# Kaizer Hill (Modi‘in), a pre-pottery neolithic a quarry site – the terraced slopes

**DOI:** 10.1371/journal.pone.0265727

**Published:** 2022-03-24

**Authors:** Naama Goren-Inbar, Anna Belfer-Cohen, Leore Grosman, Gadi Herzlinger, Aviad Agam

**Affiliations:** 1 Institute of Archaeology, The Hebrew University of Jerusalem, Jerusalem, Israel; 2 Institute of Prehistory and Protohistory, Department of Classical World and Asian Cultures, Friedrich-Alexander Universität Erlangen-Nürnberg, Erlangen, Germany; University at Buffalo - The State University of New York, UNITED STATES

## Abstract

The research of the Kaizer Hill site (the Hilltop and its Terraces), recognized as a Pre Pottery Neolithic A (PPNA) quarry site, involved studies of the rock damage associated with the quarrying activities as well as of the recovered material remains, mostly chipped stone artifacts. We present here the results of our on-site explorations (excavations, surveys and surface-collections), focusing on the findings deriving from the Terraces. Diverse rock damage patterns were identified and described, portraying systematic rock mass-exploitation through quarrying fronts, natural rock joints and fissures enlargement, drilling and chiseling. There are multiple indications that the local bedrock (Bi’na Formation, Turonian) comprising flint and limestone was quarried under a systematic quality evaluation, leaving residual flint unsuitable for exploitation. Of interest to note that nearly all of the flint artifacts excavated and collected on the Terraces were made on raw material transported from the Hilltop (Mishash Formation, Campanian), knapped in-situ, on the quarried rock surfaces of the slopes. The flint tools bear witness to intensive use involving mainly boring and drilling. The dominant tool type is the flint axe for which a variety of waste products related to its production were found in-situ, enabling the reconstruction of axe reduction sequence. Similar axes and waste products were found in many PPN sites indicating that there was a common, widely-used scheme of making flint axes during the PPN. Interestingly, besides the flint waste, there were also limestone waste products typical of the last shaping and thinning stages of axe production, indicating that limestone axes were shaped technologically similar to the flint ones, contrary to what has been assumed before. Rare findings, such as obsidian pieces, originating from much further a-field indicate ties with other PPN communities, near and/or far. Overall, this study provides unique and novel insights on Levantine PPN lifeways.

## Introduction

The environmental impact of human behavior is expressed in many ways. Some of the changes happen through direct human activities while others, under specific circumstances, happen through the impact of human activities in a roundabout way. The processes of urbanization in antiquity provided many illuminating examples of such impacts [1], yet there are much earlier, prehistoric, landscape changes (e.g., [2], so much so that their study formed a research sub-discipline, ‘landscape archaeology’ (e.g., [3–5]). Large-scale concentrations of stone artifacts are known from as early as Lower Paleolithic times [6] and references therein), yet it is only during the Pre-Pottery Neolithic B (PPNB) that landscape transformations are of such a great scale that it changed drastically the original landscape (e.g., Ramat Tamar [7, 8]). Our study of the Pre-Pottery A site of Hatoula identified landscape alteration through rock damage resulting from extensive quarrying activities [9]. Additional archaeological surveys and excavations revealed that similar rock damage, stemming from stone quarrying, exists all over the small rising hills which delineate the geographical boundary between the Israeli eastern fringes of the central Shephelah, and the western foothills of the Judean-Samarian Hills [10, 11]. Our study of Kaizer Hill further illuminates this landscape transformation phenomenon, as it is currently considered the largest known PPNA quarrying site [11].

**Fig 1:**
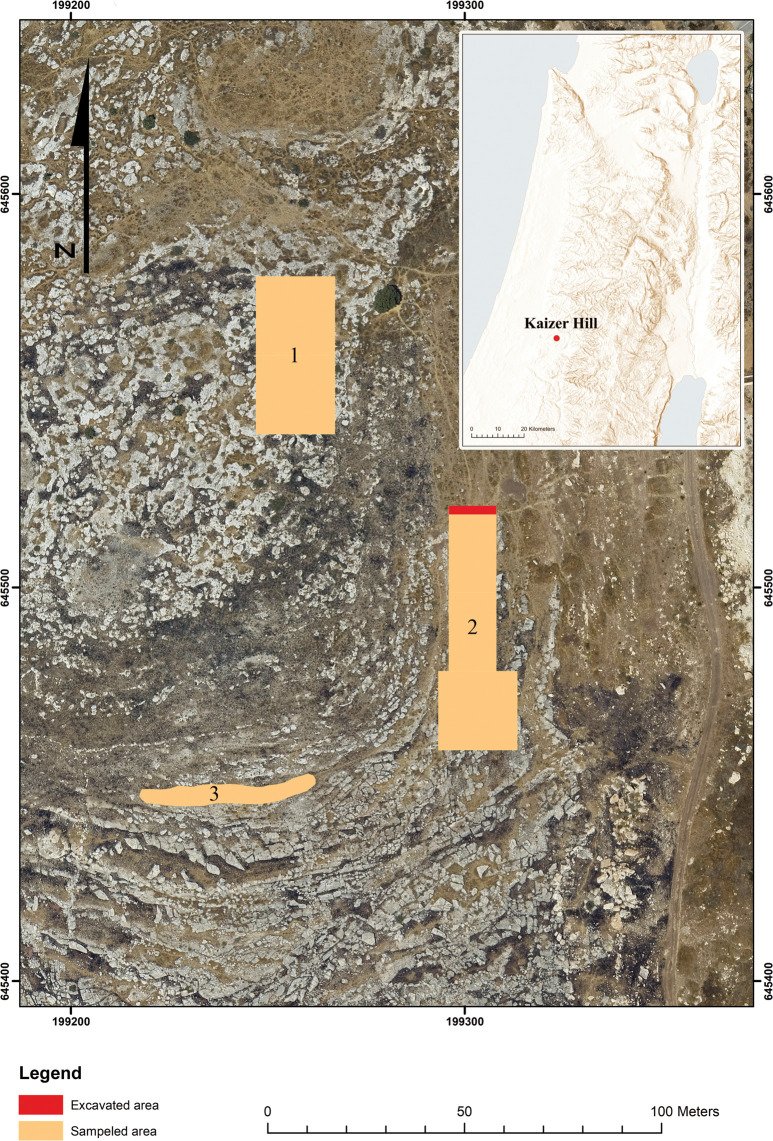
Aerial location map of Kaizer Hill PPNA quarry site. 1) Hilltop; 2) areas of artifacts surface collection, and excavation (in red); 3) surface collection–“above building”. Reprinted from ICAROS Geosystems under a CC BY license with permission from ICAROS, original copyright 2008.

Kaizer Hill is located in the southwestern outskirts of the town of Modi‘in, a city that is rapidly expanding, resulting in nearly a total destruction of the entire landscape surrounding it (including the Kaizer hill). It is situated on the northern flanks of the Anava River, on the borderline between the Shephela and the Judean Hills, some 250 m above msl (Fig 1). The hills and *wadis* of this region are characterized by eroded sedimentary rocks associated with karstic phenomena. Historical quarrying activities of the bedrock in the area are well recorded, but apparently those activities go back to much earlier times, i.e., the Neolithic period (e.g., [11–18] and references therein).

The site of Kaizer Hill underwent both archaeological surveys and excavations. Initial survey and small-scale excavations were conducted by the Israel Antiquities Authority headed by V. Zbinovich (in 2006), followed by a short-term project of the Hebrew University (in 2007–2009). Additional small-scale excavations and surveys, covering an extensive area of the hill and recording prehistoric and later periods (up to Byzantine times) were carried out by the Israel Antiquities Authority, first by Zbinovich, in 2005 and in 2010 [19] Spivak and later by Matskevich in 2015.

In the following pages we present the results of our study based on the findings of three field seasons, each of three-day duration (2007–2009), part of a field-course for Archaeology undergraduates at the Hebrew University, directed by N. Goren-Inbar and L. Grosman.

Previous studies of the Kaizer Hill site [11] revealed that it comprises a Hilltop and its Terraced Slopes. Three geological formations comprised the topography and the geology of each part. The Hilltop is made of massive chalk, the Santonian upper Menuha Formation (Fm.). This chalk is capped by a well-developed typical *caliche* (maximal thickness 1.5 m) embedded with small to large sharp-edged brown flint fragments, typical of the Upper Cretaceous Campanian Mishash Formation. The flint fragments are a residual feature of the once overlying flint-rich Mishash Fm., which has been completely eroded from Kaizer Hill, thus the flint represents a paleo-surface relict that predates the formation of the *caliche* [11, 20].

The ‘Terraced Slopes’, of the Turonian Bi’na Fm., consist mainly of well-bedded hard limestone and embedded flint which will be discussed in the following sections.

Our research of the Kaizer Hilltop included a detailed exploration of the rock damage marking, the lithic assemblage collected [11]. We also studied sampled waste piles [21] and limestone wedges [22] from the Terraces. It appears that all of these studies indicate that the quarrying activities and the associated material remains pertain to the PPNA, sometime between 11,600–10200 calBP years ago [23].

The Kaizer Hill terraced slopes are characterized by linear stone features (i.e., quarry fronts), steps, extensive surfaces of exposed bedrock and waste piles. There are many rock-damaged surfaces with varied damage patterns, attesting to extraction activities, not all of which were observed on the Kaizer Hilltop (most probably due to the differences in the bedrock nature). We have suggested that this variability is an outcome of the diverse morphology of flint [11, 21, 22]. The lowermost parts of the hill comprise giant, isolated blocks, which form a fragmented landscape; the huge blocks are ‘dotted’ with poor quality flint, clearly unusable and accordingly not included in the present discussion.

## Research objectives

The objectives of this study are twofold, aimed at a) to describe the behavioral modes and the processes that led to the creation of the unique topography of the Kaizer Hill Slopes; and b) to provide a detailed description of the lithic assemblage which was collected and excavated from the terraced slopes. Clearly, each of these studies is comprised of different methodologies and analytical procedures.

We endeavor to test the hypothesis that the particular morphology of the Terraced Slopes reflects a transformed landscape resulting from extremely large-scale limestone and flint quarrying carried out by a PPNA community, resembling the typical morphology characterizing chronologically much later quarrying localities [21]. Thus, we wish to reconstruct the quarrying strategy and techniques which were involved in the transformation of the original morphology of the slopes resulting, among other phenomena, in the formation of the quarrying fronts, hence the Terraced Slopes.

The retrieved lithic assemblage is a key component for understanding the nature and use of the PPNA quarry site and through its study we hope to gain a better understanding of the role of the lithics at the quarry and observe whether it differs from other PPNA lithic assemblages originating in other types of sites. A component of this analysis is the description and discussion of the bifacial tools and its particular characteristics [11, 24] and provide possible explanations concerning their role in the life-history of the quarry. Additionally, special attention was given to a particular type of biface waste products resembling *tranchet* waste [25]. We have conducted an experimental project focusing on the bifacial artifacts, based on the knowledge gained from the study of the Kaizer site artifacts. It involved production of bifaces similar to the latter and, attempting to imitate the final products as those existing in the Kaizer assemblage and to examine their waste products.

### What was quarried and how

It seems that the quarrying activities were aimed toward procurement of both flint and limestone.

**Fig 2:**
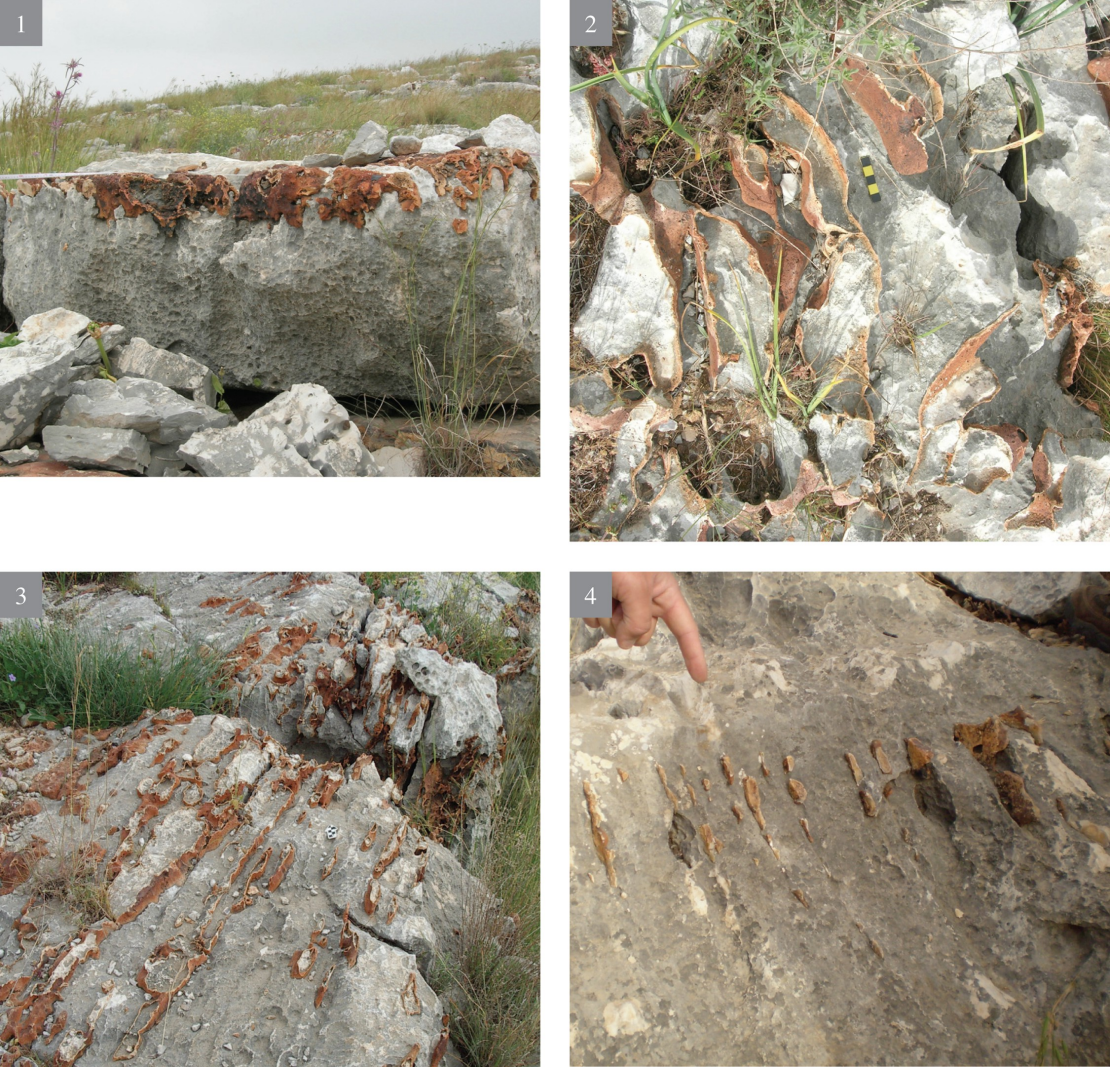
Bi’na formation—appearance and morphology (1–4).

**Fig 3:**
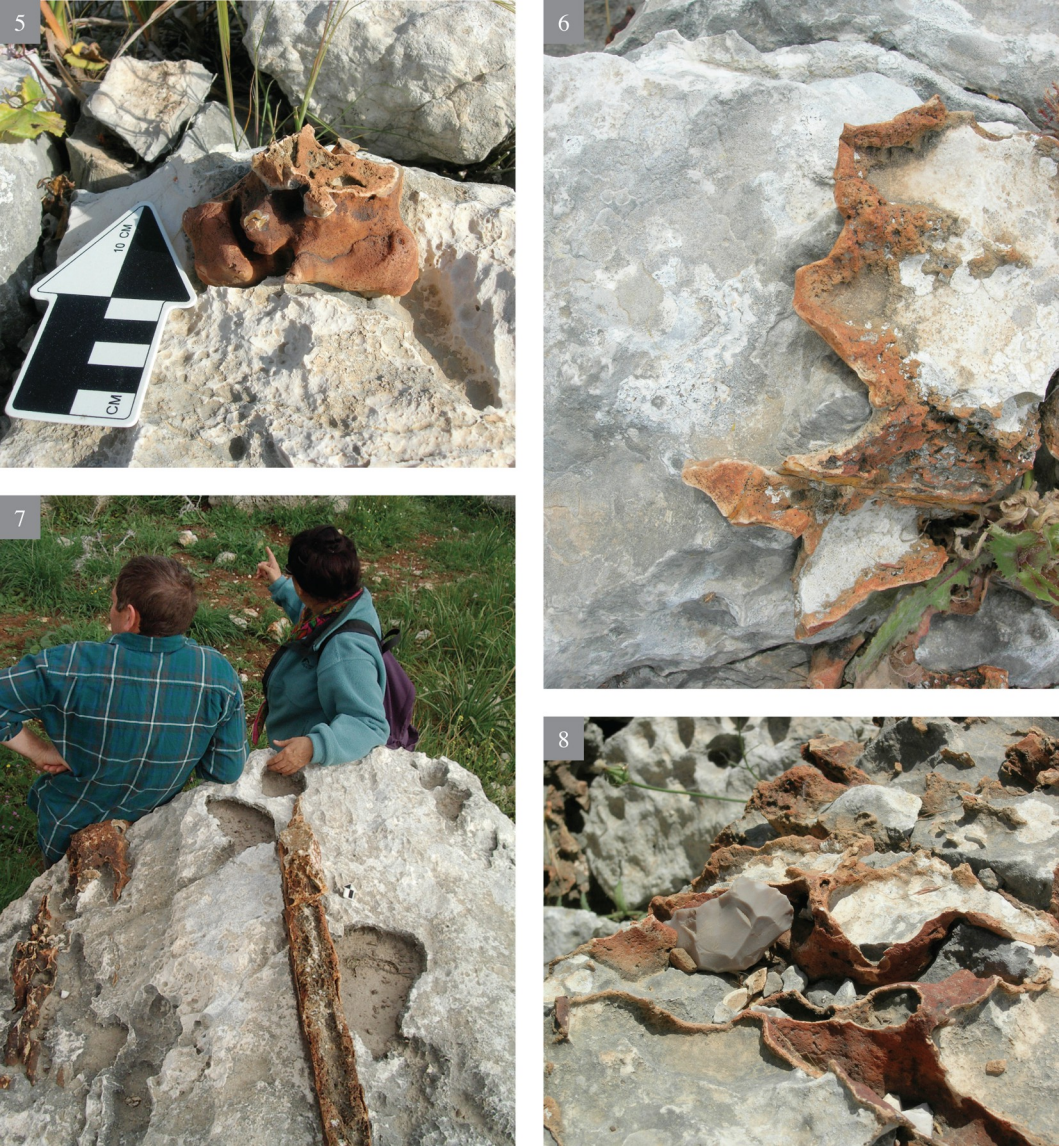
Bi’na formation—appearance and morphology (5–8).

The flint visible all over the slopes varies greatly in volume, shape, as well as in the variety of rock stratigraphic positions. As a rule, such flint appearance is extremely rare, both in rock sedimentary formations of Israel and in the Bi’na Fm. Most probably it is due to its origins from water that contained soluble silica (SiO_2_) in super saturation, and which spread irregularly over the surrounding limestone, filling the hard rock fissures. Most of the exposed flint is extremely cortical, red brown in color, at times very thin, amorphous and thus unsuitable (i.e., of bad quality) for knapping. Being actually solidified ‘liquid’, the flint on the slopes appears in many forms, from a thin layer of a few mm thick, to thick layers; from vertically bedded ‘seams’ to horizontally bedded layers, etc., (Figs 2 and 3), all dictated by the pre-existing geological stratigraphy and structure of the rock beddings and its ‘geometry’. The quality of the flint observed *in situ* most probably indicates that it is actually residual “leftovers”, due to its bad knapping quality.

**Fig 4:**
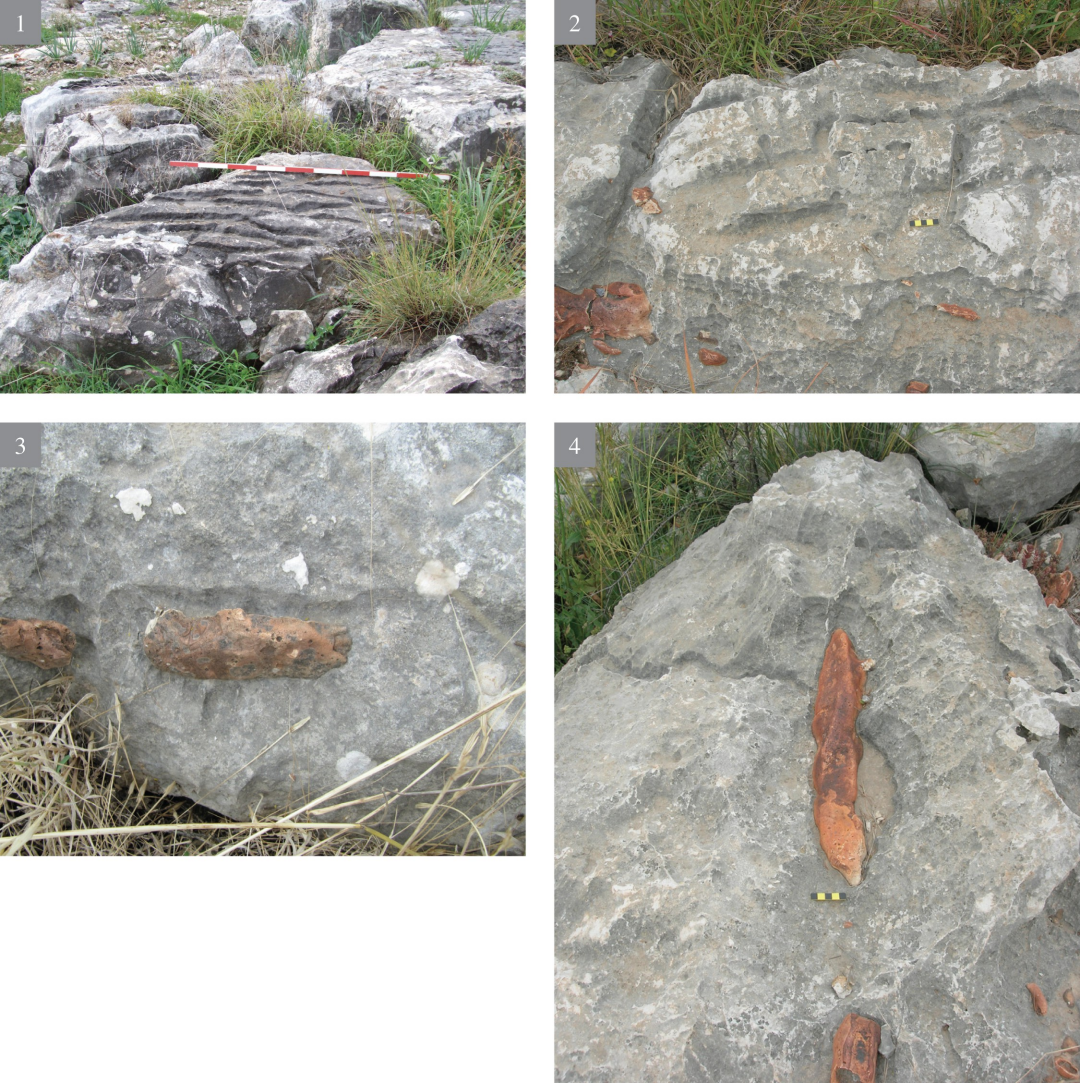
Rock damage resulting from flint quarrying of the Bi’na Formation bedrock (voids) and different types of quarrying marks (1–4).

**Fig 5:**
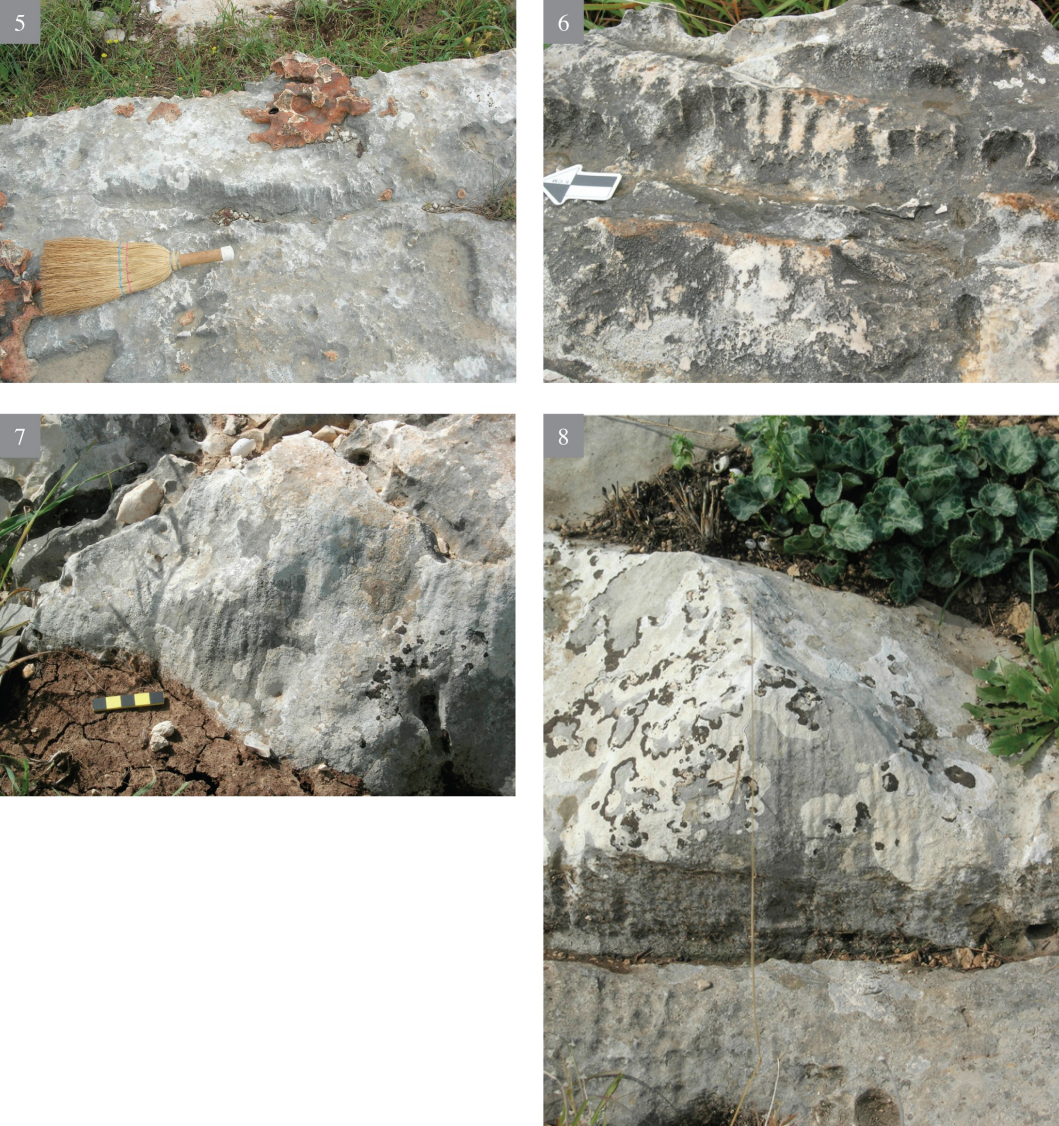
Rock damage resulting from flint quarrying of the Bi’na Formation bedrock (voids) and different types of quarrying marks (5–8).

**Fig 6:**
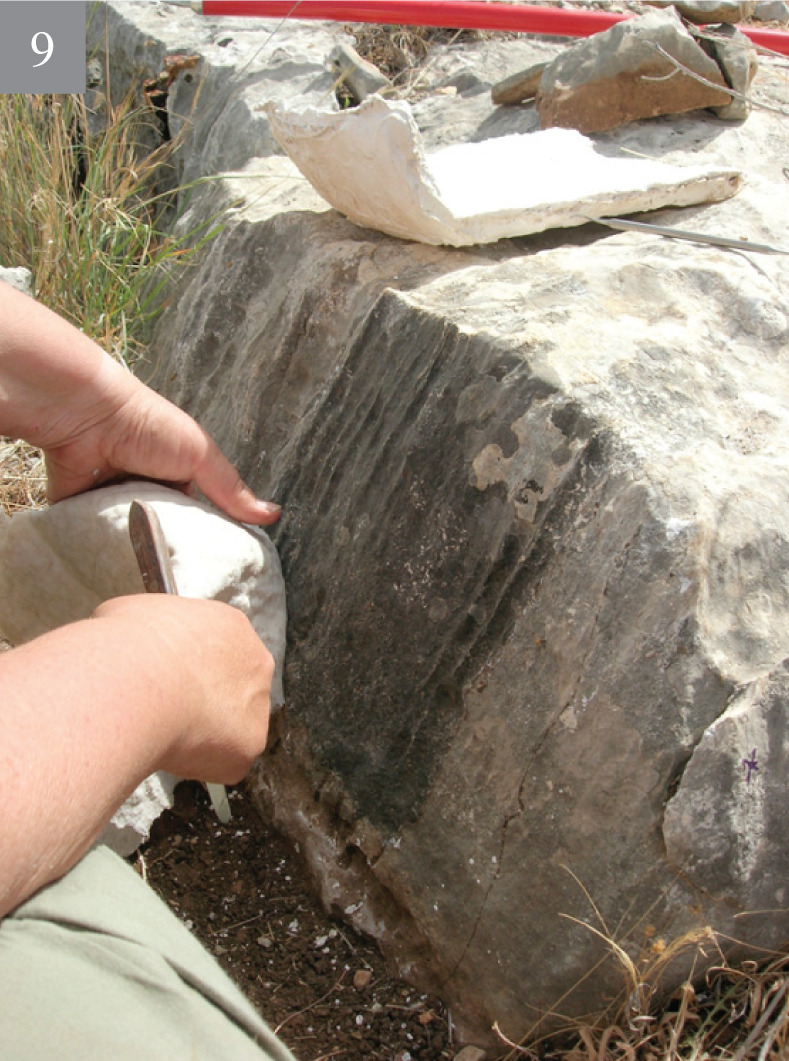
Rock damage resulting from flint quarrying of the Bi’na Formation bedrock (voids) and different types of quarrying marks (9).

The search for good quality flint is indicated by two differing types of rock damage markings visible on the bedrock: **a)** marks of the quarrying attempts to expose the flint, and once it was rejected, leaving the exposed flint *in situ*. **b)** rock damage markings, called here “voids”, testifying of flint extraction (Figs 4–6). The latter are most significant as they provide an estimation of the shape and size of the extracted flint nodules. They also testify for the great variability in shape and volume of the Bi’na Fm. flint (henceforth Bi’na flint). Judging by the extensive rock damage, the investment in searching for good quality flint, its exposure and extraction was intense. Suitable flint for knapping is totally absent from the terraces. It is only the ‘voids’ and the large array of quarrying marks which provide the data on the local flint extraction.

Besides the rock damage patterns, on-site flint extraction and production is also indicated by the presence of knapped flint and limestone artifacts on the surface, as well as of those which were excavated. Our hypothesis is that a substantial work on the rocks was carried out by bifacial tools which form the most frequent tool category at the site (both on the hilltop and on the terraces). The quarrying procedures included drillings, chiseling (i.e., a series of parallel longitudinal removals of rock ‘stripes’ of variety of widths), fissure enlargement (scars resulting from direct removal of limestone chunks, as well as through use of wedges and large hammerstones), etc. The width of the chiseling varied, as evident in the differing width and shape (circular, ovoid, straight) of the stone damage markings, which clearly necessitated several types of instruments (Figs 4–6).

The quarrying comprised complex multistage procedures, which involved two major types of activities. The first was concerned with the exposure of large rock surfaces in order to reveal the geological bedding of the limestone where flint occurs, the location and extraction of knappable flint.

**Fig 7:**
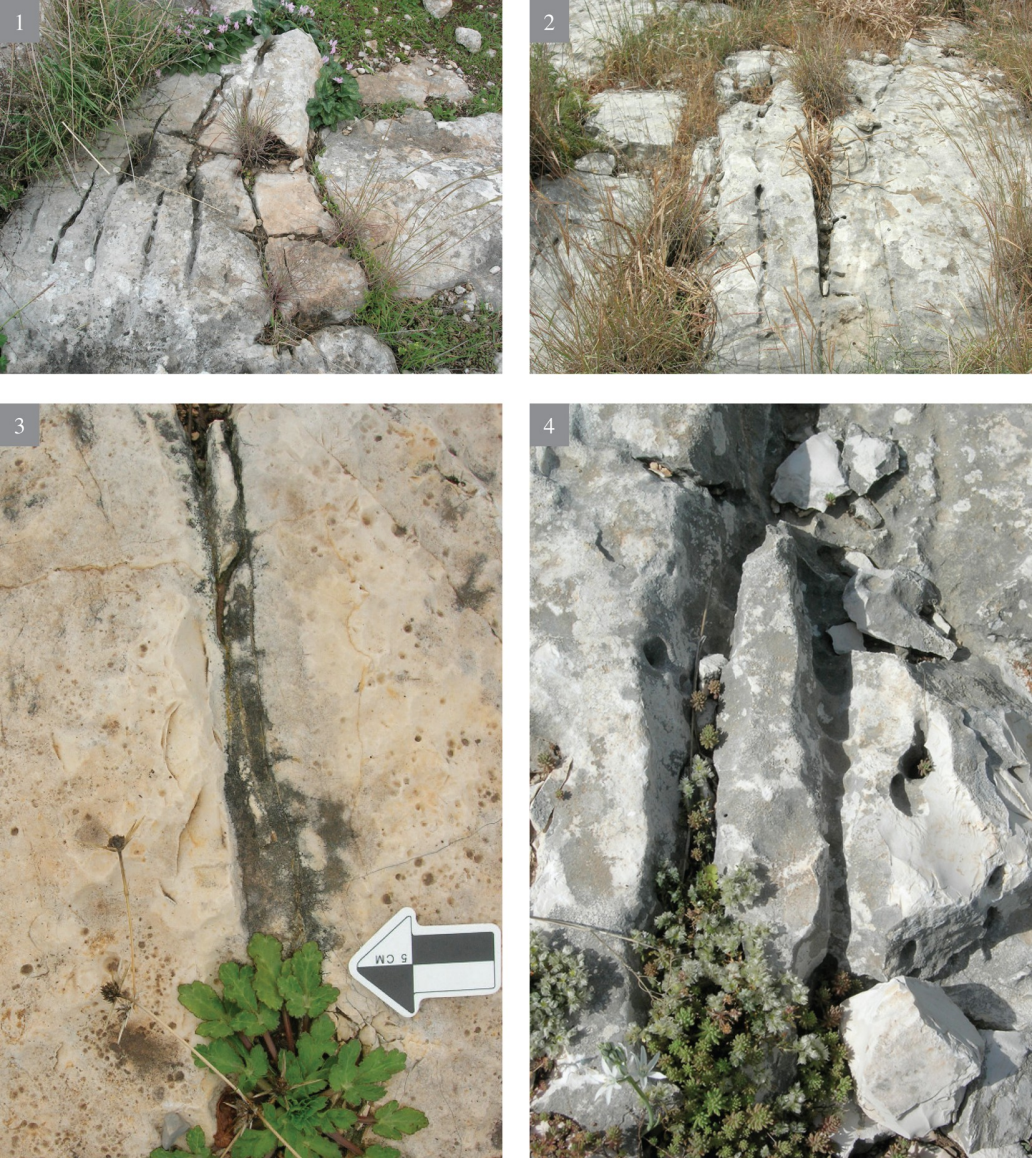
Rock markings indicating limestone quarrying. Drilling (no. 1–2); fissure widening (no. 2–4); flaking (no.3).

The second type of quarrying was intended for extraction of the limestone. Here, a very hard and dense limestone, without irregularities or flint embedded in it was selected for extraction (Fig 7). There are few indications for limestone extraction, perhaps intended as raw material to produce bifaces. Though their manufacturing technology is only partially known, PPNA limestone axes are reported from various sites, their appearance continuing unto the PPNB [24, 26]. Unfortunately, although there is clear evidence for limestone quarrying, its paucity among the finished lithic products precludes a detailed discussion (but see below).

## Methodology

**Fig 8:**
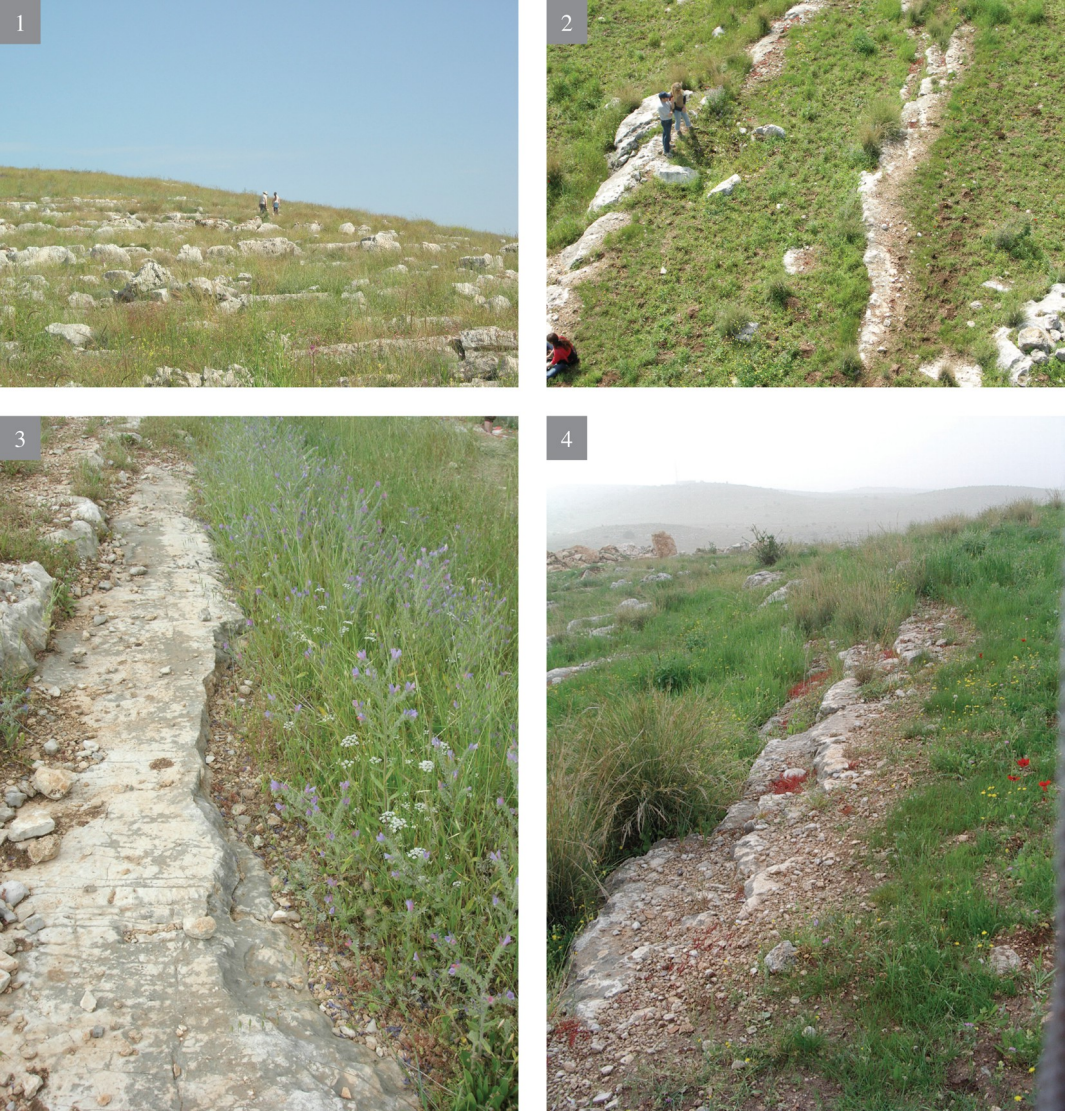
The Terraced slopes of Kaizer Hill; 1) looking uphill; 2–3) the quarrying fronts (linear lines) viewed from the air; 4) the quarrying fronts.

**Fig 9:**
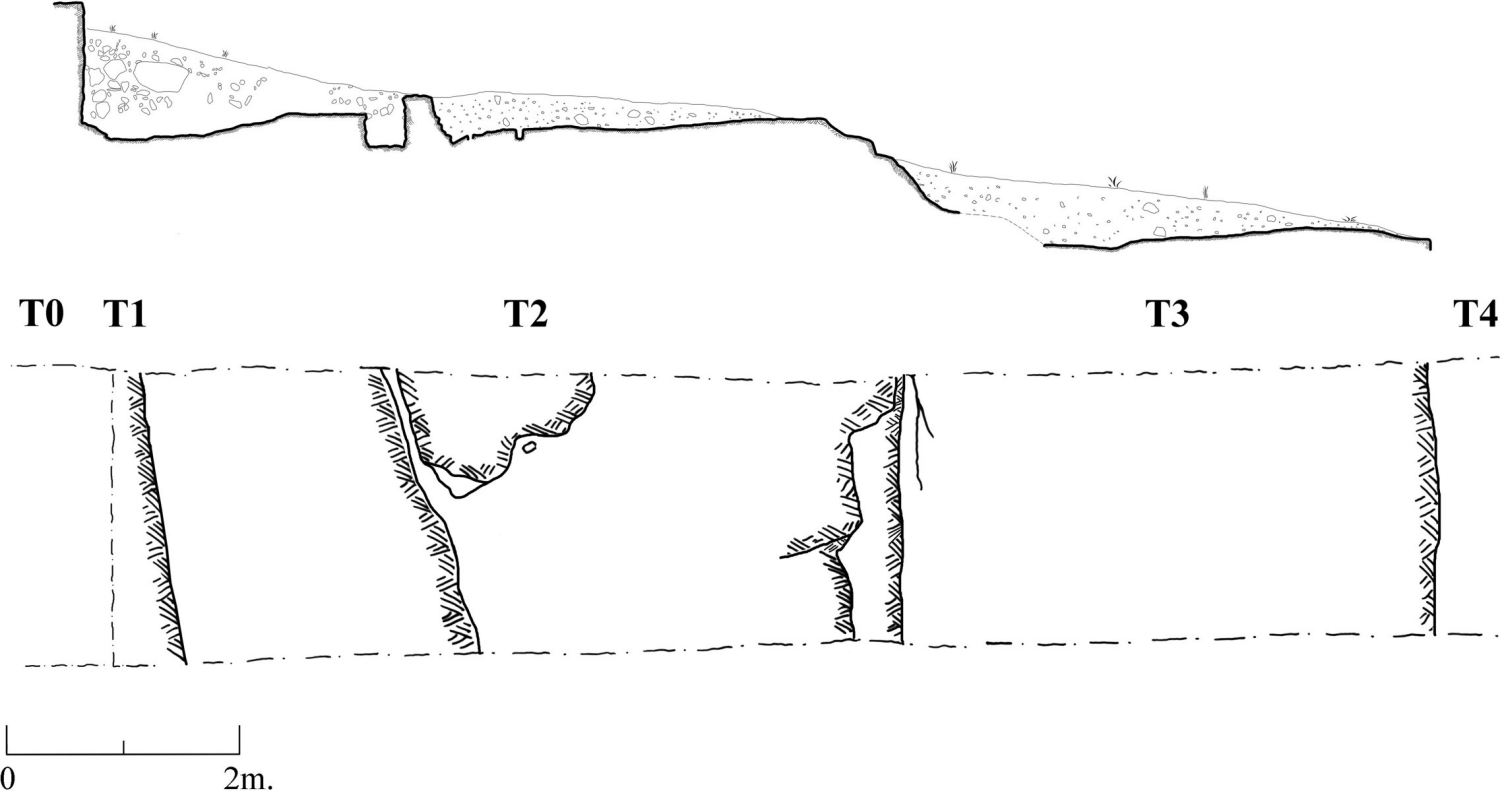
The excavation trench. Top: cross-section along the quarrying fronts T1-T4; Bottom: plan view of the quarrying fronts.

**Fig 10:**
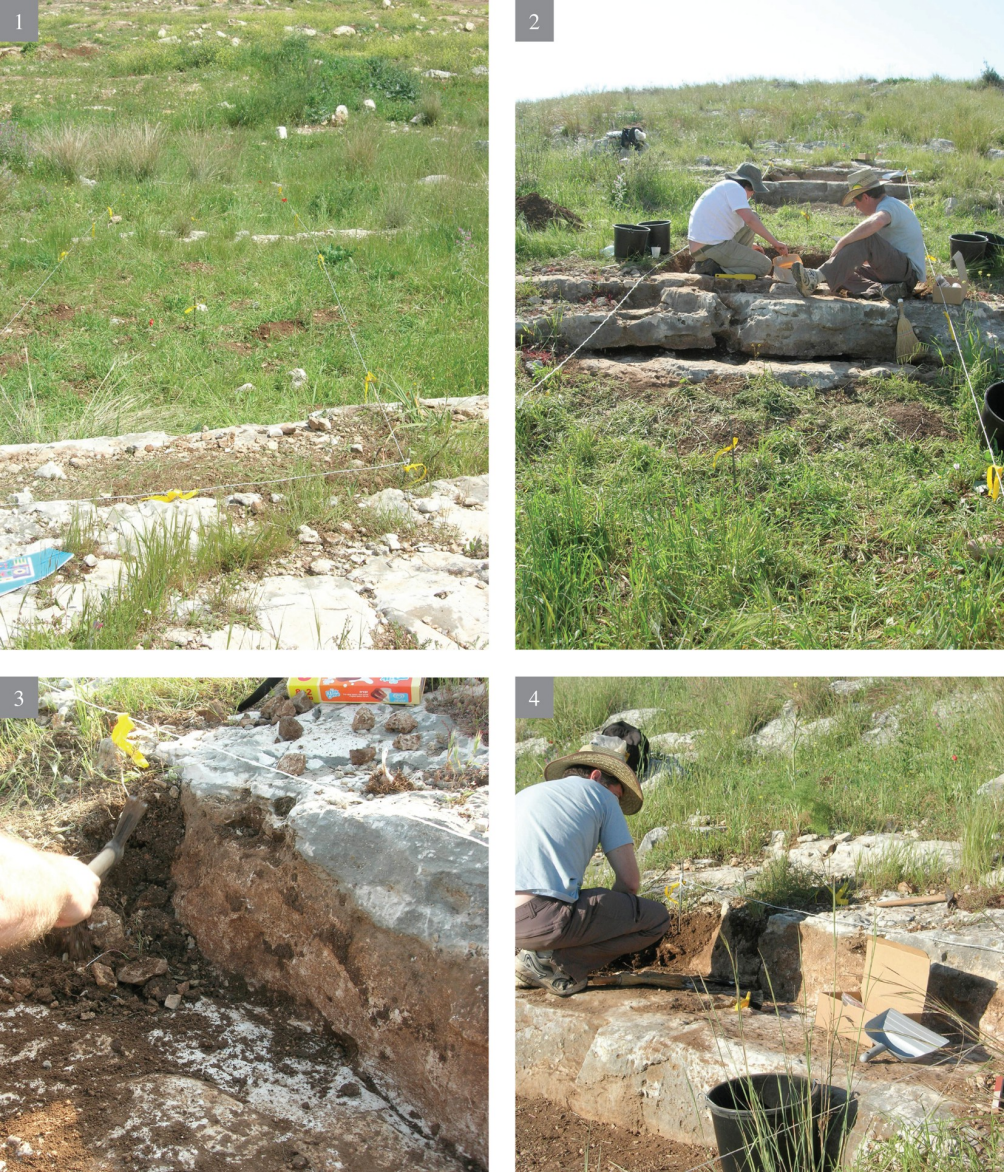
The excavation trench. (1–4) preparing the excavation.

**Fig 11:**
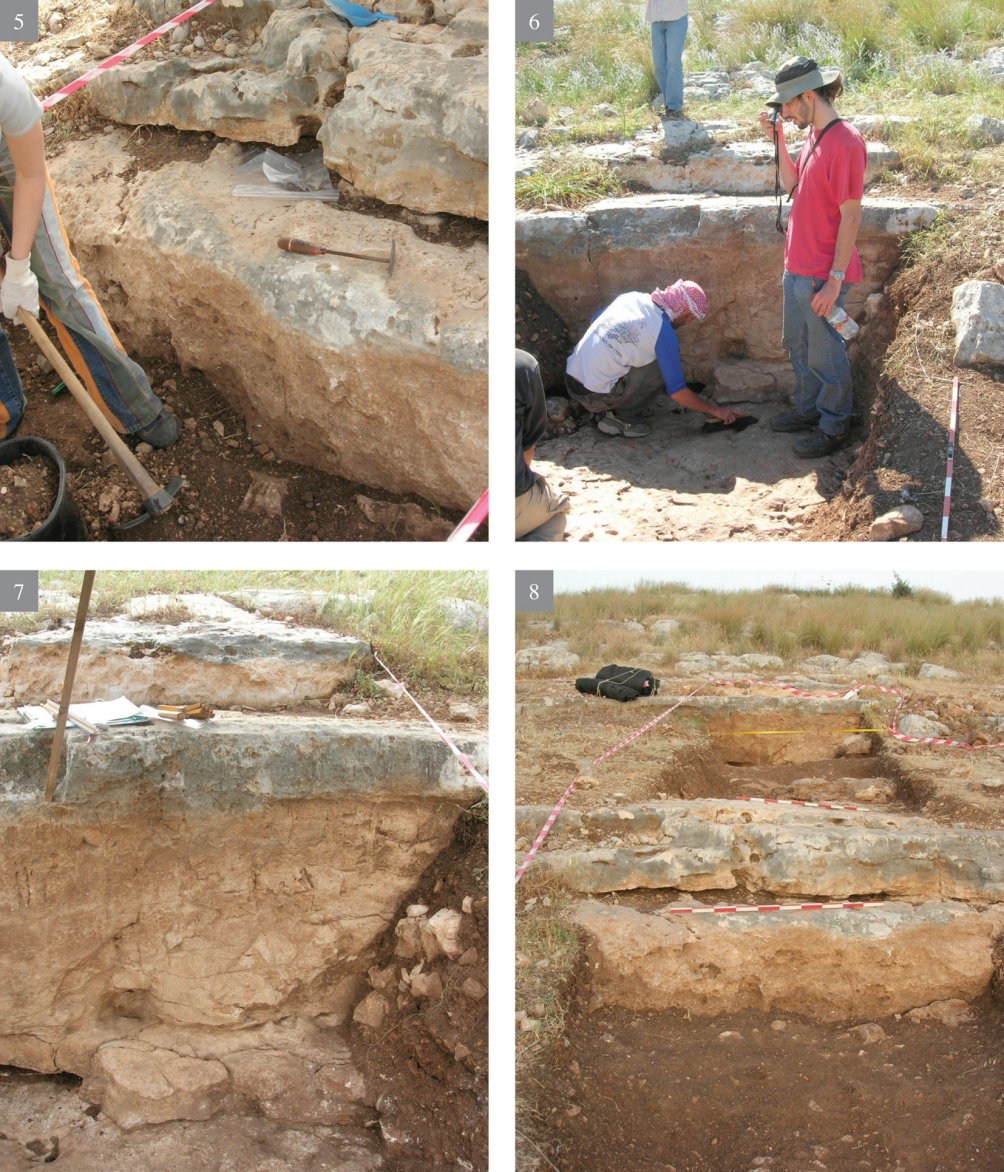
The excavation trench. (5–8) exposure of quarrying fronts.

**Fig 12:**
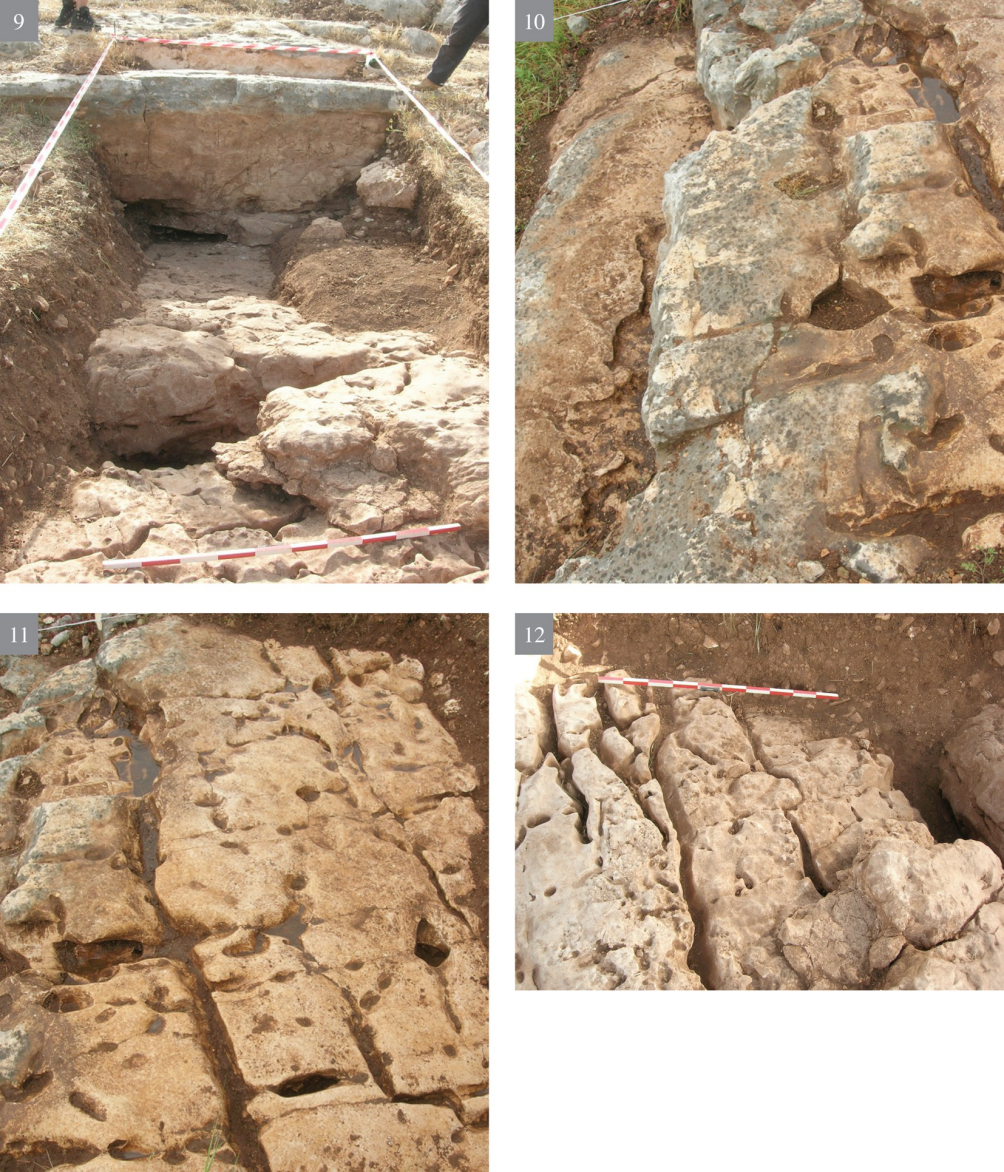
The excavation trench. (9–12) the quarrying fronts and the exploited surfaces.

As the size of the PPNA quarry site of Kaizer Hill is very large, covering some 10,000 m^2^, we have decided on documenting and sampling in detail several localities at the quarry:

The selected areas include the Hilltop [11], several waste piles on the southeastern flanks [21] and the eastern flanks of the hill, where the slopes are delineated by stony terraces. The detailed study of the terraced slopes (Fig 8) entailed the following:

1. **A** reconnaissance survey of the slopes and inspection of the different rock morphologies, flint quality and rock damage patterns.
2. Excavation of a trench (2x12 m) cutting the quarrying fronts (T0-T4) perpendicularly along five terraces (quarrying fronts) and descending topographically, each terrace separated from the other by a lower quarrying front. Fig 9 is a map view of the excavated trench and its cross section, showing the differences between the excavated terraces. As quarrying followed the various features and the stratification of the limestone bedrock, each excavated terrace (quarrying front) reached a different depth, with a maximal depth of 140.9 cm in T2 (Fig 9). Interestingly, the exposure of bedrock revealed that the Bi’na limestone was not homogeneous nor regular in all places. Accordingly, the Neolithic quarrymen left some of the exposed bedrock found to be of no potential (i.e., revealing poor quality flint and limestone) (Figs 10–13; 11:7 and 12:9). On the other hand, the bedrock rich in flint was extensively quarried as evidenced by the depth of the quarrying fronts and by the efforts to enlarge fissures as part of the general quarrying method (Figs 12: 12 and 13:13). The bedrock portrays an array of rock damage features, including quarrying fronts, fully exposed to their maximal height and damage marks (e.g., flaking scars) including those associated with widening of natural rock fissures (Figs 10–13; 11:6, 8 and 12: 9). Excavation permits were given by the Israel Antiquities Authority. These include permit No. G-36/2007, G-29/2008 and G-41/2009 for the years 2007–2009 respectively. No permits were required for the described study, which compiled with the relevant regulations. The lithic artifacts are deposited in the Department of Prehistory, The Hebrew university of Jerusalem, at Mt. Scopus campus, Jerusalem, Israel.

**Fig 13:**
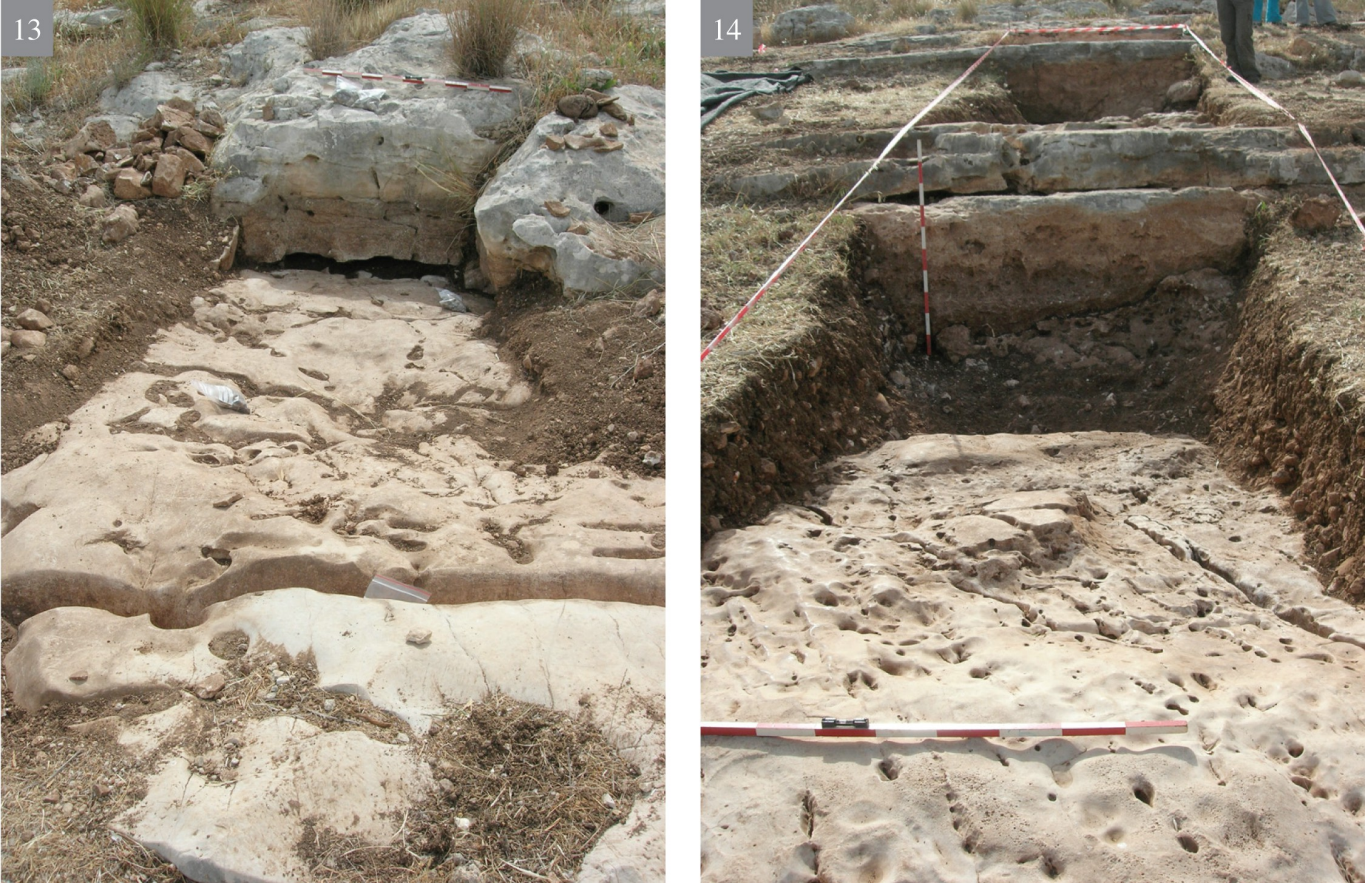
The excavation trench. (13–14) quarrying fronts, exploited surfaces and an enlarged fissure.
  The excavated deposit (maximal fill depth is in the order of 80 cm) was composed of fine-grained sediments of reddish color, flint artifacts and limestone waste products. There were no large limestone flakes and/or blocks, because of the quarrying strategy which included the removal of this component to spoil piles sorted by the different sizes of the waste products [21]. The excavated sediments were sieved, and the finds (flint and limestone) were separated per terrace and per type of raw material. While all the flint component was retained, the limestone component underwent selection as it was impossible to keep and store all the artifacts due to their great quantity. Thus, only limestone wedges [22] and a selection of other limestone artifacts, mostly of small size, were kept for further studies.
3. A surface collection of lithics from areas located adjacent to the excavated trench (Fig 1). The size of this sampled area is 24 m^2^ (2x12m) + 2x (12x20 m) + (20x20 m) = 904 m^2^. The collection was total and systematic, including items smaller than 2 cm (i.e., chips).
4. A surface collection (196 m^2^) of all lithics was carried out above the stone “structure” (made of residual blocks and stones of the quarry, at first assumed to be intentional, but proven to be natural) (Fig 1). This area is termed herewith as “Above building”.
5. Two straight line transects (A and B) were made beginning on the highest topography of Kaizer Hill and descending all the way down along the slopes (Fig 14).
6. Sixteen units of exposed bedrock bearing rock damage markings were selected for analysis. Transect A passes through Units 6 and 12 while transect B passes through unit 10 (Fig 15 and S1 Fig 1 in S1 File). The recording of the 16 units involved a detailed description of the observed rock damage patterns (and see below: results of study).
7. As Kaizer Hill was planned to be integrated within the boundary of Modi‘in city and hence destroyed, we made an effort to produce several casts of different rock damage types in order to facilitate their analysis in the future.

**Fig 14:**
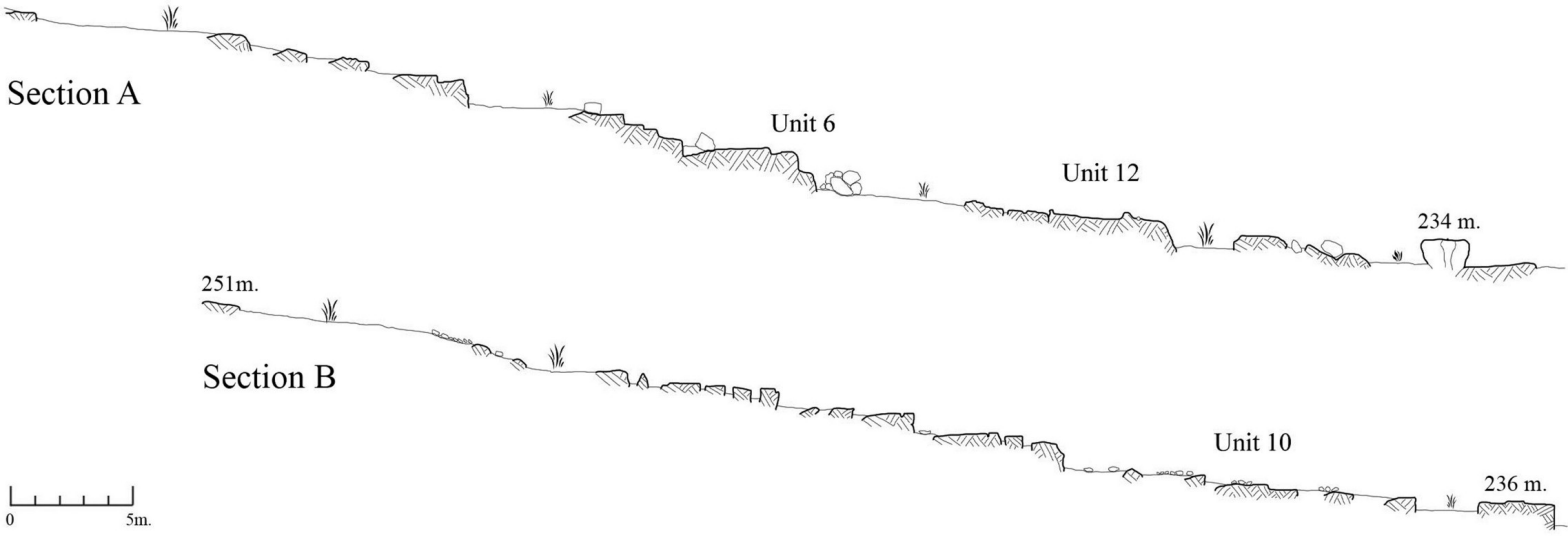
The two transects along the slopes of the hill (A, B).

**Fig 15:**
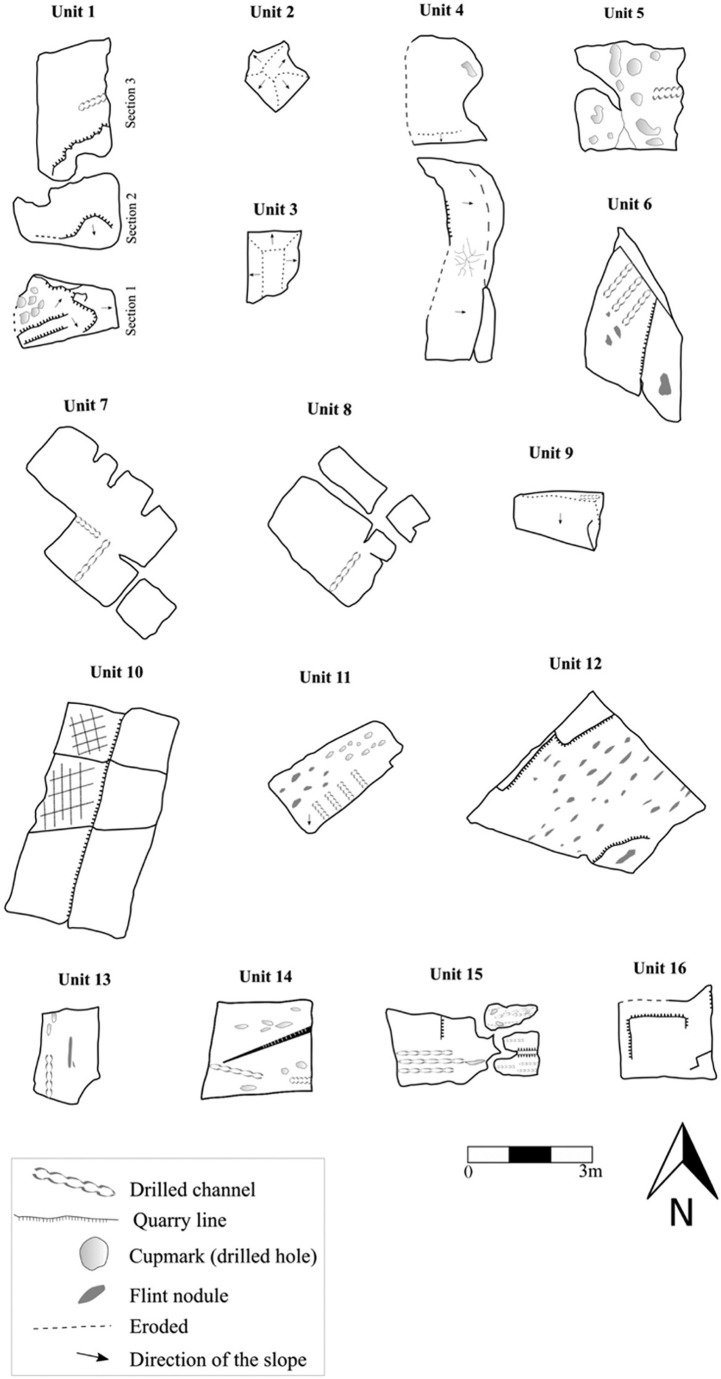
The rock damaged 16 units.

### Analytical procedures

The lithic study of the Kaizer Terraces assemblage was carried out in accordance with the conventional techno-typological classification, pertinent and applied to other Levantine PPN assemblages (Jericho: [25]; Netiv Hagdud: [27]; Gilgal: [28]; Gesher: [29]). A detailed description of all the tools from all four assemblages is provided in S1 Text.

#### 3D geomertric morphometrics

A 3D homologous landmarks-based geometric morphometric shape analysis was conducted on 53 complete tools in order to quantitatively describe, analyze, and facilitate future comparison of the biface assemblage. The tools originate in two different areas: 19 were collected on the Hilltop, while the other 34 were retrieved from the Terraces. The latter group can be further divided into 22 artifacts collected on the surface and 12 excavated in situ within the trenches. The digital 3D models of these tools as well as their positioned 3D landmarks data are available online (see data availability statement). All analytical geometric and statistical analyses and procedures were conducted using AGMT3-D v3.1 and SAS JMP v14.0 following standard protocol [30–31].

The analysis was conducted on 3D digital models of the artifacts obtained by 3D surface scanning using Shining 3D Einscan-SP structured light scanner. Next, the models were subjected to the software’s standard automatic positioning procedure which positioned them consistently in space so that the plane, roughly equivalent to the intersection between the two faces of the tool, would be placed parallel to the XY plane. Manual confirmation of positioning consistency was performed, and if necessary, artifacts were rotated in 90 degrees steps so that the distal working edge would be placed on the positive side of the Y axis. After all items were positioned, a dense grid of 20X20 landmarks was projected onto each face, resulting in 800 indexed 3D landmarks accurately representing the volumetric configuration of each tool, allowing their subsequent comparison. The landmarks coordinate data of all items were compiled into a single dataset which in turn was subjected to generalized Procrustes analysis (GPA), used to filter out differences in translation, rotation and scale, leaving only morphological related differences. Lastly, principal component analysis (PCA) was performed on the GPA modified data. It is used to reduce data dimensionality, measure the amount of shape variability in the sample and detect its main axes. Thus, it provides a number of components, each reflecting a specific shape trend, that is, a mutual change in the values of a number of homologous landmarks. Alongside the general shape trends, additional morphometric attributes measured in the framework of this analysis include: volume (cm^3^), surface area (cm^2^), bilateral and bifacial asymmetries, total lateral edge curvature, and total lateral edge irregularities in planform and section views [31]. Additional analyses performed on the data include agglomerative hierarchical clustering for exploring potential morphological groups within the sample and the non-parametric Wilcoxon rank-sum test for testing the statistical significance of morphometric differences between sub-samples.

#### The flint raw material

The surroundings of the site were surveyed in search for potential flint sources. We explored both the immediate vicinity of the site, and further afield, up to a distance of ~15 km away from Kaizer Hill. All sources were mapped and sampled.

**Fig 16:**
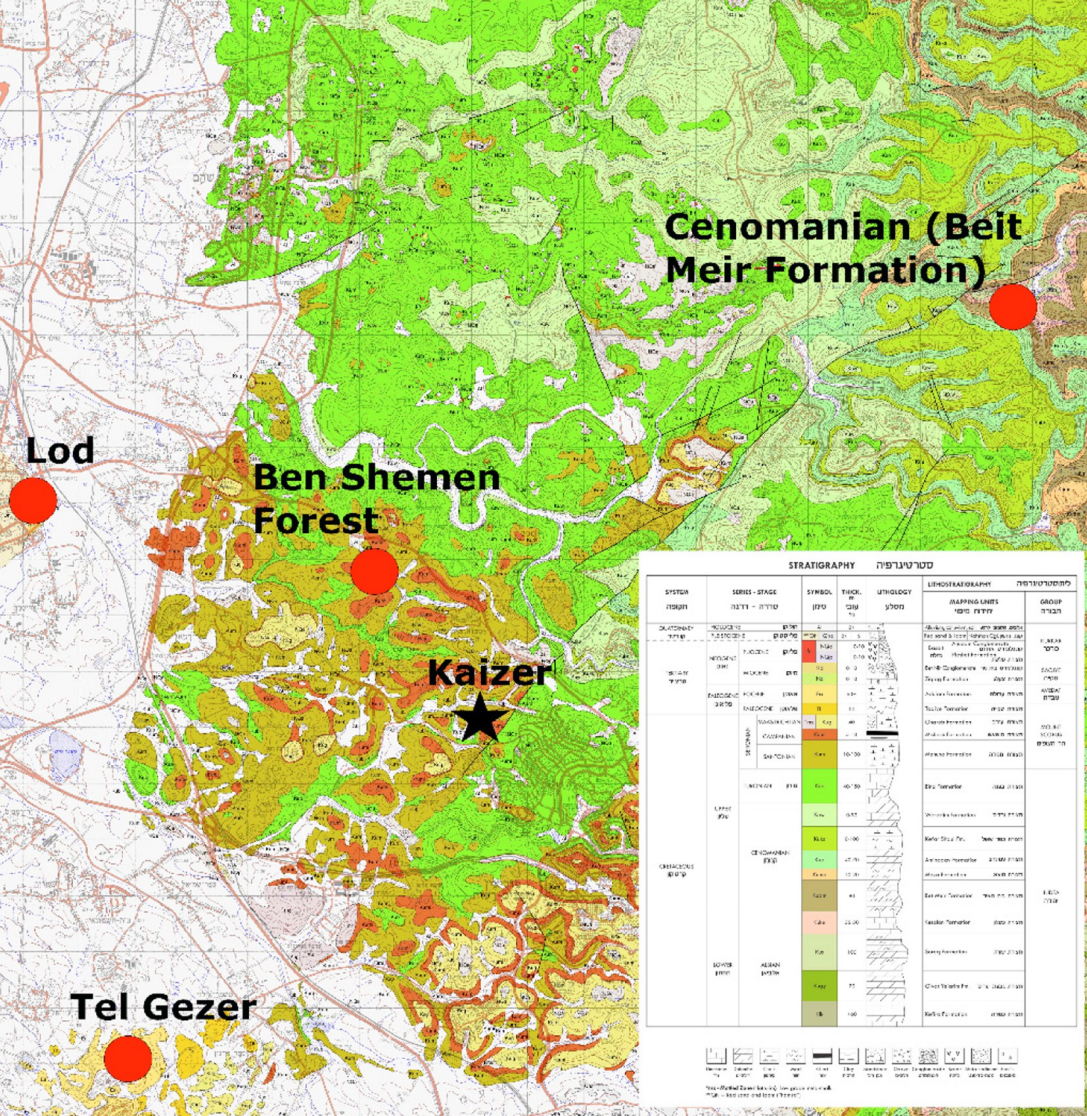
The geologic setting of Kaizer Hill (following [20]). Note the interchanging Campanian and Turonian outcrops in the immediate vicinity of the site. Reprinted from Yechieli 2008, under a CC BY license. It is taken from the Geological survey of Israel website (public domain), with permission from Dr. Amit Mushkin of the Geological survey of Israel, original copyright 2021.

The following description is of the sites’ geological settings. Turonian and Campanian outcrops, forming the Kaizer Hill, are also present as rich outcrops in the surroundings of the site [20], making such flints highly available in the area (Fig 16 and Table 1). Cenomanian exposures of the Bet Meir Fm. are located some 12–13 km to the north-east of Kaizer Hill (not surveyed for this study); Eocene sources of the Adulam Fm. are ~ 9–10 km south-west of the site in Lod and Tel Gezer (sources 5–8 in Table 1).

**Table 1:** The surveyed geologic sources.

	Name	ID	Coordinates	Age	Formation	Distance from Kaizer (in km)	Primary / secondary
1	Zaglembie Martyrs (Memorial)	ZM	31°56’14.53"N, 34°59’1.06"E	Campanian	Mishash and/or Menuha Fm.	3.14	secondary
2	Ben Shemen	BS	31°56’42.06"N, 34°58’9.07"E	Campanian	Mishash	4.44	secondary
3	Ben-Shemen West	BSW	31°56’48.19"N, 34°56’17.15"E	Campanian	Mishash	6.56	primary
4	Ben-Shemen Center	BSC	31°56’43.39"N, 34°56’16.67"E	Campanian	Mishash	6.47	primary + secondary
5	Tel Gezer North	TGN	31°51’35.71"N, 34°55’10.67"E	Eocene	Adulam	8.82	primary
6	Tel Gezer East	TGE	31°51’35.66"N, 34°55’10.77"E	Eocene	Adulam	8.82	primary + secondary
7	Tel Gezer 1	TG1	31°51’35.60"N, 34°55’10.68"E	Eocene	Adulam	8.89	secondary
8	Lod	L	31°57’13.94"N, 34°54’19.73"E	Eocene	Adulam	9.53	secondary

The high frequency of geologic flint sources in the vicinity of Kaizer Hill, especially the primary sources, increase the likelihood of finding additional flint quarries in the area. It is therefore our view that the flint quarry of Kaizer Hill, in addition to those in Hatula [9] are but a part of a large-scale PPNA phenomenon, with intense activity oriented towards the acquisition of flint as well as limestone.

The flint of the chipped stone assemblage recovered on-site was assigned to four potential geologic origins, based on visually observed traits: Upper Cretaceous Turonian flint of the Bi’na Fm., Upper Cretaceous Campanian flint of the Mishash Fm., possibly Eocene flint, possibly Cenomanian flint, and flint pieces which are too worn, burnt or small to be assigned to any of the above. All samples were weighed, as groups, by geologic origins.

The assignment to potential geologic origins was based on macroscopic traits considered indicative for such classification. Campanian Mishash flint was identified based on its characteristic deep brown color, and its brecciated texture. The Turonian Bi’na flint was identified based on its resemblance to the Turonian nodules collected from the outcrops of Kaizer slopes—amorphous pieces, with a coarse texture, and relatively low silica content. Flint pieces were tentatively assigned to Eocene or Cenomanian origins if they presented traits which resemble geologic samples collected from such sources, e.g., assignments to an Eocene origin were based on similarities with flint pieces observed in deposits of Eocene flint near the city of Lod and near Tel Gezer. Assignment to a Cenomanian origin was based on the identification of light brown opaque homogenous pieces typical of Cenomanian sources of the Beit Meir Fm. [32, 33]. Still, the classification to both, Eocene and Cenomanian origins, should be treated cautiously, as they are yet to be tested using petrography.

Petrographic thin sections of six Turonian and three Campanian samples were produced at the Department of Earth and Environmental Sciences at the Deichmann Rock Mechanics Laboratory at Ben-Gurion University. The thin sections were analyzed at the petrographic lab at Tel Aviv University, by optical microscopy, in both plane-polarized and cross-polarized light, using a ZEISS Axio Scope.A1 Polarized Light Microscope.

## Results

The results of the various studies are presented according to the field activities described above: **(1)** The transects; (**2**) The rock damage patterns of the 16 units; **3**) the lithic assemblage deriving from collection and excavation.

### The transects

Since the quarry is huge and its topography and rock types are varied, we opted to express these diversities through two parallel topographic transects from high to low topography along the slopes (Fig 14) and a selection of 16 rock units with rock damage surfaces whose features were described in detail. The transects go over three of the 16 selected rock units (see below).

### Rock damage

The 16 marked rock units portray an array of different quarrying features: quarrying, fissure widening, drilling, exposure of bedded flint and the leftovers when raw material quality is inappropriate (Fig 15 and S1 Text and S1 Fig 1–7 in S1 File). Table 2 presents a description of the markings per unit (see supporting information for notes).

**Table 2:** Size and rock damage patterns of the 16 selected units.

Unit	Length (m)	Width (m)	Height (m)	Type	Elevation Above msl
Unit 1	7		0.8	Mainly 1 & 2	
Unit 2		1.30	0.9	Mainly 4	
Unit 3		1.5	0.8	Mainly 1	
Unit 4	12		1.	1	
Unit 5	3	2.5	1	Mainly 4	209
Unit 6	3		1	Mainly 1	243
Unit 7	7	2.5	0.5	Mainly 2	
Unit 8	7	2.5	0.45	Mainly 2	243
Unit 9	2.2	1.1	0.6		239
Unit 10	5.5	2.4	0.5	Mainly 2	248
Unit 11	2.5	1.4			239
Unit 12	4.5	4	1.1		239
Unit 13	2	1.7	1.1		239
Unit 14	2.7	2.1	0.4		242
Unit 15	3.4	1.7	0.7	Mainly 2, small & narrow	
Unit 16	2.3	2.2	0.6		242

### The finds

Detailed analyses were carried out as regards the main raw material, the flint including its various geological sources, aiming to characterize the assemblage as well as to reconstruct procurement patterns in-and-out of the quarry.

While the flint component underwent a detailed typo-technological analysis, the study of limestone finds was rather limited, pertaining only to biface production debitage (and see below).

#### Raw materials

**Fig 17:**
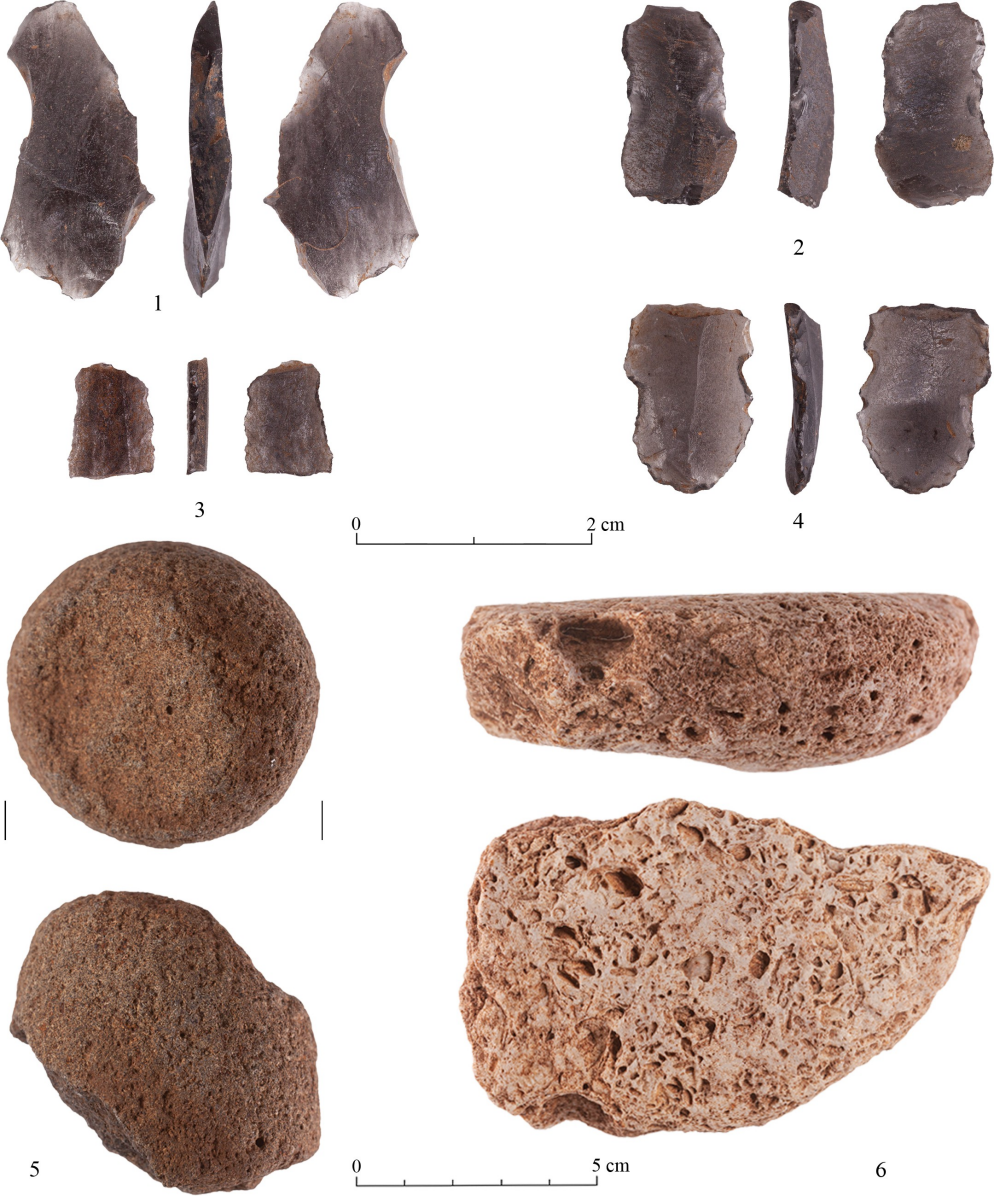
Rare raw materials: 1–4) obsidian—broken blades; 5) basalt—broken pestle; 6) silicified limestone—broken grinding stone.

The Kaizer Terrace slopes lithic assemblage includes three rare rock types minimally represented. These are basalt (N = 1), silicified limestone (quartzolite) (N = 1) and obsidian (N = 4). The basalt item is a broken pestle (Fig 17: 5) and the quartzolite is a broken grinding tool (Fig 17: 6). While the origin of the basalt is unknown, that of the quartzolite is associated with the Bi’na Fm., though not encountered at Kaizer Hill or its vicinity. This rock type appears in the foothill of the Judean Mountains as a compact, stratified layer. It is composed of quartz crystals and voids of fragmented fossils and its grinding properties are similar to those of basalt. During the formation process (the silicification) of quartzolite, the interior pressure in the biogenic limestone caused lateral fissuring on the upper part of the layer (3–7 cm thick), resulting in fragments with flat surfaces suitable for grinding tools (identified by Z. Levi).

The obsidian is represented by four small blade/bladelet fragments (Fig 17:1–4). Although these artifacts did not undergo a chemical analysis, their structural and visual features are characteristic in general of the obsidian source of Göllü Dağ, and perhaps one fragment (Fig 17: 4) could be assigned specifically to East Göllü Dağ (Kayirli) [34, 35].

#### Flint: Analysis of the flint types

**Fig 18:**
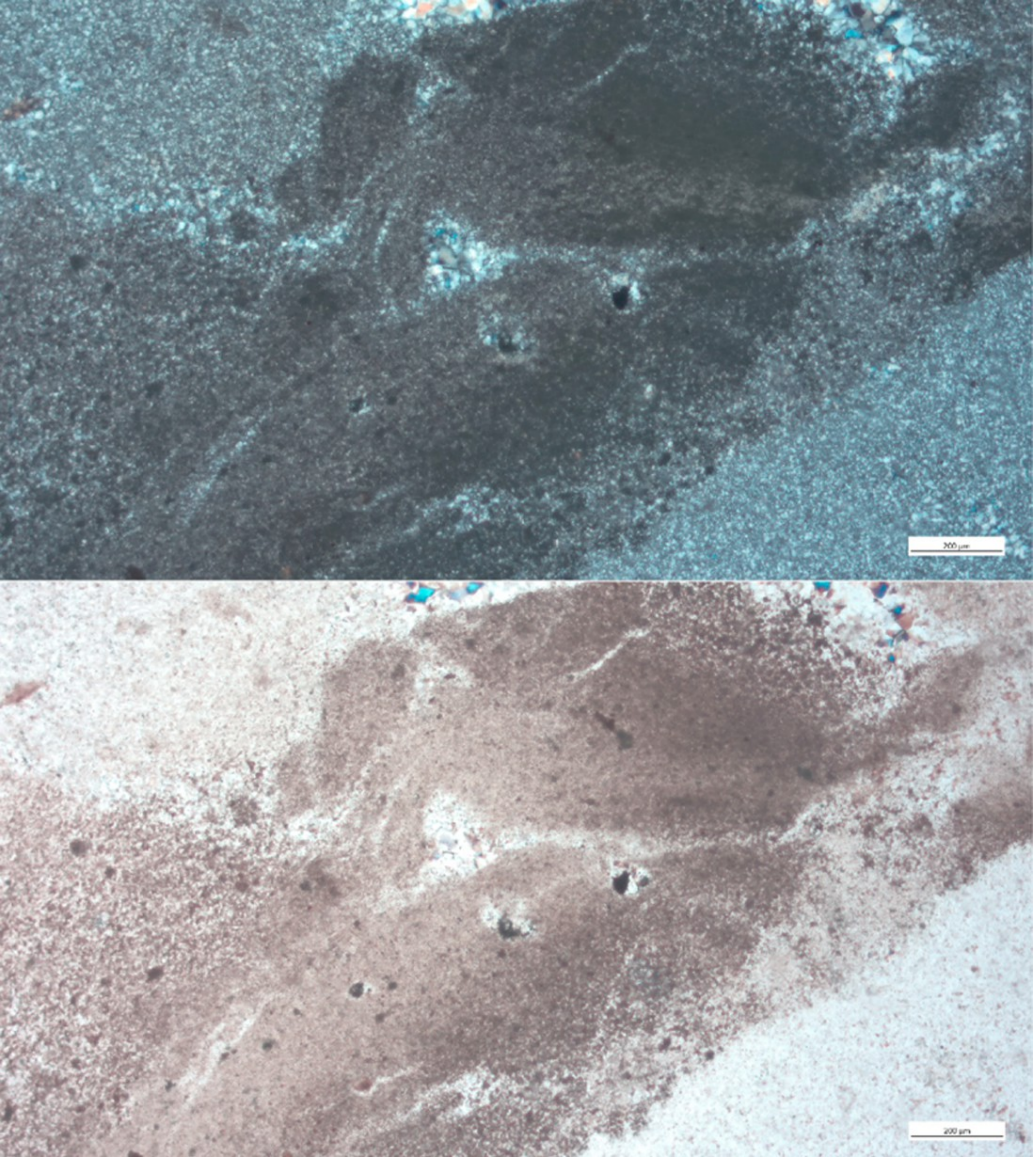
The brecciated texture of the Mishash flint, in cross polars (above) and in plain polars (below).

**Fig 19:**
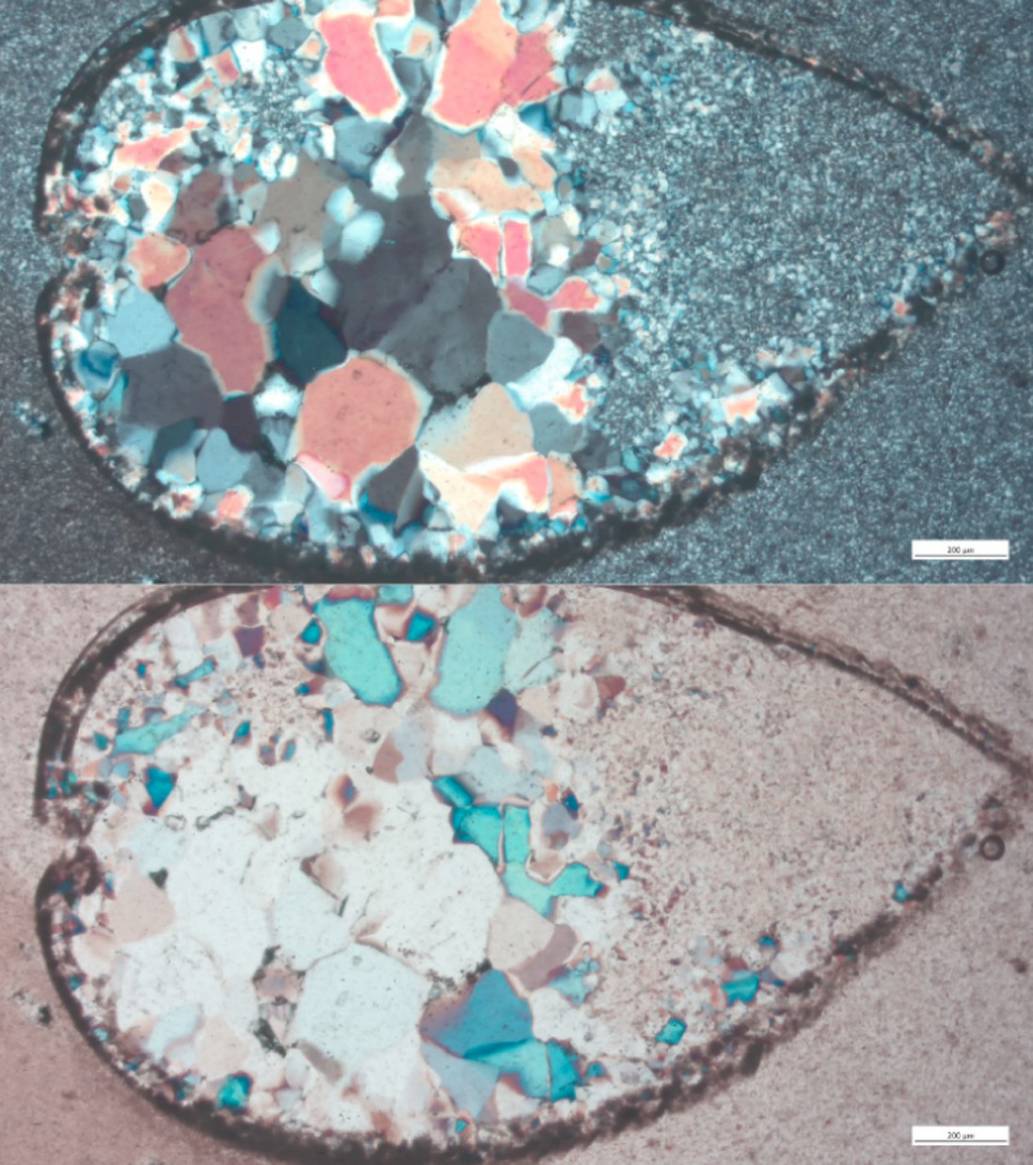
An oval ostracod observed in one of the Mishash flint samples, in cross polars (above) and in plain polars (below), infilled with large grains of quartz.

Thin sections of Campanian Mishash samples show a clear brecciated texture, which is often considered a marker of Campanian Mishash flint ([36] but see [37]), with high concentrations of iron (Fig 18). Fossils observed in the Campanian samples include shell fragments, coiled foraminifera, possible gastropods, and ostracods (Fig 19), though no *bulimina foraminifera*, which are considered a marker of Campanian Mishash flint [33], were observed.

**Fig 20:**
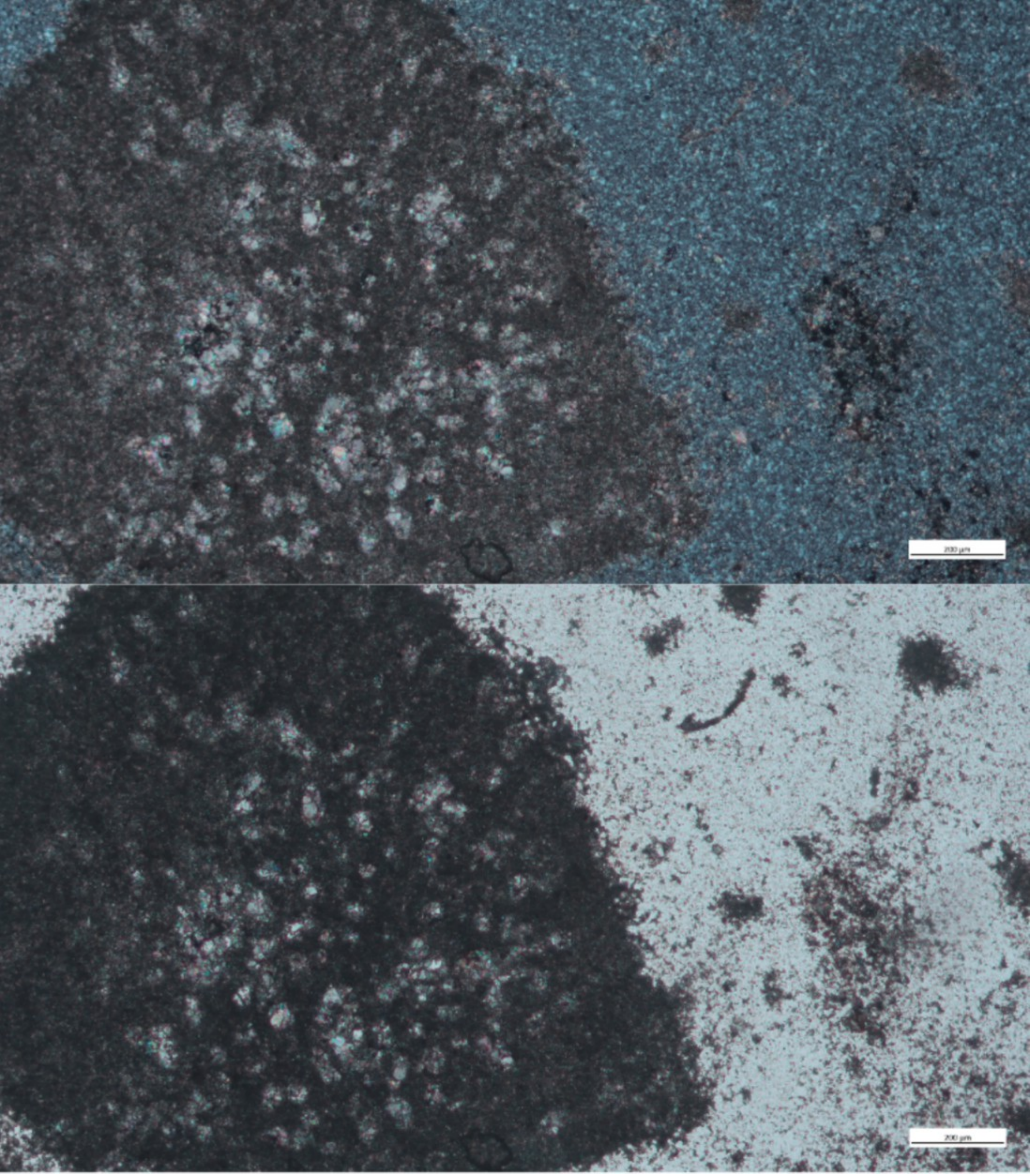
A concentration of foraminifera in a Turonian Bi’na sample, in cross polars (above) and in plain polars (below).

The Turonian Bi’na flint analyzed is fine-grained, with some calcite content. Several fossils were observed, including shell fragments, ostracods, and foraminifera, which were not identified to a type level (Fig 20).

Combined with the macroscopic resemblance between the geologic flint and the archaeological analyzed here, we consider the classification of the archaeological artifacts to Turonian and Campanian geologic origin as secure.

**Fig 21:**
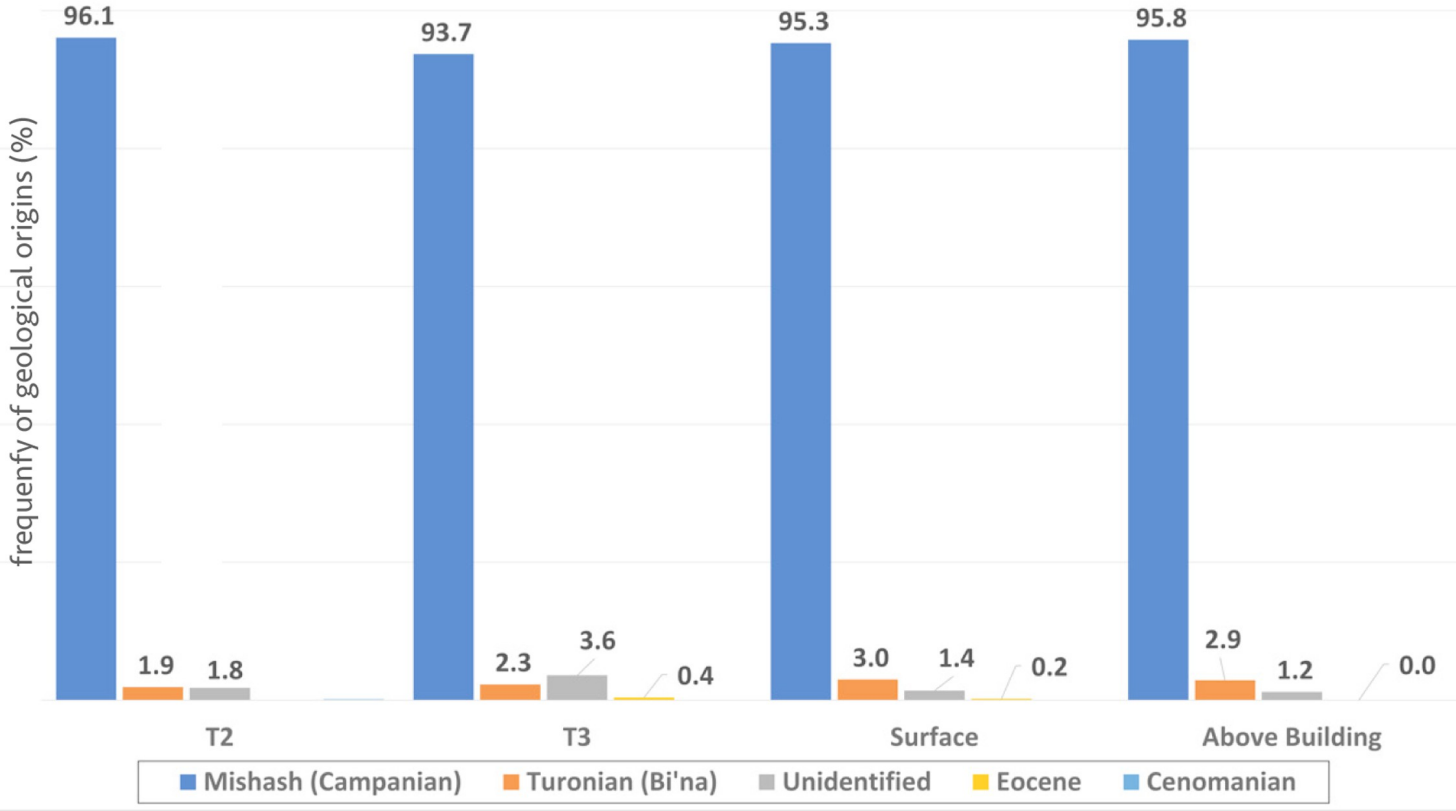
Frequencies of the geologic flint raw material by assemblage.

The flint collected and excavated from the slopes of Kaizer Hill numbers 21,391 flint pieces weighting in total 61.16 kg. Of the four assemblages, the ‘Surface collection’ is the largest (n = 9,520) and the heaviest (27.73 kg) (Table 3 and Fig 21). While located directly on top of outcrops of the Turonian Bi’na Fm., all four assemblages are dominated by Campanian flint of the Mishash Fm., in proportions ranging between 93.7% (T3 excavation) and 96.1% (T2 excavation). This Campanian Mishash flint most likely originated from the Campanian outcrops located on top of the hill. The Turonian flint of the Bi’na Fm. appears in surprisingly low percentages, ranging between 3.6% (T3 excavation) and 1.9% (T2 excavation). Possibly Cenomanian and Eocene pieces appear in single numbers. These flint pieces could have been brought to the site during occasional trips in the surrounding of the site, rather than through regular, systematic procurement activities.

**Table 3:** The lithic assemblages analyzed by geologic origins and weight.

Assemblage	N of artifacts	Weight (gr)	% N out of total assemblage	% weight out of total assemblages
**Surface (T0-3)**				
Campanian (Mishash)	9034	26429	94.9	95.3
Turonian (Bi’na)	178	829	1.9	3.0
Unidentified	304	398	3.2	1.4
Eocene	2	68	0.0	0.2
Cenomanian	2	9	0.0	0.0
** Sub Total**	**9520**	**27733**	**100.0**	**100.0**
**T2 (excavation)**				
Campanian (Mishash)	5985	10352	96.1	96.1
Turonian (Bi’na)	105	208	1.7	1.9
Unidentified	133	193	2.1	1.8
Cenomanian	3	14	0.0	0.1
** Sub Total**	**6226**	**10767**	**100.0**	**100.0**
**T3 (excavation)**				
Campanian (Mishash)	2150	7570	92.4	93.7
Unidentified	131	292	5.6	3.6
Turonian (Bi’na)	40	185	1.7	2.3
Eocene	6	30	0.3	0.4
** Sub Total**	**2327**	**8077**	**100.0**	**100.0**
**Surface (above building)**				
Campanian (Mishash)	3231	13962	97.4	95.8
Turonian (Bi’na)	34	427	1.0	2.9
Unidentified	50	181	1.5	1.2
Cenomanian	1	5	0.0	0.0
Eocene	2	4	0.1	0.0
** Sub Total**	**3318**	**14579**	**100.0**	**100.0**
** Total**	**21391**	**61156**	**100.0**	**100.0**

Artifacts made of Turonian Bi’na flint generally tend to be larger pieces (an average of 4.62 grams per piece), compared to artifacts made of Campanian Mishash flint (an average of 2.86 grams per piece). The ten possibly Eocene pieces are relatively large, with an average of 10.2 grams per piece. The six possibly Cenomanian pieces average 4.67 grams per piece.

The strong dominance of Mishash flint appears also on a type of tool/blank level. Of the tools (excluding bifaces), 99.3% are made of Campanian Mishash flint. Only three tools (a burin, a retouched blade, and a ‘varia’ artifact) were made of Turonian Bi’na flint. This may imply the negligible use of Turonian flint for the production of formal tools, including bifaces. Indeed, out of 107 bifaces, 106 are made of Campanian Mishash flint (99.1%), and one is made of possibly Eocene flint (0.9%). Among the cores, 271 out of 275 are made of Mishash flint (98.5%). Among the blades and bladelets, Campanian flint constitutes 95.3%. In no category was Campanian Mishash flint found in proportions lower than 90%.

## The chipped stone assemblages

The following is a techno-typological description of the chipped stone inventory comprising artifacts of a detailed stratigraphic context, with an emphasis on the bifaces and their production.

### Debitage and debris

As already stated above, most of the chipped stone artifacts, in all four assemblages, are production waste.

‘Debitage’ (Table 4) comprises the common types, mostly representing technological waste rather than blanks for further modification. Though there is quite a difference in the total number of debitage items between the four assemblages, ranging from 2218 (T0-3 surface) to 194 (T3 excavation), the percentages of the debitage categories are quite similar. Clearly, flakes predominate (65.4% of the total, the range being 59.1%-69.2%). The ‘Primary Elements’ (> than a 3^rd^ of the surface cortical) are almost exclusively flakes and the same is valid for the ‘Core trimming Elements’. Blades and bladelets put together comprise but 3.5% of the total (ranging from 2.2% to 7.2%), though there is a possibility that there were more bladelets since the ‘Chips’ in the Debris category most probably comprise fragments of broken bladelets.

**Table 4:** Debitage and debris categories–Kaizer Hill Terraces.

Kaizer Terraces	Surface (T0-3)		T2 (excavation)		T3 (excavation)		Surface (above building)		TOTAL	
**DEBITAGE**	N	%	N	%	N	%	N	%	N	%
Primary Elements	288	13.0	156	12.3	26	13.4	245	13.1	715	12.9
Flakes	1311	59.1	764	60.2	97	50.0	1055	56.5	3227	58.1
Thinning Flakes	211	9.5	121	9.5	24	12.4	49	2.6	405	7.3
Blades	40	1.8	17	1.3	9	4.6	22	1.2	88	1.6
Bladelets	44	2.0	38	3.0	5	2.6	18	1.0	105	1.9
Core Trimming Elements	285	12.8	144	11.3	31	16.0	434	23.2	894	16.1
Bifacial Spalls	35	1.6	10	0.8	1	0.5	29	1.6	75	1.4
Burin Spalls	4	0.2	20	1.6	1	0.5	16	0.9	41	0.7
**TOTAL**	**2218**	100.0	**1270**	100.0	**194**	100.0	**1868**	100.0	5550	100.0
**DEBRIS**										
Chunks	26		8		4		13		51	
Chips	5805		4535		523		1522		12385	
**TOTAL**	**5831**		**4543**		**527**		**1535**		**12436**	

The waste pertaining to shaping and modification of bifaces will be discussed in detail below. Suffice it to say that they constitute on the average a small percentage of the debitage (‘Thinning Flakes’– 7.3% and ‘Bifacial Spalls’– 1.4%).

There is only one other debitage category observed in the lithic assemblages, namely the ‘Burin Spall’ comprising 0.7% of the total (range: 0.2%-1.6%).

The ‘Debris’ comprises the ‘Chunks’ and ‘Chips’ categories (Table 4) and the number of the latter is rather impressive when one considers that the majority of the chips derives from surface collections. The ratio of debitage-per-core (average: 20-per-1) as well as the absolute percentages of ‘Primary Elements’ and ‘Core Trimming Elements’ indicate that chipped stone production might have taken place on-site.

### Cores (N = 275; for details see Tables 5 and 6)

**Table 5:** Core types–Kaizer Hill Terraces.

Core Type	Surface (T0-3)		T2 (excavation)		T3 (excavation)		Surface (above building)		TOTAL	
	N	%	N	%	N	%	N	%	N	%
Nodule	6	4.5	4	8.7	0	0	5	5.7	15	5.5
1 st.pl. on flake	8	6.0	1	2.2	0	0	3	3.4	12	4.4
1 st.pl. on chunk	19	14.3	7	15.2	1	12.5	12	13.6	39	14.2
1 st.pl. indet.	3	2.3	1	2.2	0	0	5	5.7	9	3.3
Pyramidal—like	12	9.0	3	6.5	0	0	10	11.4	25	6.7
2 st.pl. opposed	11	8.3	3	6.5	1	12.5	6	6.8	21	7.6
2 st.pl. observe	4	3.0	5	10.9	0	0	3	3.4	12	4.4
2 st.pl. at 90°	7	5.3	1	2.2	0	0	3	3.4	11	4.0
3 st.pl.	1	0.8	3	6.5	0	0	0	0	4	1.5
Varia	5	3.8	2	4.4	1	12.5	7	8.0	15	5.5
Exhausted	5	3.8	4	8.7	0	0	5	5.7	14	5.1
Amorphic/Burnt	27	20.3	8	17.4	3	37.5	15	17.1	53	19.3
Broken	19	14.3	3	6.5	0	0	14	15.9	36	13.1
core/hammerstone	6	4.5	1	2.2	2	25.0	0	0	9	3.3
**TOTAL**	**133**		**46**		**8**		**88**		**275**	

**Table 6:** Core categories–Kaizer Hill Terraces.

Core Category	Surface (T0-3)		T2 (excavation)		T3 (excavation)		Surface (above building)		TOTAL	
	N	%	N	%	N	%	N	%	N	%
Nodule	6	4.5	4	8.7	0	0	5	5.7	15	5.5
1 striking platform	42	31.6	12	26.1	1	12.5	30	34.1	85	30.9
2 striking platforms	22	16.5	9	19.6	1	12.5	12	13.6	44	16.0
3 striking platforms	1	0.8	3	6.5	0	0	0	0	4	5.3
Varia/Exhausted	10	7.5	6	13.0	1	12.5	12	13.6	29	10.6
Amorphic/Burnt/Broken	46	34.6	11	21.7	3	37.5	29	33.0	89	32.4
core/hammerstone	6	4.5	1	16.7	2	25.0	0	0	9	3.4
**TOTAL**	**133**		**46**		**8**		**88**		**275**	

All in all, the frequencies of the core types in all assemblages studied herein are quite similar. Still, the absolute number of cores varies. One, that of T3-excavation, stands out with just 8 cores as compared to the 133 cores from T0-3 surface, 88 cores in above building, to 46 from T2-excavation.

The most frequent core type belongs to the one striking platform category (N = 85), nearly double the number of the two striking platforms category (N = 44). The rest of the cores are mainly either broken or burnt items (N = 89), comprising ca. a third of the whole core amount. One may also note that some of the cores, besides those actually defined as cores/hammerstones, portray signs of battering (N = 8, in single numbers from all four assemblages).

Besides the cortical nodules (‘tested’ chunks with 3 ≥ flaking removals) (N = 15; 5.5%), cortex is observed on a small number of items in the various categories (except the burnt and broken specimens): three cores with some cortex from T0-3 surface, two items from T2-excavation, and one item from T3-excavation, the exception being the cores of the above building assemblage with 15 items retaining some cortex on their surface–the coverage is much less than a third of the surface. These are mostly cores with one striking platform, on a chunk.

Apart from the regular types of the ‘one striking platform’ category–on flake and on chunk–it is of interest to note the type (comprising ca. 10% of the category) with equally intense flaking on both the flaking surface and the striking platform. Most of the cores belonging to the ‘two striking platforms’ category are of the opposed variety (N = 33) with those of the ‘at 90°’ type comprising only 10 items. Cores with clearly discernable ‘three striking platforms’ are rare (N = 4), though perhaps some of the exhausted items, which are as a rule quite small and amorphous, may have had originally three striking platforms–a case in point is observed among the exhausted cores in the assemblage T0-3 surface. The ‘varia’ category comprises items which do not fit any of the other categories, e.g., two ‘varia’ cores from T0-3 surface are two elongated chunks, with a polygonal section, similar to roughouts of bifaces but not diagnostic enough to be defined as such.

Hammerstones: Besides the 9 cores/hammerstones (Table 6), there are a couple of other items that were most probably hammerstones. Three are from T0-3 surface–one is actually a broken hammerstone (a chunk), another is a heavily weathered item of limestone with fossils, while the third is a cherty limestone pick that was used as a hammerstone. Two other items derive from above building’–those are two big, though broken, hammerstones of the same raw material, cherty limestone.

Tools: Overall, most of the tools show little investment and can be considered as ad-hoc tools, a feature quite commonly observed in PPNA assemblages in general (e.g., [38]) and those deriving from quarries in particular (e.g., [39]). We can add to these 83 items (excluded from the tool counts) bearing signs of use-wear.

Except for bifaces which comprise ca. 12% of the total tools (ranging from 6.3% to 18.5%; see Table 7), no other typical PPNA tools were recovered, i.e., arrowheads or/sickle blades. The only sickle blade found is a broken item, actually a multiple tool recovered in T3 excavation. It is a medial fragment, retouched with a spike shaped on the opposed lateral.

**Table 7:** Tool categories–Kaizer Hill Terraces.

TOOL CATEGORY	Surface (T0-3)		T2 (excavation)		T3 (excavation)		Surface (above building)		TOTAL	
	N	%	N	%	N	%	N	%	N	%
Endscrapers	24	6.9	2	1.4	6	9.2	28	8.9	60	6.9
Burins	25	7.2	9	6.3	4	6.2	12	3.8	50	5.7
Borers & Awls	111	32.1	60	41.7	19	29.2	114	36.1	304	34.9
Backed Pieces	7	2.0	1	0.7	0	0.0	3	0.9	11	1.3
Truncations	11	3.2	1	0.7	0	0.0	2	0.6	14	1.6
Notches & Denticulates	29	8.4	22	15.3	3	4.6	35	11.1	89	10.2
Retouched Pieces	26	7.5	17	11.8	6	9.2	27	8.5	76	8.7
Multiple Tools	29	8.4	7	4.9	6	9.2	30	9.5	72	8.3
Microliths, non-Geo.	0	0.0	2	1.4	0	0.0	2	0.6	4	0.5
Bifaces	52	15.0	9	6.3	12	18.5	34	10.8	107	12.3
Varia	32	9.2	14	9.7	9	13.8	29	9.2	84	9.6
**TOTAL**	**346**	100.0	**144**	100.0	**65**	100.0	**316**	100.0	**871**	100.0

The dominant tool blank are flakes (see S1 Text for a detailed account) which also fits-in with their proliferation among the debitage items. Some 8% of the tools are modified on double patinated blanks, ca. 11% of the tools are on primary elements, ca. 5% on biface thinning flakes and ca. 1% on bifacial spalls (Table 8).

**Table 8:** Technological observations and double patina of tools—Kaizer Hill Terraces.

TOOLS	Surface (T0-3) (N = 346)		T2 (excavation) (N = 144)		T3 (excavation) (N = 65)		Surface (above building) (N = 316)		TOTAL	
	N	%	N	%	N	%	N	%	N	%
Double Patina	30	8.7	9	6.3	0	0	32	10.1	71	
On Primary Elements	36	10.4	16	11.1	4	6.2	36	11.4	92	
On Thinning Flakes	7	2.0	18	12.5	6	9.2	16	5.1	47	
On Bifacial Spalls	4	1.6	0	0	0	0	4	1.3	8	
**Use-Wear Items**	**46**		**17**		**6**		**83**			

**Fig 22:**
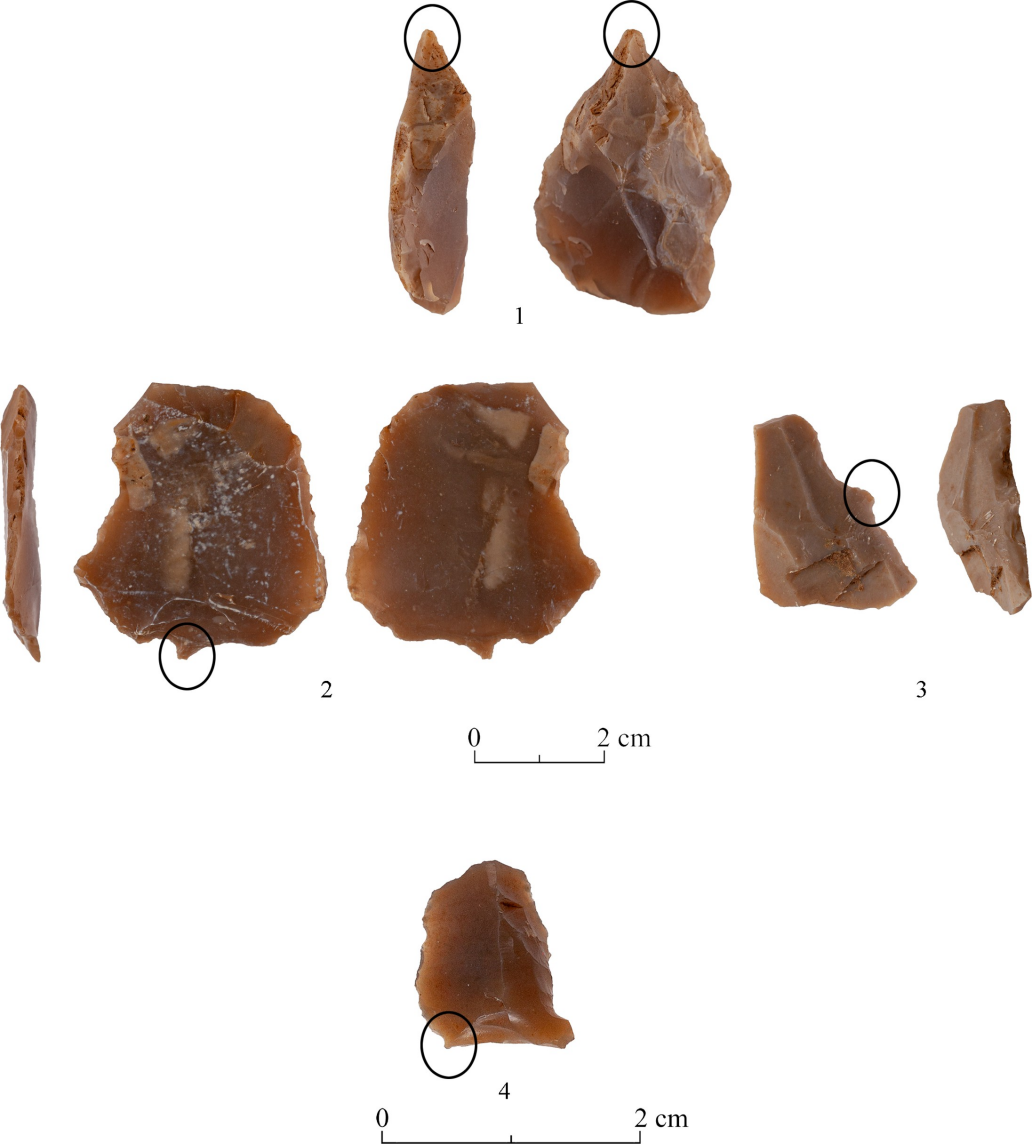
Borers and awls: 1–4.

As known for other PPNA sites [27], the main tool category (representing 34.9% of the studied tools) is the ‘Borers & Awls’. The majority are multiple tools comprising every possible combination of borers-awls-spikes-becs and heavy-duty borers (ca.41%). (Tables 7 and 9 and Figs 22:1–4 and 23:1–5) Actually, the numbers of the ‘borer’ varieties are even greater since some of the items incorporated in the ‘Multiple Tools’ category are also of the ‘borer’ family (T0-3 surface = 22; T2 excavation = 6; T3 excavation = 1; Above Building = 23), thus the actual count is 356 specimens (ca. 41%). Still, this number can be even higher if we add here some items included in the category of ‘Notches & Denticulates” which most probably represent broken borer varieties (see S1 Text for details).

**Fig 23:**
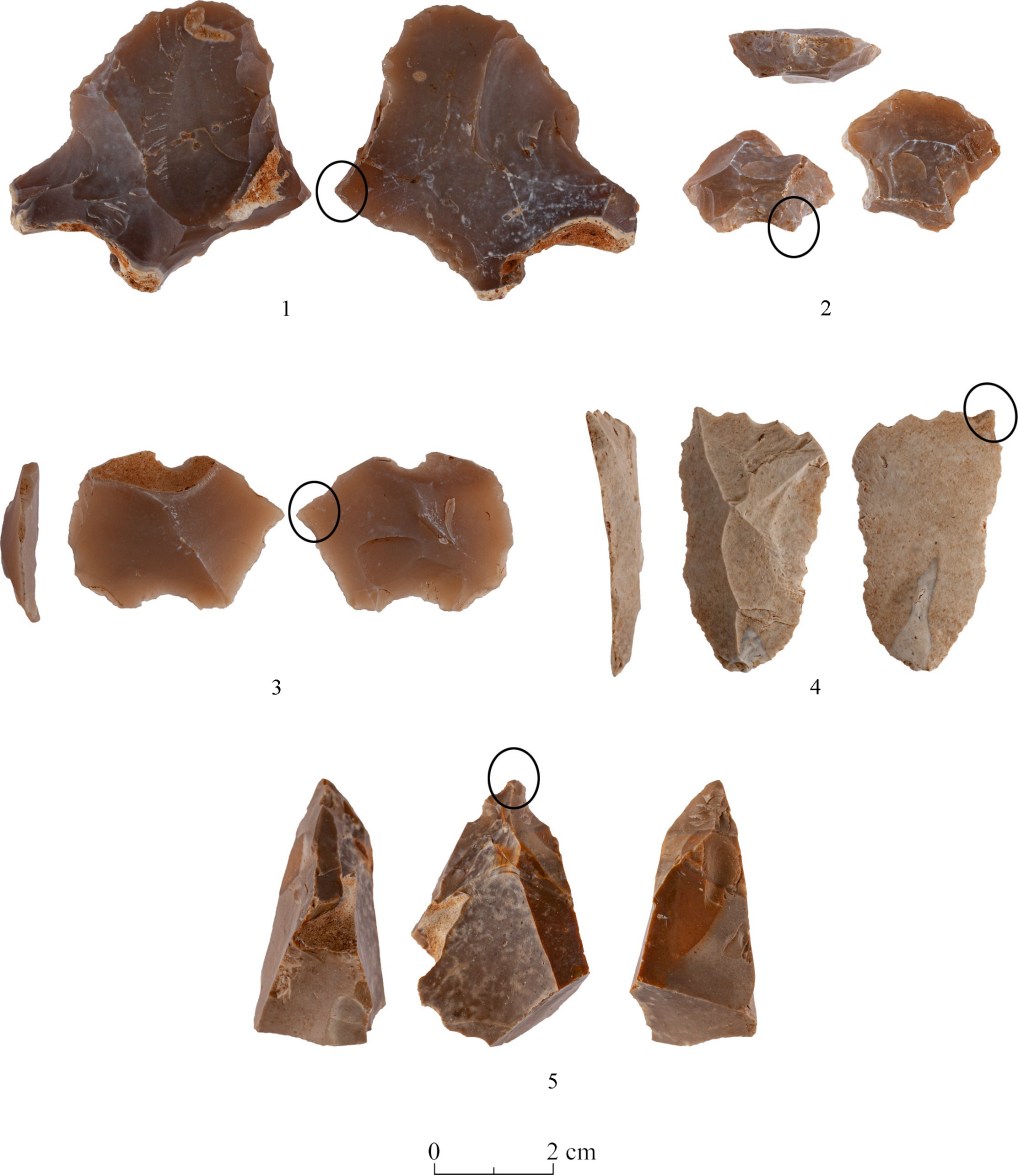
Borers and awls: 1–5.

**Table 9:** ‘Borers and Awls’ tool types–Kaizer Hill Terraces.

Tool Type	Surface (T0-3)		T2 (excavation)		T3 (excavation)		Surface (above building)		TOTAL	
	N	%	N	%	N	%	N	%	N	%
spike	19	17.1	20	33.3	2	10.5	15	13.2	56	18.4
awl	9	8.1	8	13.3	3	15.8	9	7.9	29	9.5
borer	4	3.6	12	20.0	2	10.5	0	0.0	18	5.9
bec	29	26.1	3	5.0	4	21.1	23	20.2	59	19.4
double/multiple	43	38.7	17	28.3	8	42.1	58	50.9	126	41.4
varia	7	6.3	0	0.0	0	0.0	9	7.9	16	5.3
	**111**	**100.0**	**60**	**100.0**	**19**	**100.0**	**114**	**100.0**	**304**	**100.0**

**Fig 24:**
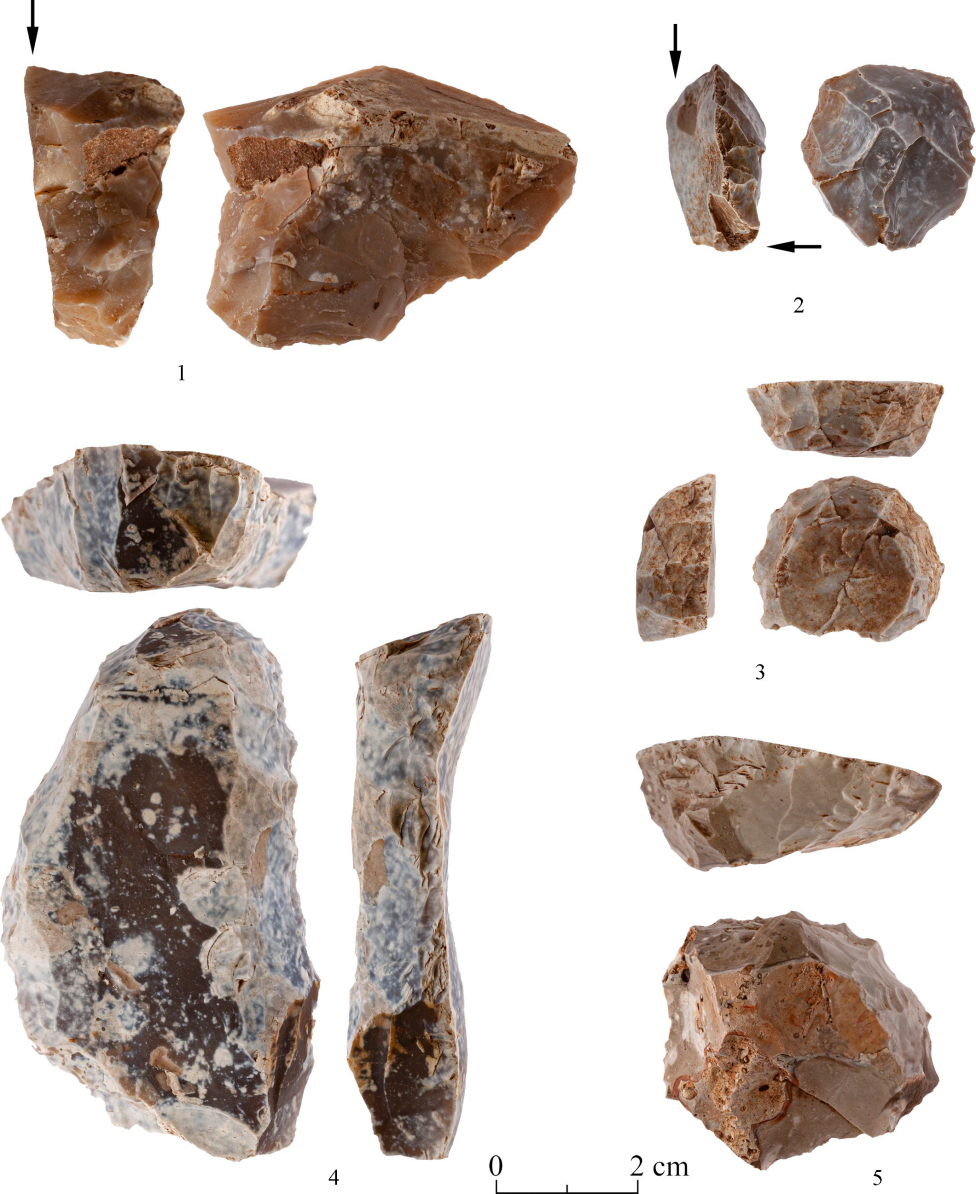
Burins: 1–2 and Endscrapers: 3–5.

‘Notches & Denticulates’ comprise the next-in-line largest category (if we exclude the bifaces), comprising 10.2% of the total tools. All the other categories account for less than 10% of the tools, with ‘Varia’ comprising 9.6%, while ‘Retouched Pieces’ and ‘Multiple Tools’ comprise each ca. 8%. ‘Endscrapers’ (Fig 24: 3–5) are a bit more numerous than ‘Burins’ (Fig 24:1, 2) (6.9% and 5.7% respectively) and all the rest represent less than 2% of the tools. Of interest to note the scarcity of bladelet (-microlithic) tools– 0.5% -, which fits well with bladelets representing the smallest percentage of potential tool blanks in the Debitage (Table 4).

BIFACES: Bifaces are the most dominant and culturally indicative tool type of both the Kaizer Hilltop [11] and the Terraces assemblages. In this study we used the entire biface sample to characterize the raw material and the shape of the bifaces, while the detailed aspects of their technology and that of their waste products are presented here only for the finds discovered on the Terraces.

All in all, 155 bifaces were recovered from the site of Kaizer– 38 from the Hilltop, 107 from the Terraces and 10 are of unclear context and nearly all of them (99.4%) were made on Mishash flint. As seen in Table 10 there are 26 complete bifaces from the Terraces, the rest are biface fragments, ‘varia’ and preforms. Most of those were found on the surface, with only a minority comprising part of the excavated assemblages. As in many other PPNA lithic assemblages [24], the broken tools form a major component of the biface assemblage (63.6%). Table 10 provides details concerning the breakage types (proximal, distal or medial) and their counts.

**Table 10:** Biface distribution in Kaizer Hill Terraces assemblages.

	Terraces					
Axes	Surface		Excavation		Total	
	N	%	N	%	N	%
Complete	21	24.42	5	23.81	26	24.30
Distal end	20	23.26	5	23.81	25	23.36
Proximal end	30	34.88	2	9.52	32	29.91
Medial part	8	9.30	3	14.29	11	10.28
Varia	4	4.65	5	23.81	9	8.41
Preform	3	3.49	1	4.76	4	3.74
**TOTAL**	**86**	**100.00**	**21**	**100.00**	**107**	100.00

**Fig 25:**
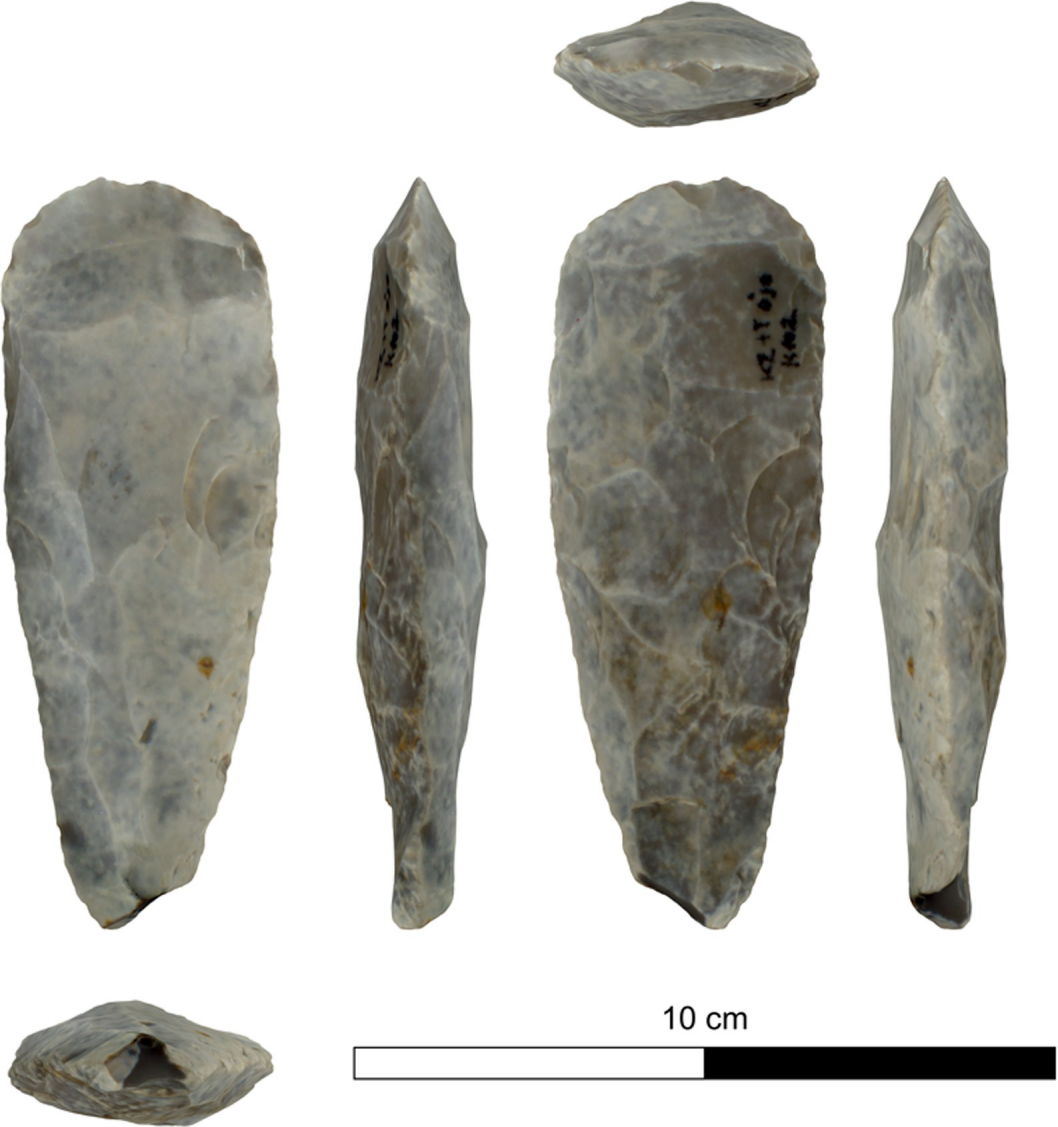
Finely made biface (a ‘prestige item’) (#142).

**Fig 26:**
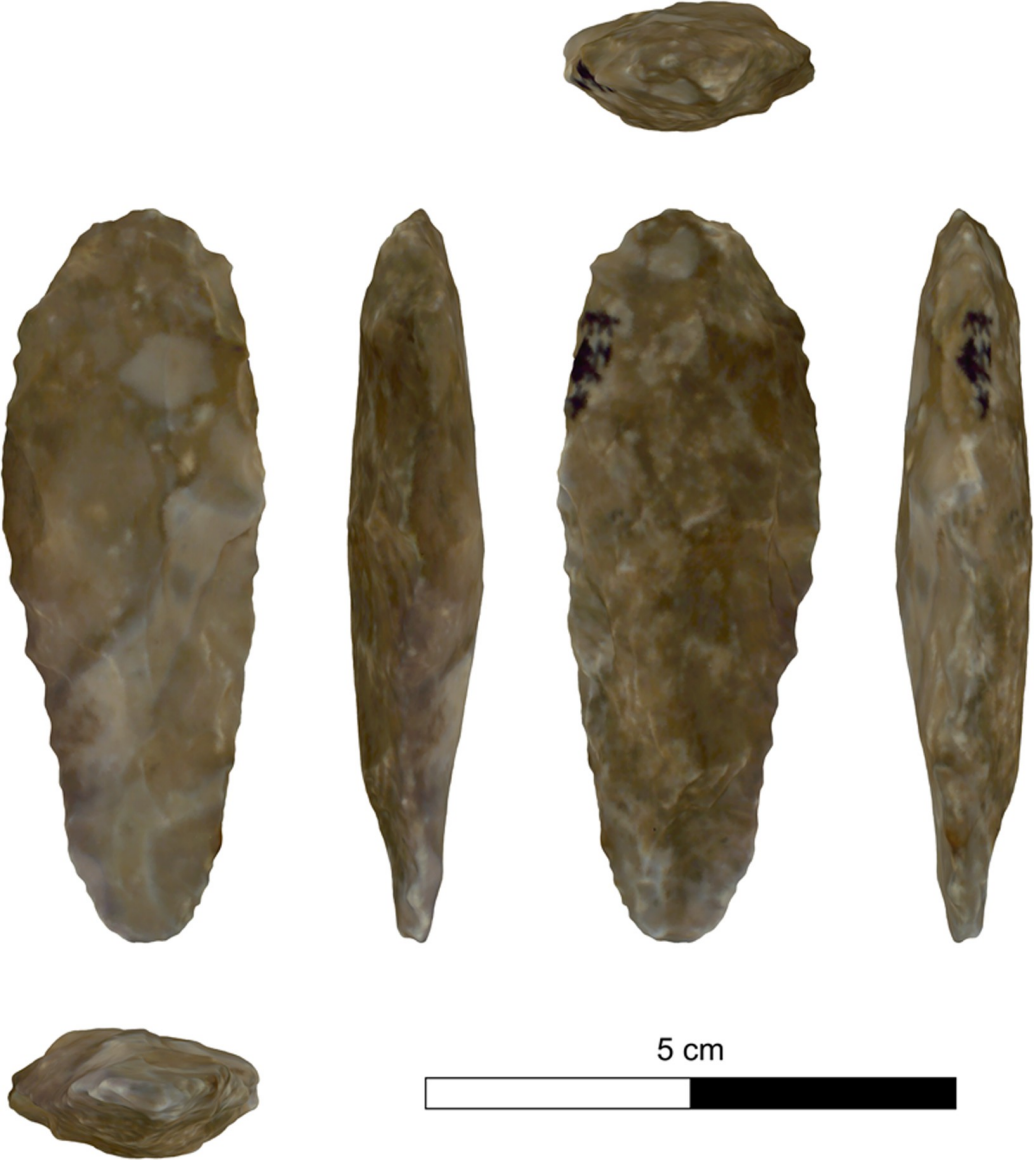
Finely made biface (a ‘prestige item’) (#020).

**Fig 27:**
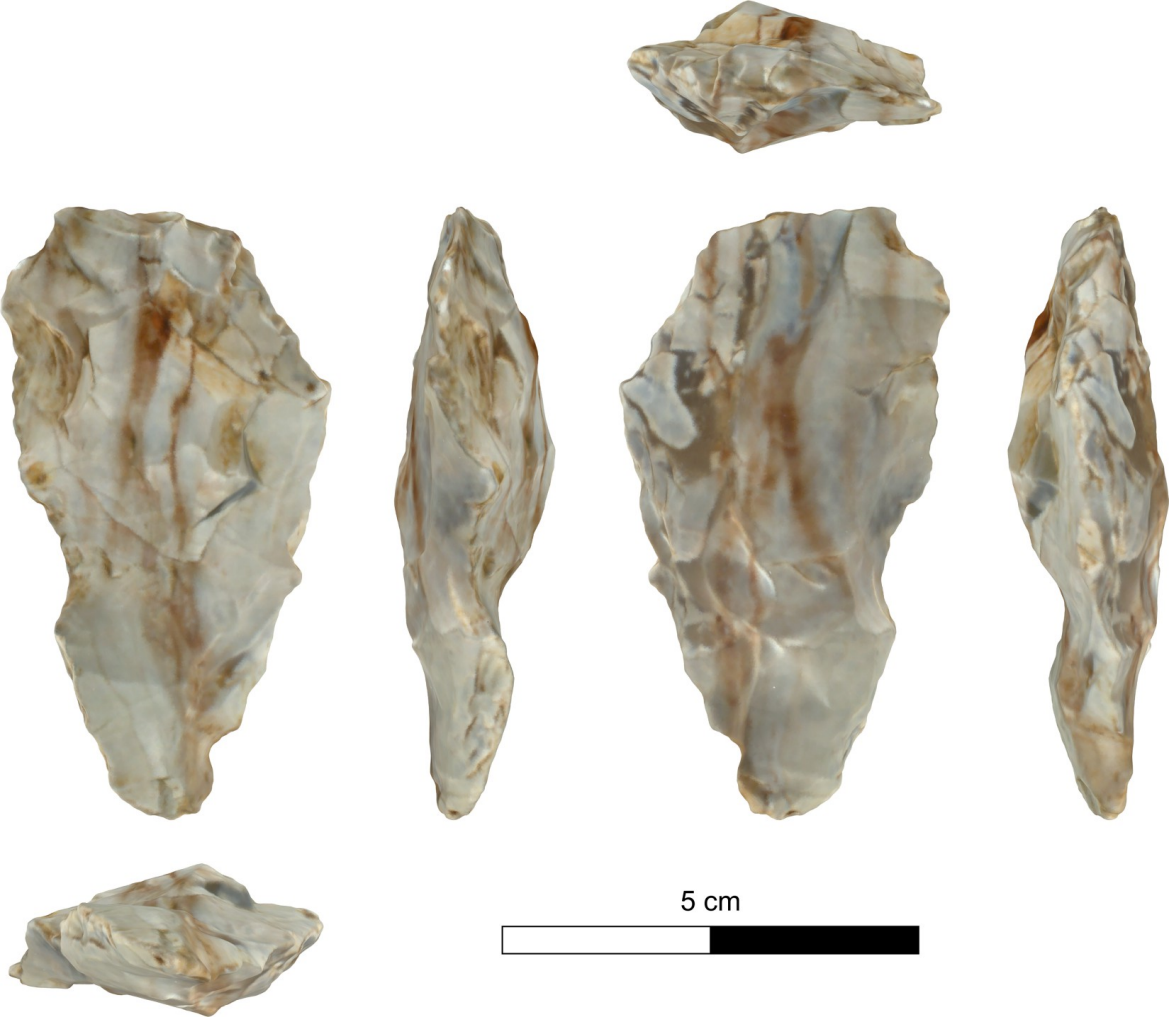
A coarsely made biface (#025).

The bifacial tools comprise both, well-made bifaces considered to be ‘prestige items’ (Figs 25 and 26), and lower quality, ‘sloppily’ produced tools, lacking finesse (Fig 27). While it seems that the former were made for distribution beyond the boundaries of the quarry-cum-production Kaizer site (such ‘prestige’ tools are a typical find in many PPN sites, (e.g., [10, 24, 27]), we hypothesize that the latter were produced as quarrying-tools to be used in the local quarrying activities. These ‘mundane’ bifaces are generally small-sized, usually rough, unstandardized, at times asymmetrical, and are characterized by a relatively deeper scar pattern when compared to the ‘prestigious’ tools. There are also a small number of artifacts that are classified as ‘preforms’, i.e., bifaces in their very initial stage of production, lacking the typical morphology and shaping scar-pattern of biface tools. Of interest to note that typologically all Kaizer bifaces belong to the category of axes and its varieties.

**Fig 28:**
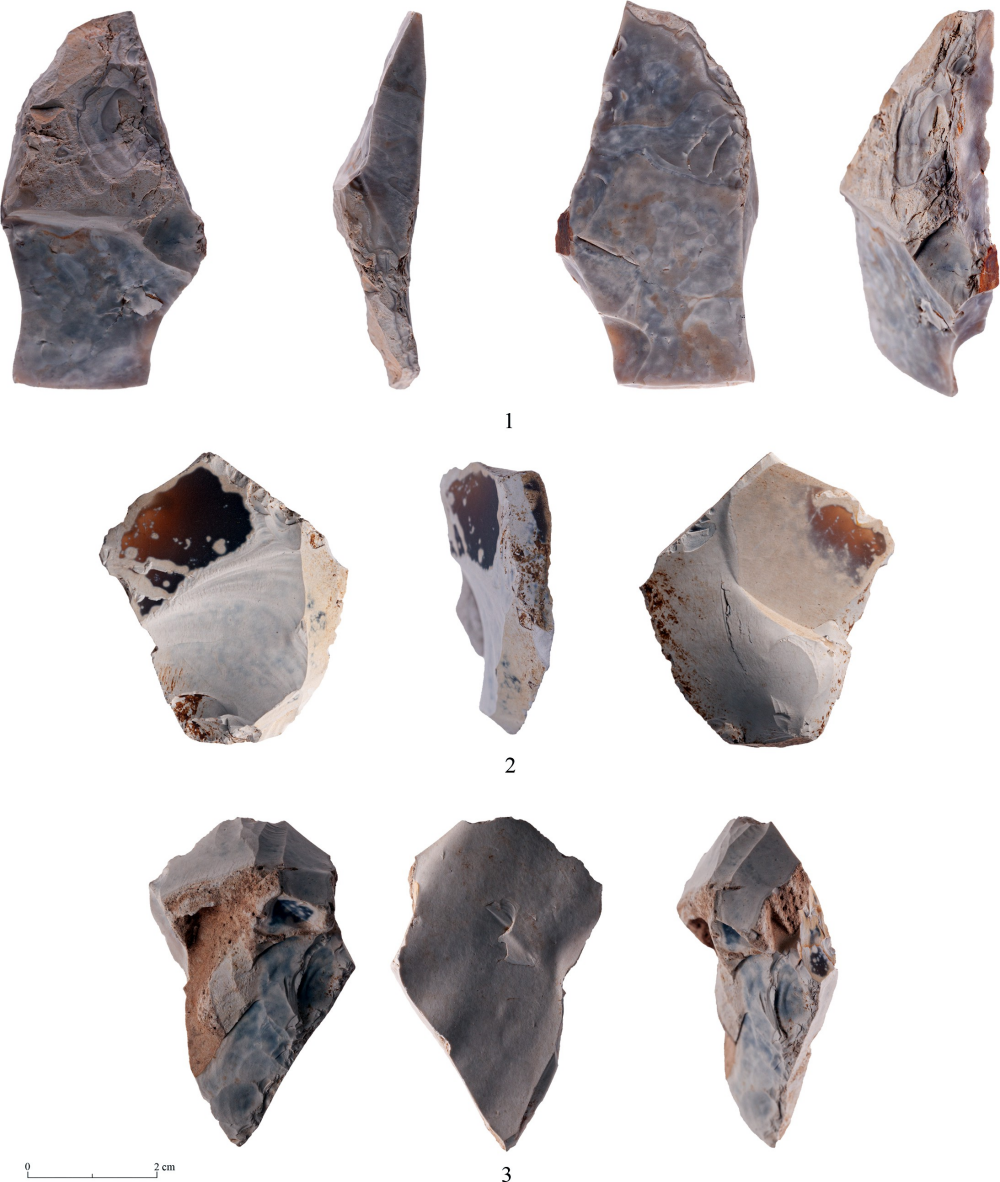
Waste products typical of the early stages of biface production.

There are several types of debitage associated with the production and modification of bifaces. In the following we shall deal mostly with two particular categories, yet it is important to state that there are also other debitage products which indicate biface production. Among these are artifacts typical of the beginning of the reduction process, reflecting the attempt to form an appropriate striking platform for removal of various surface ‘obstacles’ (Fig 28).

**Fig 29:**
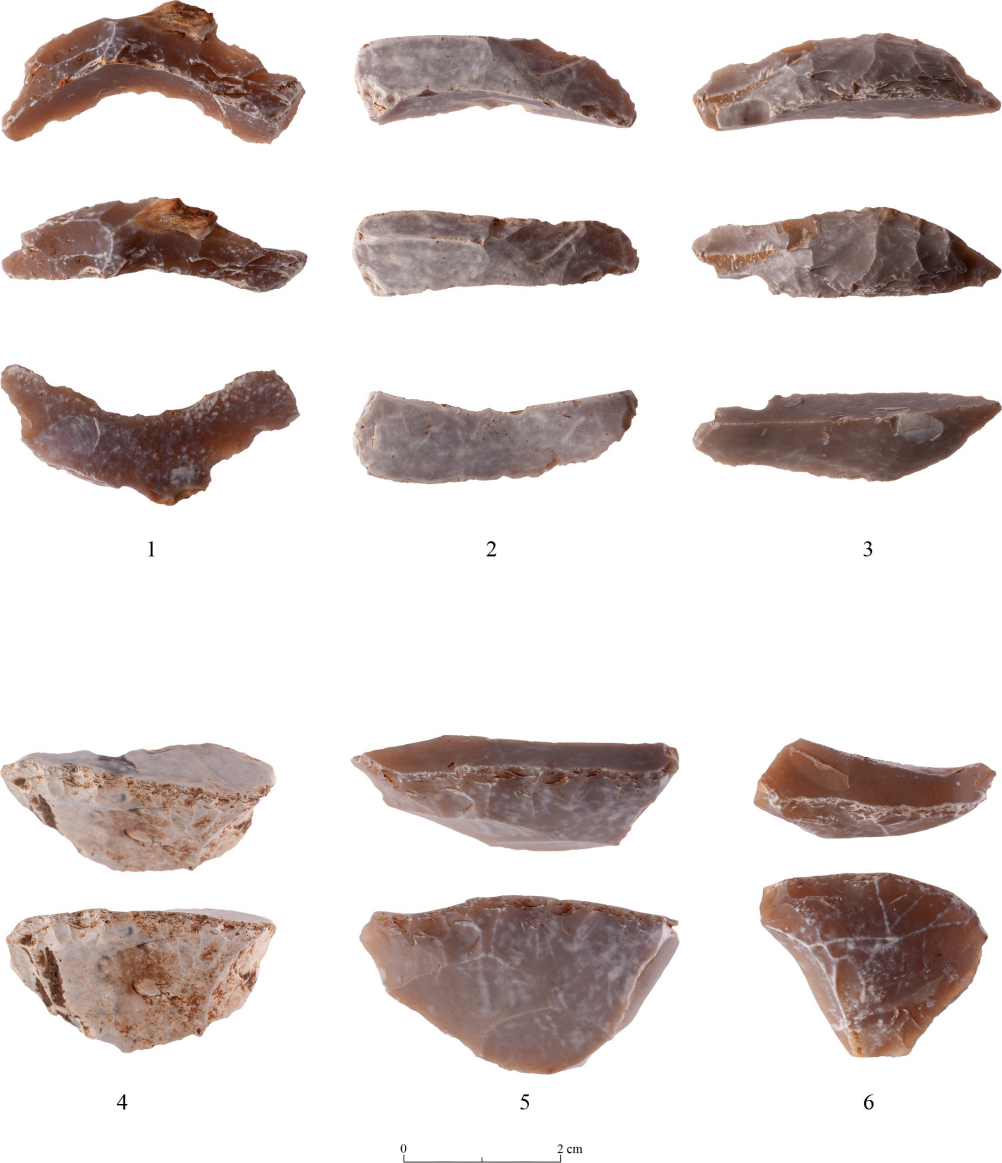
Transversal spalls.

The two debitage categories detailed herewith are the ‘thinning flakes’ which are small, thin flat flakes reflecting the final stages of biface shaping and thinning [40]). The second category are artifacts resembling morphologically *tranchet* spalls, yet they are part of the process of biface production (Figs 29:1 and 30).

**Fig 30:**
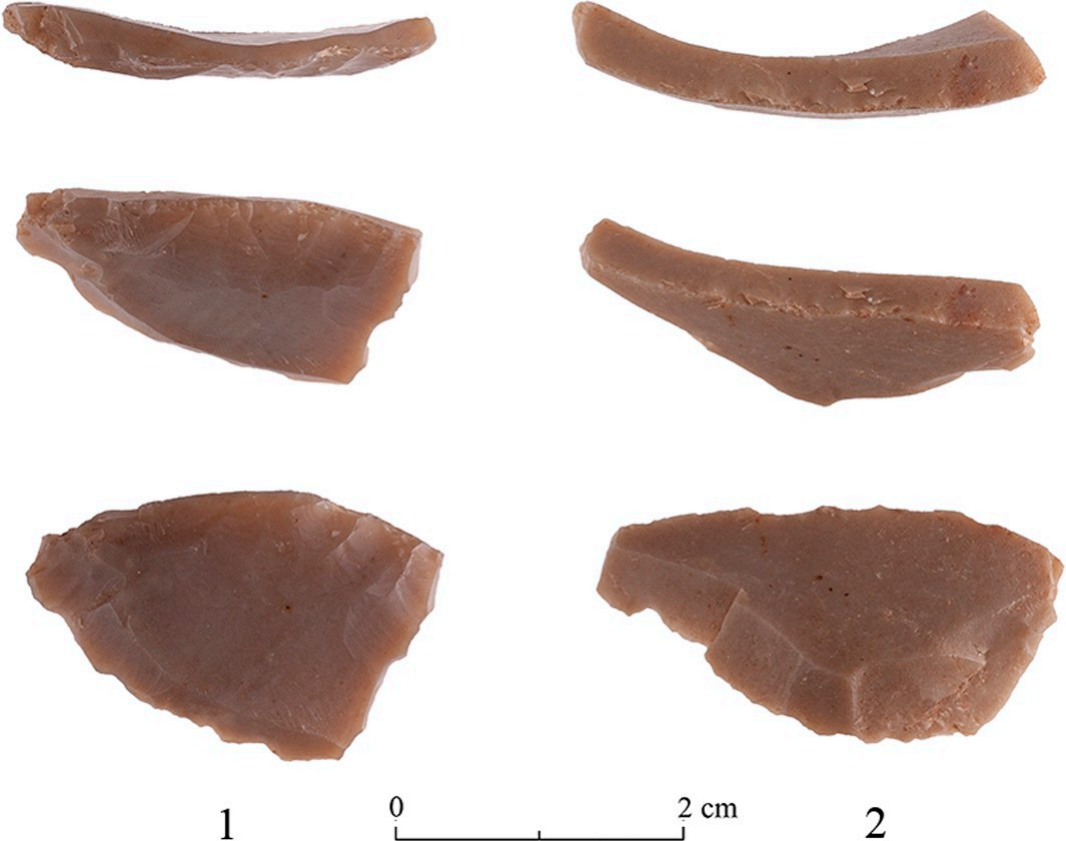
Transversal spalls.

The original blank type of the bifaces is of great importance when attempting to reconstruct the reduction sequence. To begin with, it is difficult to identify the blanks because of the bifacial intensive flaking which masks the original nature of the blank, especially at the last stage of “thinning and finishing”.

Our analysis revealed that the Terraces flint assemblages lack large sized artifacts (with the exception of very large hammerstones which are a particular component of the quarrying toolkit; see Table 3). In addition, the small frequencies of cortical elements (primary flakes– 12.9%) in the debitage as well as the presence of several bifaces with some cortex (8.4%) indicate that the bifaces were rather small and at least ca. 18% of them were modified on flakes. As a large component of the bifaces are broken items (Table 10) it was not possible to provide an exact account of how many bifaces were made on flakes and how many on chunk. Moreover, the Mishash flint at the site, in contrast to Mishash flint raw material exploited elsewhere (e.g., Nesher -Ramla: [41]) is rather amorphous, a fact that further complicates the attempts to assign blank type to a finished tool. Undoubtedly, an amorphous shape of blanks demands from the knapper high flexibility, skill and expertise. High flexibility is reflected by a less standardized reduction sequence and hence expressed in a higher morphological diversity of the bifaces. Taking into consideration all the above, we may assume that the blanks used for the Kaizer biface production were of small size, sometimes flakes and quite amorphous in shape (see GM of bifaces below). Further support for the small size of the blanks and the possibility of their being most probably made on flakes is derived from experimental biface production carried out in order to obtain bifaces similar to those recovered at Kaizer Hill. The experimental knapping produced a variety of debitage items with dimensions similar to those observed in the Kaizer Hill assemblages. Moreover, it appears that the best results derived from flake blanks, frequently of triangular shape with one edge much thicker than the other (in section).

**Fig 31:**
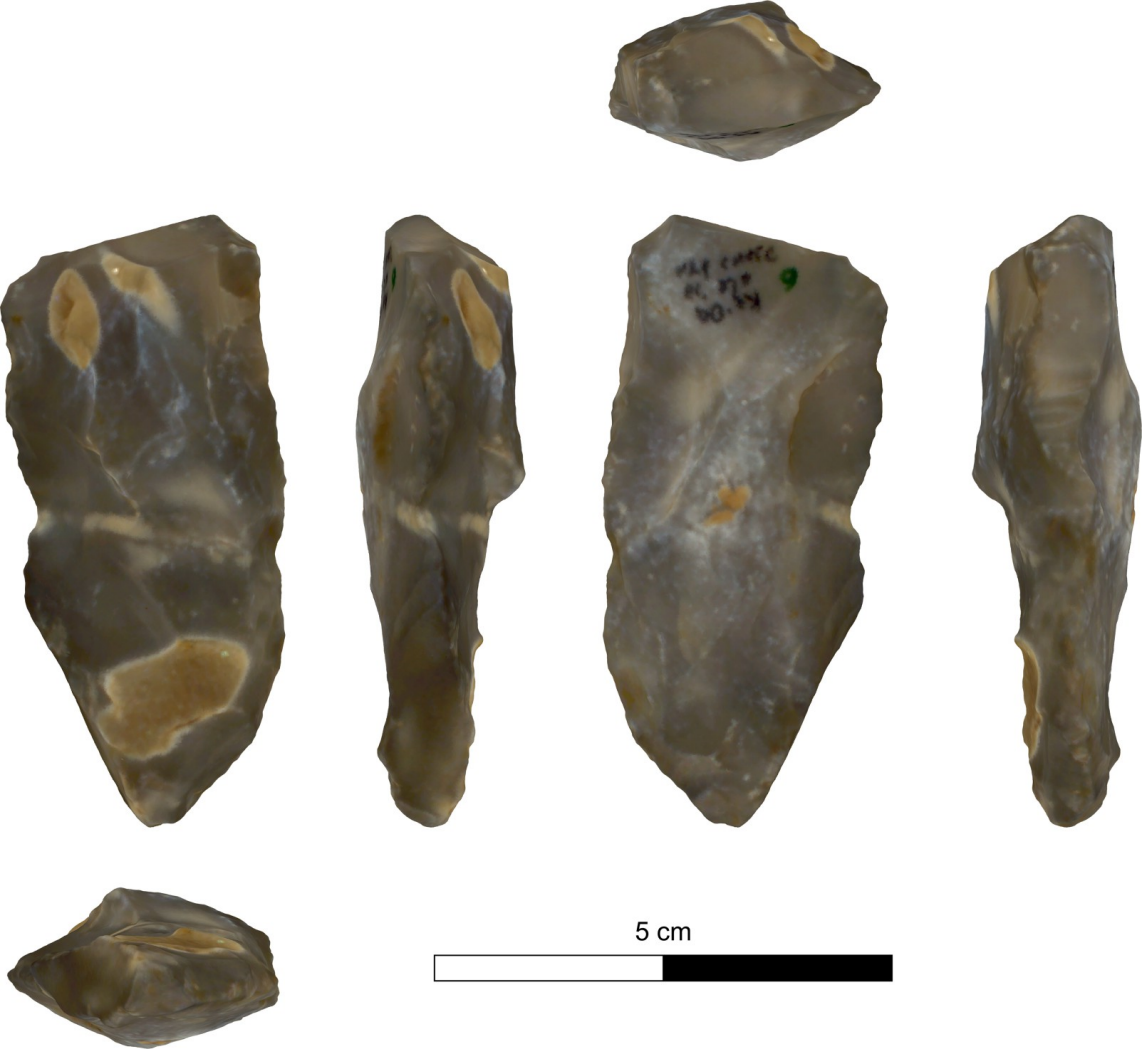
Unfinished biface with preparation for bifacial retouch (#88).

**Fig 32:**
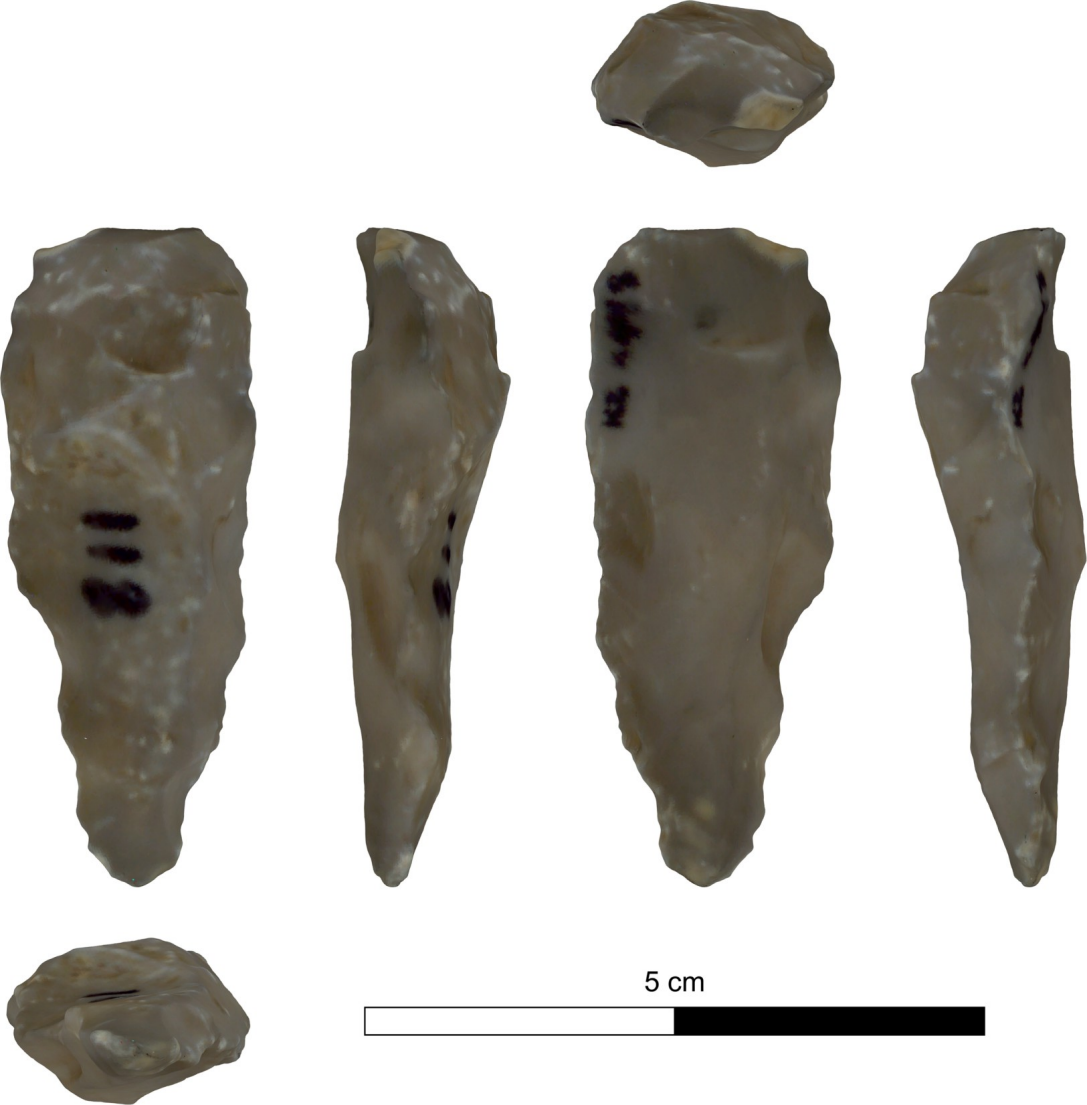
Unfinished biface with preparation for bifacial retouch (#131).

**Fig 33:**
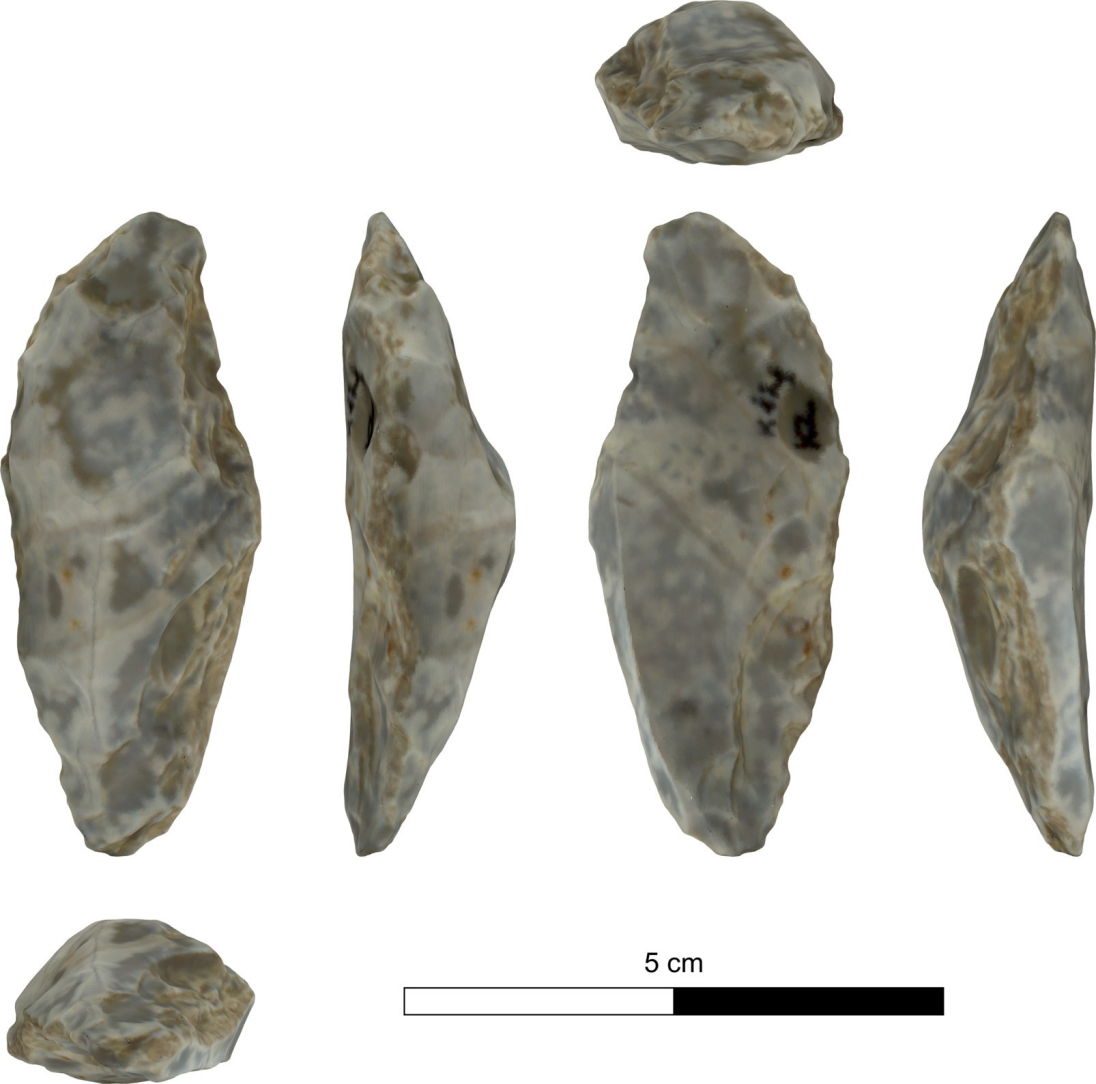
Unfinished biface with preparation for bifacial retouch (#136).

**Fig 34:**
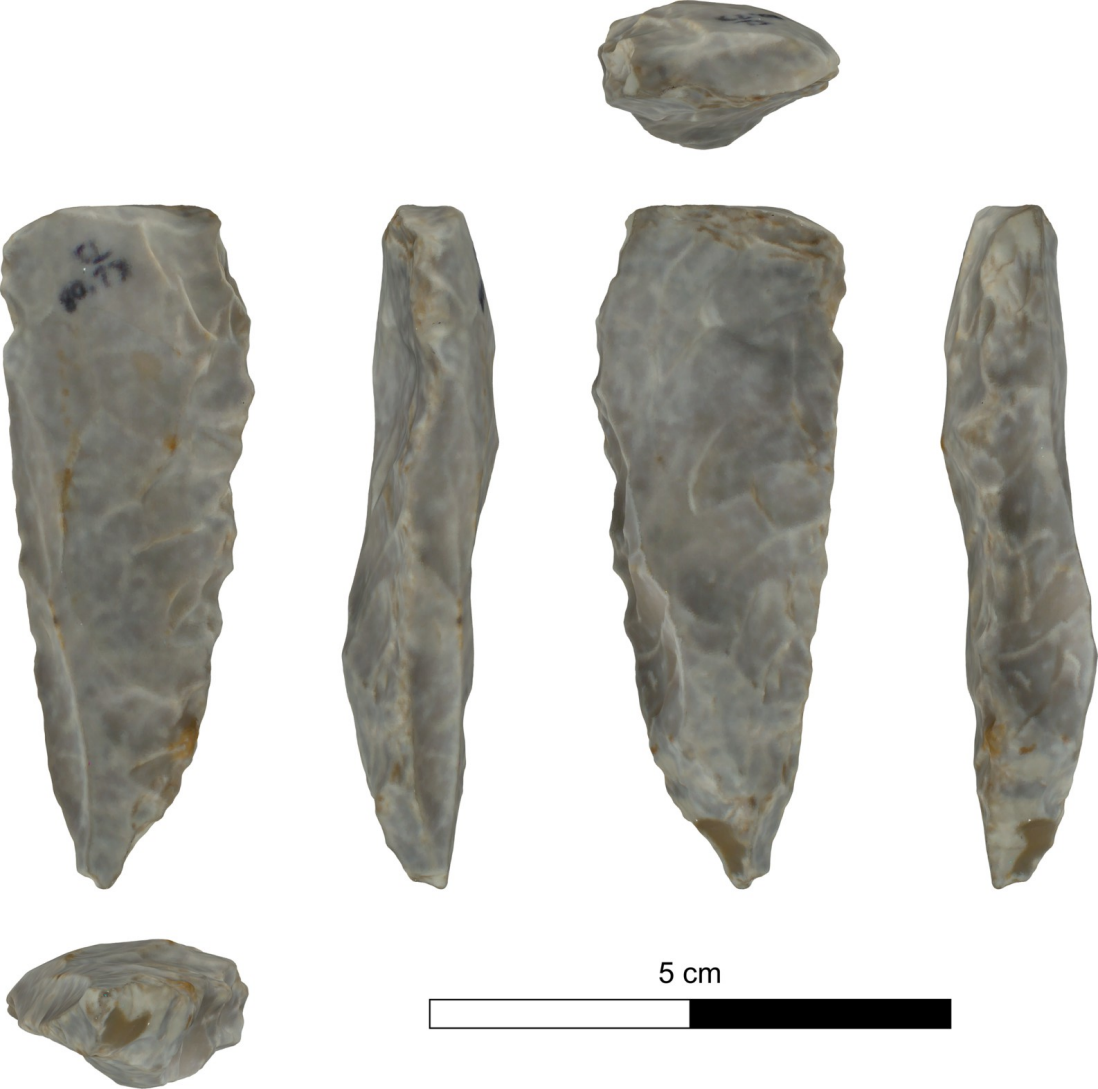
Unfinished biface with preparation for bifacial retouch (#047).

**Fig 35:**
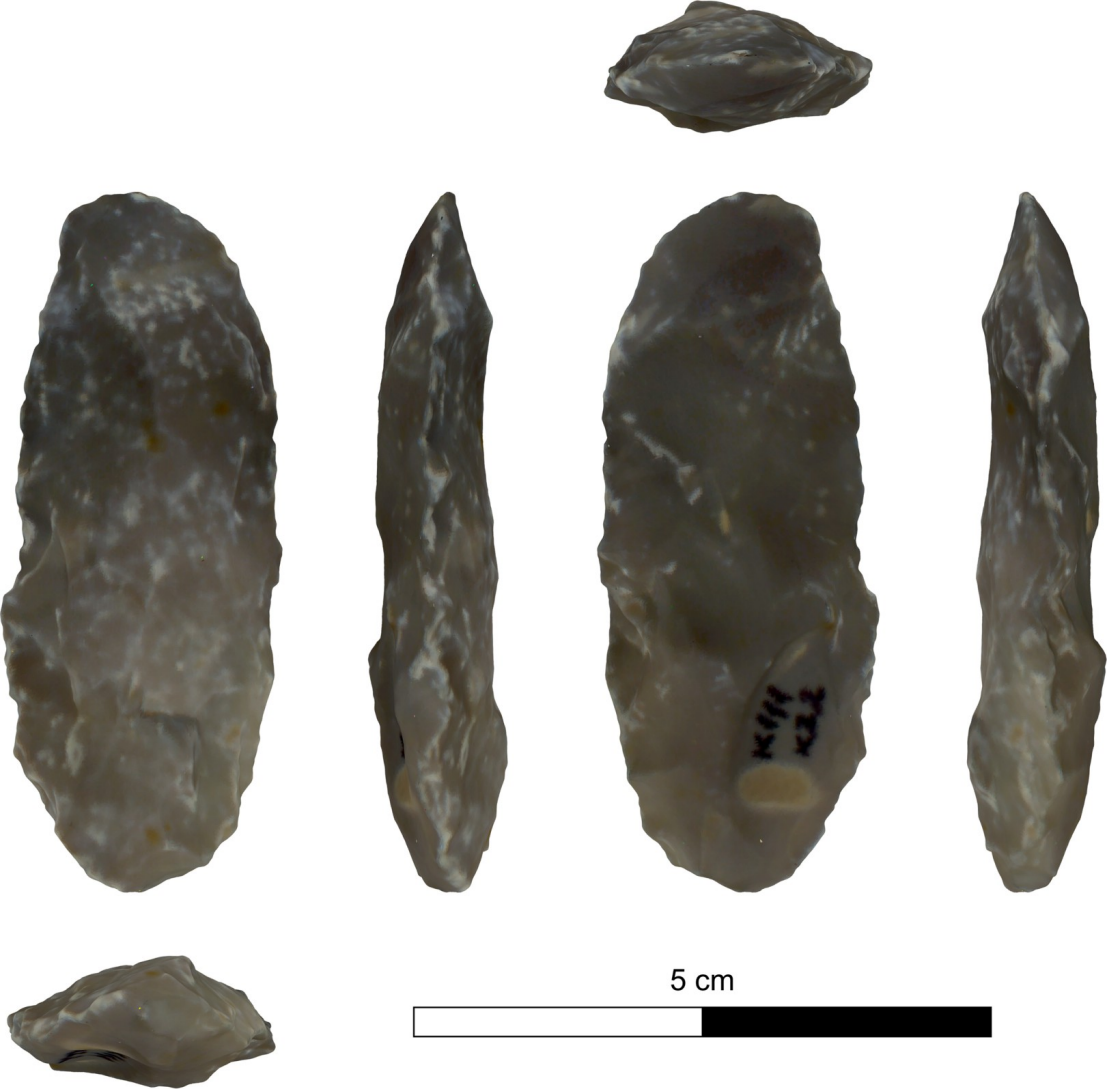
Biface with a transversal scar at the distal end (#106).

The presence of preforms is but one of several features that indicates local production. Other indications

are the presence of residual cortex, and unfinished tools, i.e., a) artifacts with one face entirely flaked while the other is devoid of any signs of bifacial flaking; b) artifacts retaining transversal and lateral preparations for bifacial removals (Table 11; lateral: Figs 31–34; transversal: Fig 35); c) artifacts with the proximal end (frequently of pointed morphology) completely shaped–while other parts of the items are devoid of treatment (Figs 32, 34).

**Table 11:** Bifaces retaining distinct preparation traces.

Terraces—Excavation and Surface	N
Transversal preparation for bifacial removals	9
Longitudinal preparation for bifacial removal (lateral)	12

**Fig 36:**
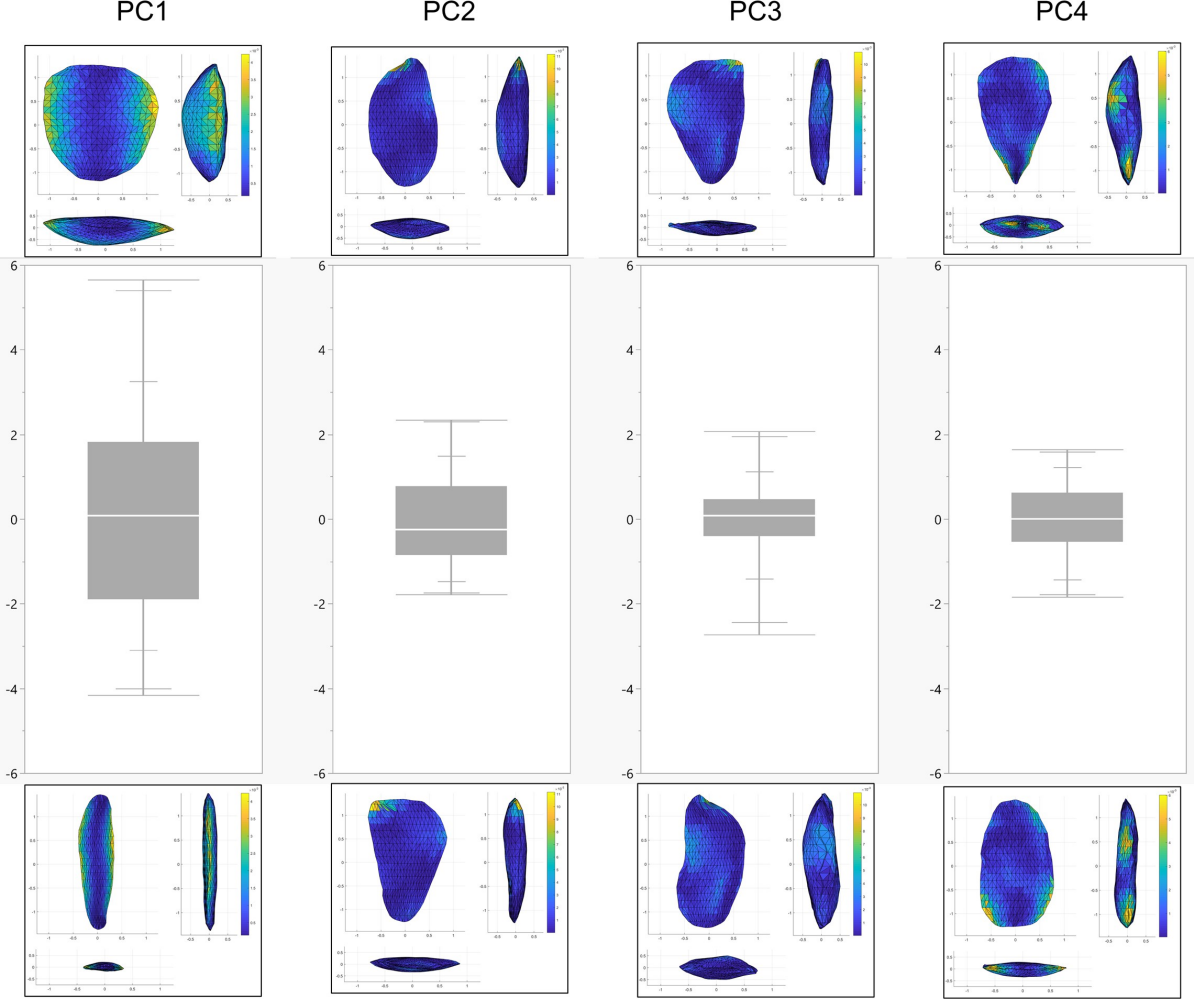
Distribution of the bifaces across each of the first four principal components. Box plots are quantile, blank line is the median. The figures at the top and bottom of each plot represent hypothetical items at the extremity of the principal component to visualize the shape trend. Color scales reflect the proportional spatial distribution of variability on each shape trends (i.e., the areas that change the most).

Biface 3DGM: The 3DGM results describe four significant shape trends each explaining over 5% of the variability, and cumulatively explaining ca. 65% of the sample variability. The first, explaining ca. 44%, ranges between narrow and elongated artifacts to short rectangular ones (Fig 36). The second explaining ca. 9%, ranges between artifacts with left-slanting working edges to artifacts with right-slanting ones (Fig 36). The third explaining ca. 6%, ranges between artifacts with twisted lateral edges to ones with planar ones (Fig 36). The fourth explaining ca. 5%, ranges between artifacts with high distal to proximal width ratio and those with a low one (Fig 36).

The results indicate that no significant differences exist between the two main provenance groups (i.e., Hilltop and Terraces) in terms of mean shape or shape variability. Both groups occupy an almost identical area in shape space and show a highly similar central tendency towards a relatively elongated, proximally pointed and plano-convex artifacts. The spatial variability patterns within both groups are also very similar, concentrating mainly on the distal parts of the lateral edges and within the central area of the tools. In contrast, the distal and proximal surface areas are relatively more homogenous. Furthermore, the pattern in which the variability is distributed across the three spatial dimensions between the groups, is almost completely identical. The only significant difference in morphometric attributes between the Hilltop and Terraces groups is the bilateral asymmetry, where the Hilltop group is significantly more symmetrical than the Terrace one. It should be noted that while the two subgroups originating in the Terraces exhibit some differences in mean shapes and shape variability, these are very minor and statistically insignificant.

**Fig 37:**
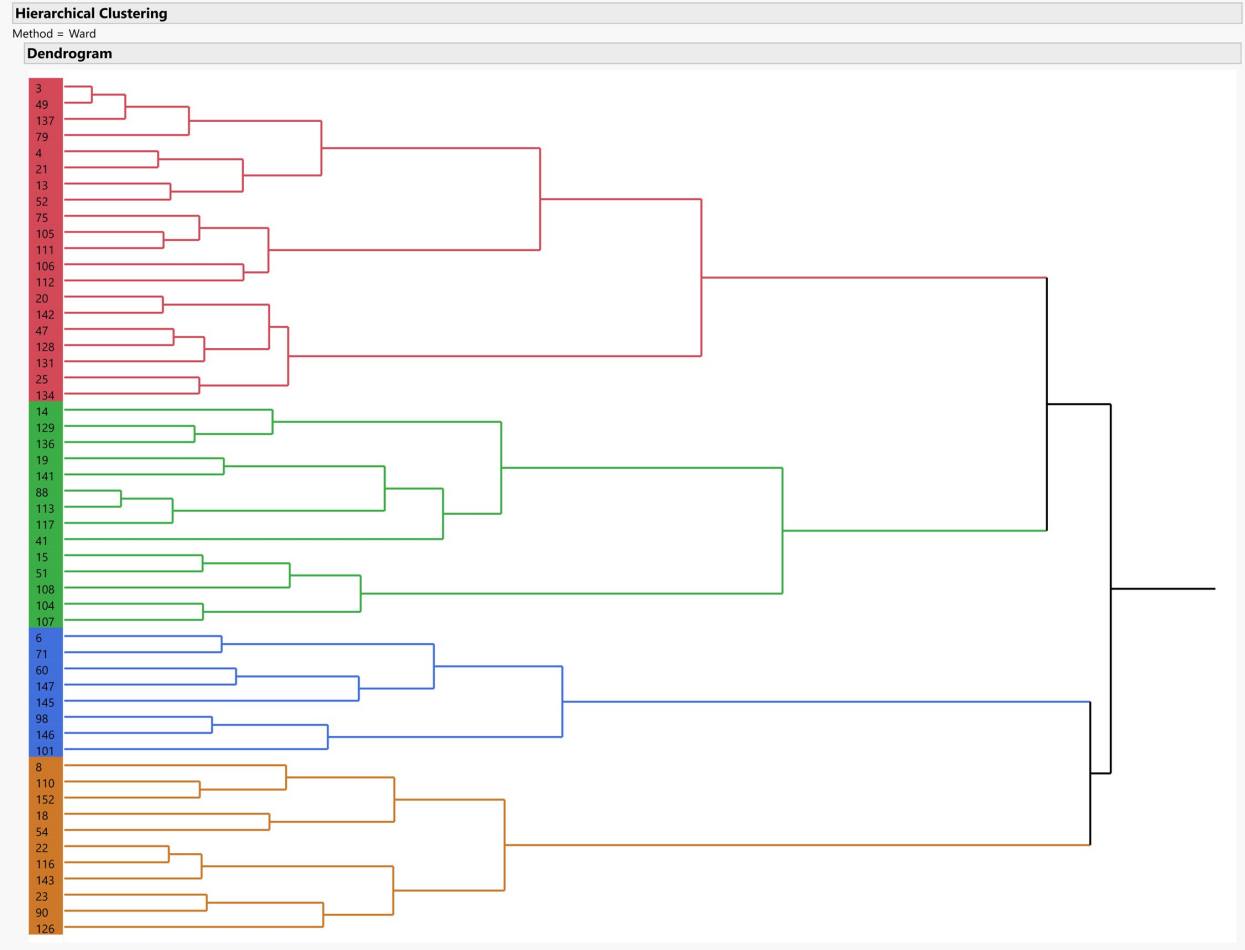
Dendrogram reflecting the hierarchical clustering based on morphological distances between specimens. Colors reflect the four clusters cut-off; labels are artifact numbers.

A hierarchical clustering analysis using Ward’s method, has been performed on the first four principal components (Fig 37). Cubic clustering criterion (CCC) was applied to the results to estimate the optimal number of clusters in the data (SAS Institute Inc., 1983). Its results indicate that while the number of optimal clusters in the data is four, the largest CCC value remains negative for all cluster numbers, suggesting that no natural clusters exist in the data. Furthermore, the mean shapes of all but a single of the suggested clusters fall within the 95% normal confidence ellipses of at least one other cluster.

**Fig 38:**
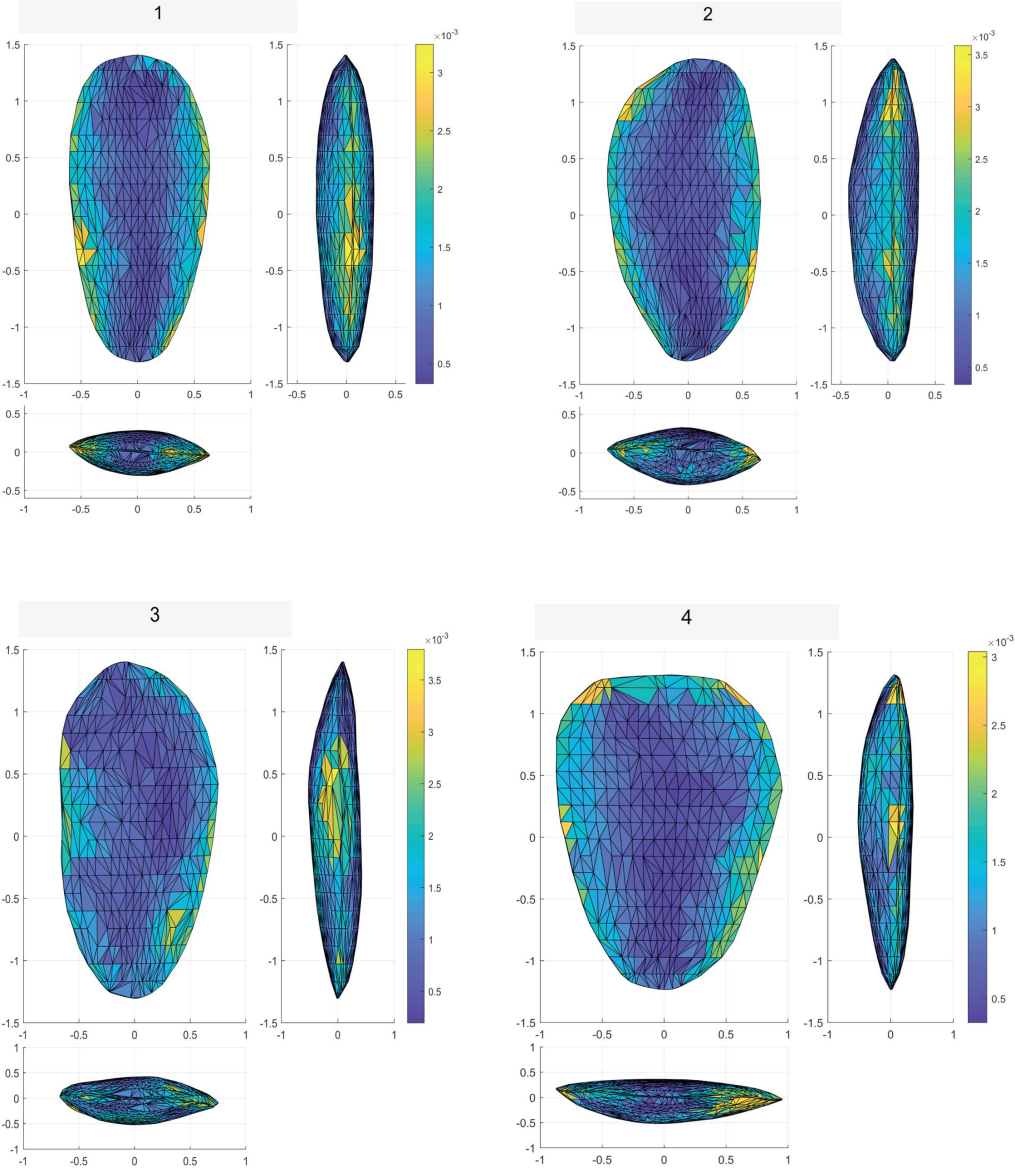
Mean shapes (central tendencies) of clusters 1 (A), 2 (B), 3 (C), and 4 (D). Color scales reflect the proportional spatial distribution of variability in each cluster (i.e., the areas that are most variable).

On the other hand, the mean shapes of each of these suggested clusters significantly differ from all three others, suggesting that the sample does contain some underlying morphological patterns (Fig 38A–38D). A deeper examination of the central tendencies of each cluster in terms of morphology suggests that clusters 1 and 4 differ mainly in the first principal component, reflecting differences between very thin, elongated items with high surface-to-volume ratio in cluster 1 and wide, short, and low surface-to-volume ratio in cluster 4. In contrast, clusters 2 and 3 differ mainly on the second principal component, the shape trend, reflecting differences mainly between left- and right-slanting distal edges. Accordingly, only clusters 1 and 4 are interpreted as reflecting an intentional behavioral tendency in the shapes of the tools. This is based on the nature of the morphological differences, the distance between the clusters’ means, and stylistic and technological observations. In contrast, the differences between clusters 2 and 3 are interpreted as stemming from random, unintentional, differences in tool production or use.

Examination of the differences between clusters 1 and 4 demonstrate some significant differences in several shape trends. Cluster 1 is significantly more homogeneous (Z: 2.62 p-value: 0.009) and refined (Z: -2.37 p-value: 0.01) than cluster 4. Furthermore, its items have significantly straighter, more regular lateral edges (edge curvature: Z: 3.7 p-value: <0.001; edge section irregularity: Z: 2.04 p-value: 0.04), and are also more bifacially symmetrical (Z: 2.66 p-value: 0.008).

Waste products of biface modification: The following paragraphs describe in detail specific debitage artifacts pertaining to the production of the Terraces axes.

The Flint Thinning Flakes: The Terraces assemblages include 405 flint and 50 limestone thinning flakes (Table 12). While all thinning flakes (as the bifaces) are of Mishash flint, the limestone thinning flakes (no limestone bifaces were found) are Bi’na limestone. Worth noting is the fact that the Terraces survey identified quarrying marks on limestone blocks that could be associated with the production of limestone bifaces (Fig 7A and 7B) (see details below).

**Table 12:** Different debitage categories resulting from production of bifaces.

	TERRACES
Biface production by-products	Excavation and Surface
	N
Transversal spall (distal)	38
Longitudinal spall	18
Thinning flakes flint	405
Thinning flakes limestone	50

**Fig 39:**
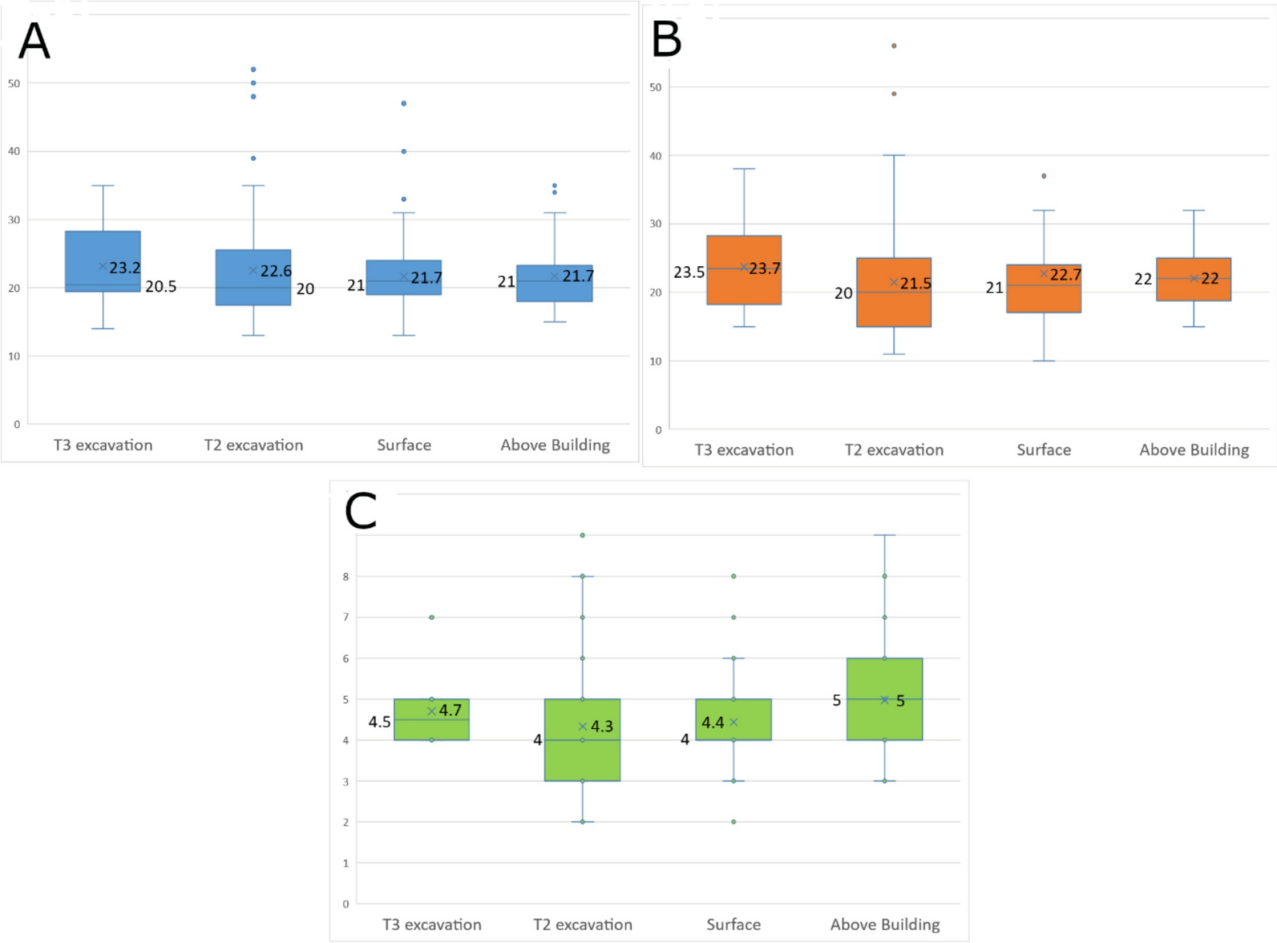
Length (A), width (B), and thickness (C) of flint thinning flakes by assemblage (in mm). The ‘X’ sign represents the mean value of each group, the line within the rectangles represents the median value.

The flint thinning flakes comprise 236 complete items measuring, on the average, 22.0 mm in length (median: 21 mm), 22.3 mm in width (median: 21 mm) and 4.5 mm in thickness (median: 4 mm). The thinning flakes of all four Terraces assemblages present mean metrics fairly similar to each other (Fig 39).

The most common striking platform type of the flint thinning flakes is ‘crushed’ (n = 75; 31.8%), followed by ‘plain striking platform’ (n = 61; 25.8%) and ‘punctiform’ (n = 39; 16.5%) (Table 13). Other types of striking platforms appear in much lower numbers.

**Table 13:** Striking platforms of flint thinning flakes by assemblage.

Type	T2 excavation		T3 excavation		Surface		Above Building		Total	
	N	%	N	%	N	%	N	%	N	%
Crushed	17	23.3	4	40.0	42	36.5	12	31.6	75	31.8
Plain	22	30.1	1	10.0	30	26.1	8	21.1	61	25.8
Punctiform	12	16.4	3	30.0	17	14.8	7	18.4	39	16.5
Missing	8	11.0			6	5.2	4	10.5	18	7.6
Facetted	3	4.1			10	8.7	5	13.2	18	7.6
Indeterminate	6	8.2			4	3.5	-	-	10	4.2
Dihedral	4	5.5			1	0.9	2	5.3	7	3.0
Lipped	-	-	2	20.0	4	3.5	-	-	6	2.5
Removed	1	1.4			1	36.5	-	-	2	0.8
**Total**	**73**	**100.0**	**10**	**100.0**	**115**	**100.0**	**38**	**100.0**	**236**	**100.0**

As for the dorsal faces of the thinning flakes, the most frequent pattern is ‘radial’ (n = 79; 33.5%), followed by ‘simple’ (n = 62; 26.3%) and ‘along the axis & side’ (n = 24; 10.2%) (Table 14).

**Table 14:** Dorsal face scar pattern of flint thinning flakes by assemblage.

Type	T2- excavation		T3- excavation		Surface		Above Building		Total	
	N	%	N	%	N	%	N	%	N	%
Radial	28	38.4	2	20.0	32	27.8	17	44.7	79	33.5
Simple (along axis)	20	27.4	3	30.0	33	28.7	6	15.8	62	26.3
Along axis & side	5	6.8	2	20.0	12	10.4	5	13.2	24	10.2
Along axis & opposed	8	11.0	1	10.0	8	7.0	4	10.5	21	8.9
Convergent	5	6.8	1	10.0	7	6.1	1	2.6	14	5.9
Side	4	5.5	1	10.0	5	4.3	1	2.6	11	4.7
Plain	1	1.4	-	-	8	7.0	-	-	9	3.8
Indeterminate	1	1.4	-	-	2	1.7	2	5.3	5	2.1
Along axis & radial	-	-	-	-	4	3.5	-	-	4	1.7
Parallel	1	1.4	-	-	1	0.9	1	2.6	3	1.3
Cortical	-	-	-	-	1	0.9	1	2.6	2	0.8
Opposed & side	-	-	-	-	1	0.9	-	-	1	0.4
Ridged	-	-	-	-	1	0.9	-	-	1	0.4
**Total**	**73**	**100.0**	**10**	**100.0**	**115**	**100.0**	**38**	**100.0**	**236**	**100.0**

**Fig 40:**
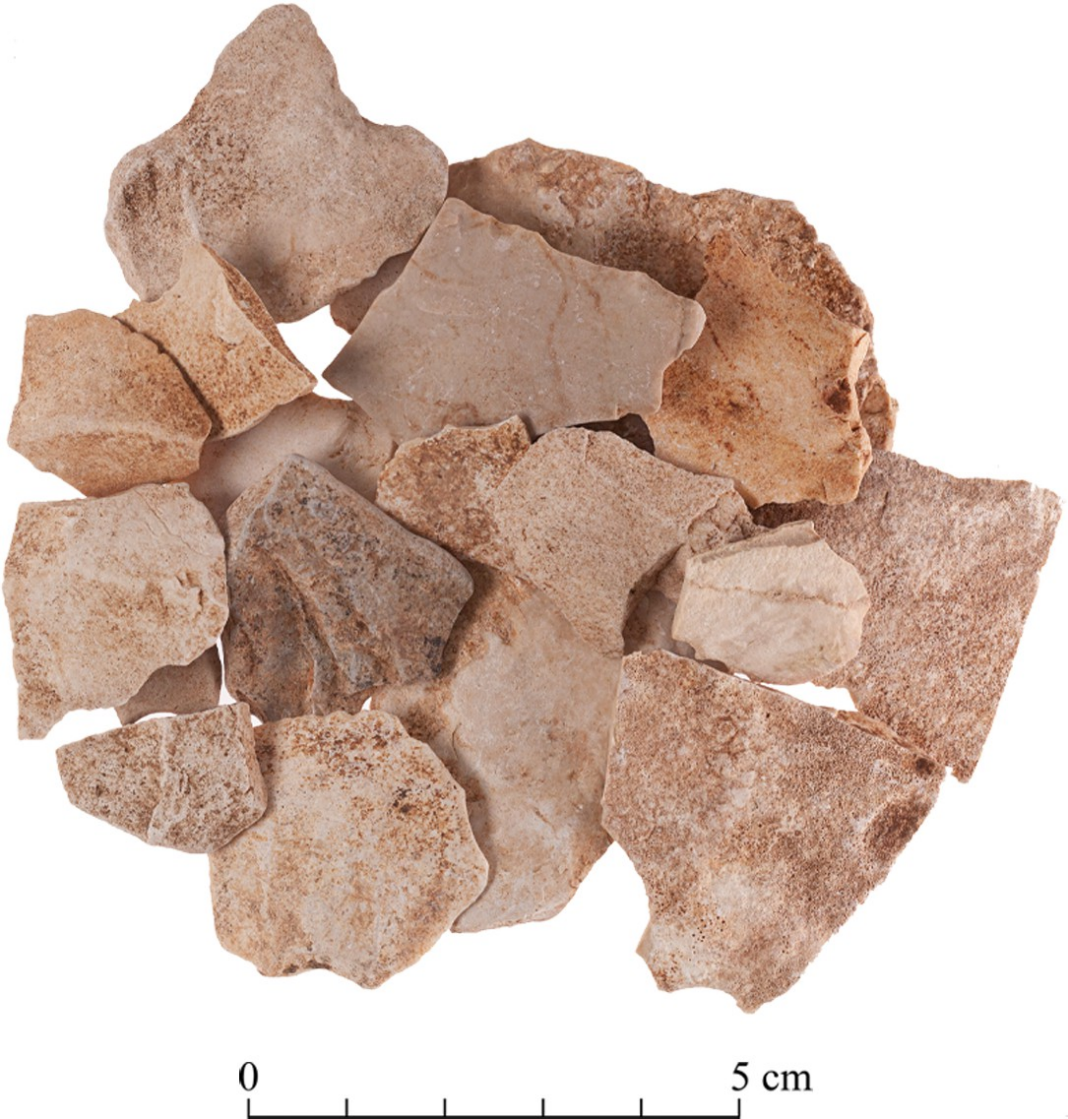
Limestone thinning flakes.

**Fig 41:**
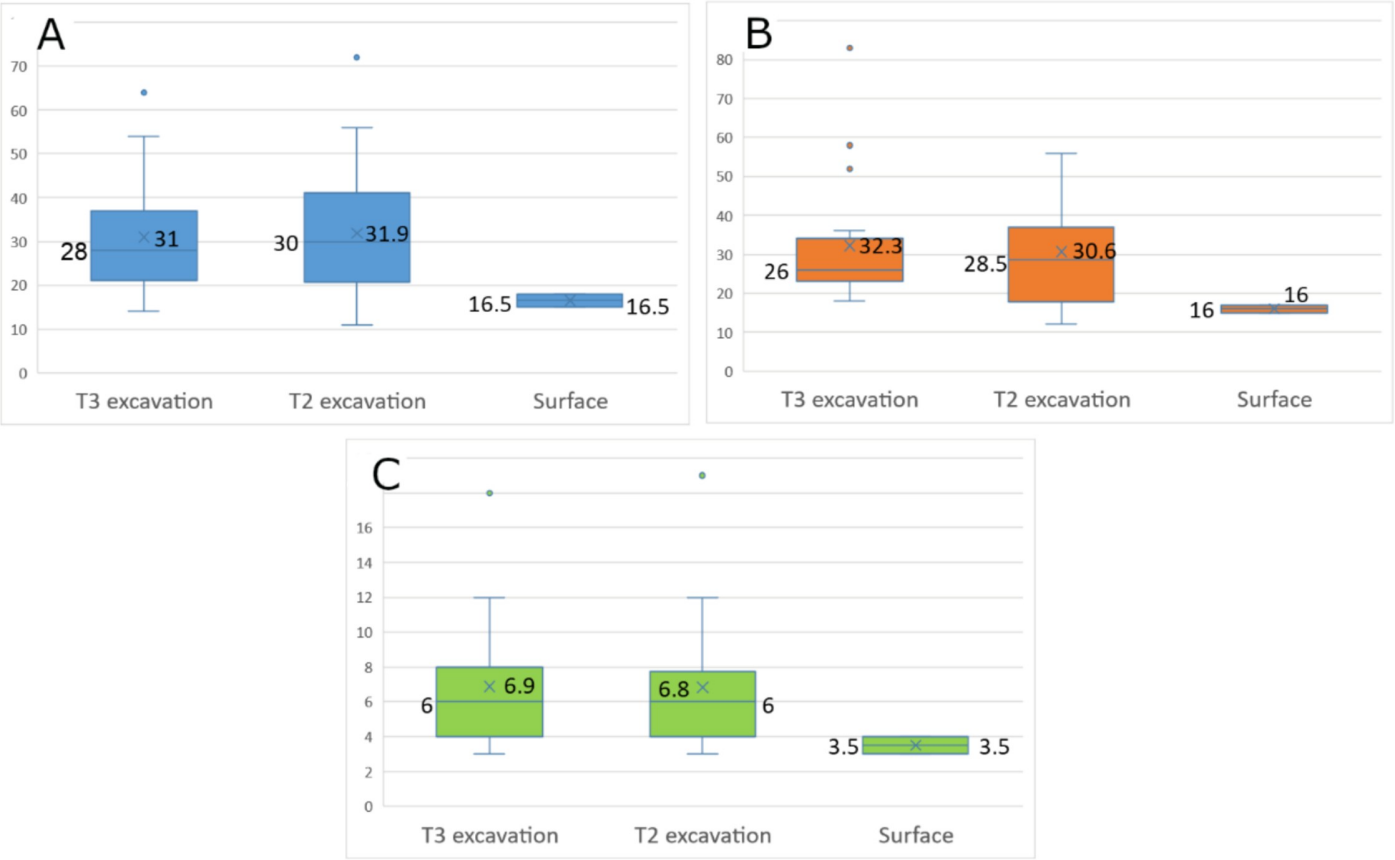
Length (A), width (B), and thickness (C) of limestone thinning flakes by assemblage (in mm). The ‘X’ sign represents the mean value of each group, the line within the rectangles represents the median.

The Limestone Thinning Flakes: The Terraces assemblages comprise 49 complete limestone thinning flakes (Fig 40). Most of them originate from ‘T2 Excavation’ (n = 28), followed by ‘T3 Excavation’ (n = 19). Additional two artifacts were collected from the surface. The limestone thinning flakes tend to be longer, wider and thicker than the flint ones (Fig 41).

## Production of limestone bifaces

We have found evidence that the quarrymen at the Neolithic Kaizer site also produced limestone axes, though the evidence is less extensive than that for the flint bifaces. Rock damage markings indicate that relatively elongated squarish blocks of limestone were systematically removed from the hardest, most compact, Bi’na rocks (Fig 7) which seemingly fit to be used as blanks for bifaces. Limestone biface production is further supported by the presence of limestone typical thinning flakes (Fig 40). While previous discussions about limestone bifaces considered their production solely through pecking, sometimes followed by polishing [24], our findings suggest that their production was more complex and that it involved in the initial stages a reduction process similar to that of the flint bifaces (i.e., producing limestone thinning flakes), differing only in the final stages of modification which is absent at the Kaizer site but present in other PPNA sites (e.g. Netiv Hagdud: [27] and Gesher: [29]).

**Fig 42:**
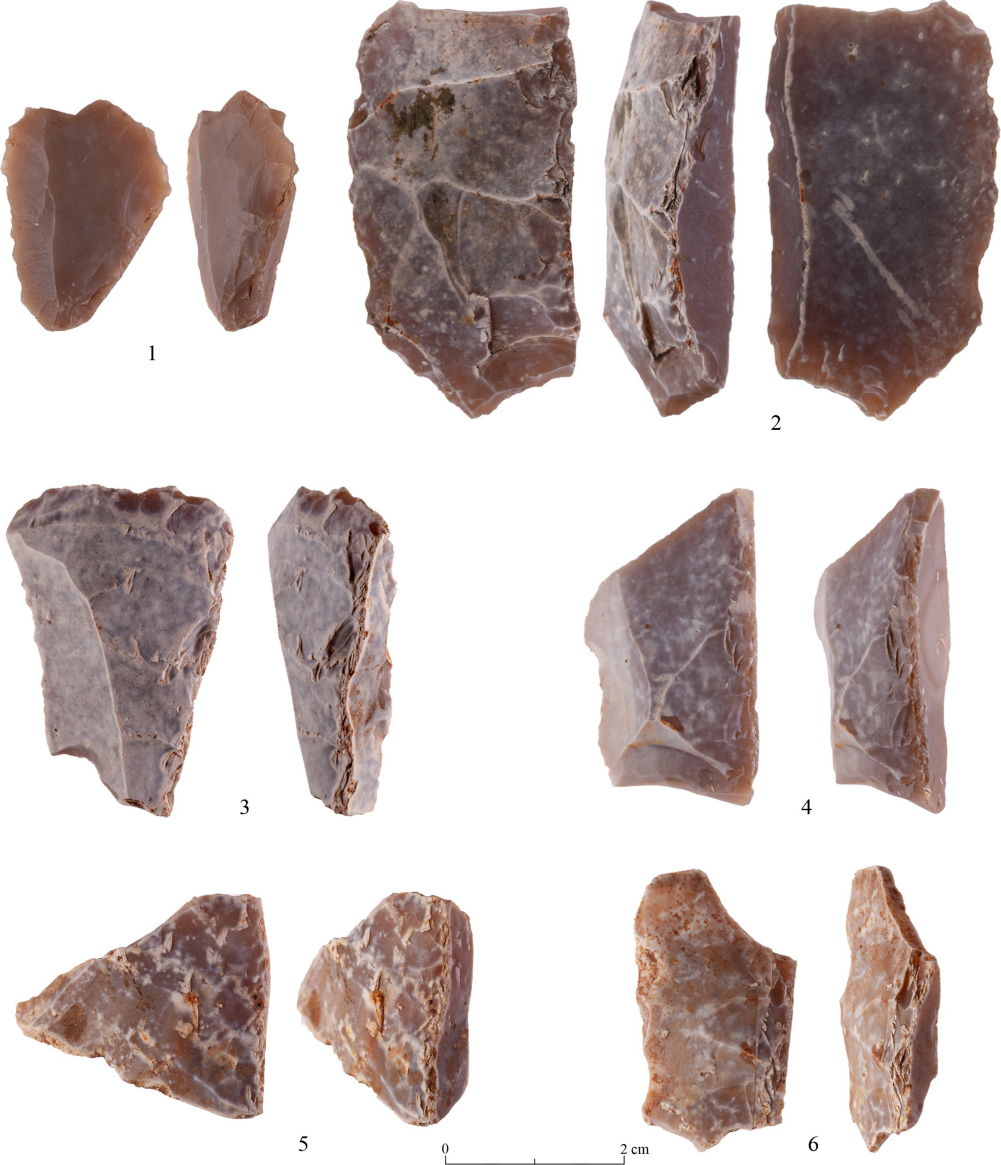
Lateral biface spalls.

Spall: As stated above, these biface waste products resemble in general the ‘classic’ *tranchet* spalls which are assumed to represent the final stage of the tool modification [42], yet it seems that the Terraces spalls reflect earlier phases of the bifaces reduction sequence, having a different section and scar patterns than the former. They bear on their dorsal surfaces remnants of ‘striking platforms’ or alternatively, a bifacial scar pattern. They are also characterized by straight or convex ventral faces, which are frequently at a 90° angle with the dorsal face. We distinguish between two major categories of those spalls (Table 12). One is the transversal spall, which usually has an arched morphology dictated by a concave ventral face and a triangular section, deriving from the modification of the distal (working) part of the biface (Figs 29:1–4 and 30:1–2). The other category is the lateral spall, which originates from the modification of the lateral edges of the tool (Fig 42:1–6). Within the category of the lateral spalls, we further distinguish between artifacts that were flaked-off during the initial stages of the biface modification (Fig 42: 3, 4), and those which derive from the later stages of shaping and thinning of the tool (Fig 42: 1, 2, 5, 6). The first group is frequently characterized by an elongated, curved, triangular or straight spall, while the second are usually very thin flakes, with dorsal faces covered by bifacial scars, typical of an advanced modification stage (Fig 42: 2,6).

### The reduction process of the bifacial tools

Before going in-depth into a suggested reconstruction of the bifaces reduction process (*chaine operatoire*), it should be noted that most of these artifacts originate from surface collections, accordingly this is a description of rather a **fragmented reduction sequence**. The reconstruction is based on the different observations described above. Two other elements were of substantial importance: a) the presence of similar waste products (in particular the ‘spalls’) in both Natufian and Neolithic assemblages with bifaces (e.g., [10, 24, 27, 38, 43]); b) the experiments which we carried out in order to mimic the Kaizer Hill axes production. We believe that the following description of the biface reduction sequence was a known procedure among the Neolithic communities and could be found in other PPN biface assemblages at large.

The small size and the non-standardized shape of the Mishash blanks were a major constraint on the production of bifaces as they for sure deterred from preparing an elongated working edge from which bifacial flaking could take place. Considering the non-standardized shape of the blanks reflected in the high variability of the bifaces, the knappers had to make an effort to obtain an appropriate striking platform for the following stages of modifications. Remains of this preliminary striking platform are still visible on some of the bifaces, though rarely, due to the advanced stages of the tool modification which masked the previous preparations. The preparation of such an edge results in both types of spalls described above. It may also be the origin of some blades and bladelets of the Terraces assemblages which clearly do not derive from the cores recovered on site (Figs 42: 2, 4, 6; 43:3 and 44: 1). Our inventory of this preliminary stage is characterized by short, rather than elongated, lateral spalls due to the size of the tool blanks (see an overshot artifact bearing this technological feature, Fig 44:2). Elongated lateral spalls were reported from the PPNA quarry site of Bir el-Maksur, where abundant and large flint nodules were available [38, 44] (Fig 45). Actually, one can assume that lateral spalls (as well as the transversal spalls described below) probably represent several stages of the biface modification and even biface rejuvenation. Lateral spalls from the early modification stage will have a somewhat triangular section (Fig 42: 1,3,4), while those of the later/final stage will bear on their dorsal face intense, bifacial, shallow flake scars, left by the removal of ‘thinning’ flakes (Fig 42: 2,5,6). Hence, the different types of the lateral spalls testify for a mode of production which involves repeated cycles of striking platform formation and their exploitation throughout the modification process.

**Fig 43:**
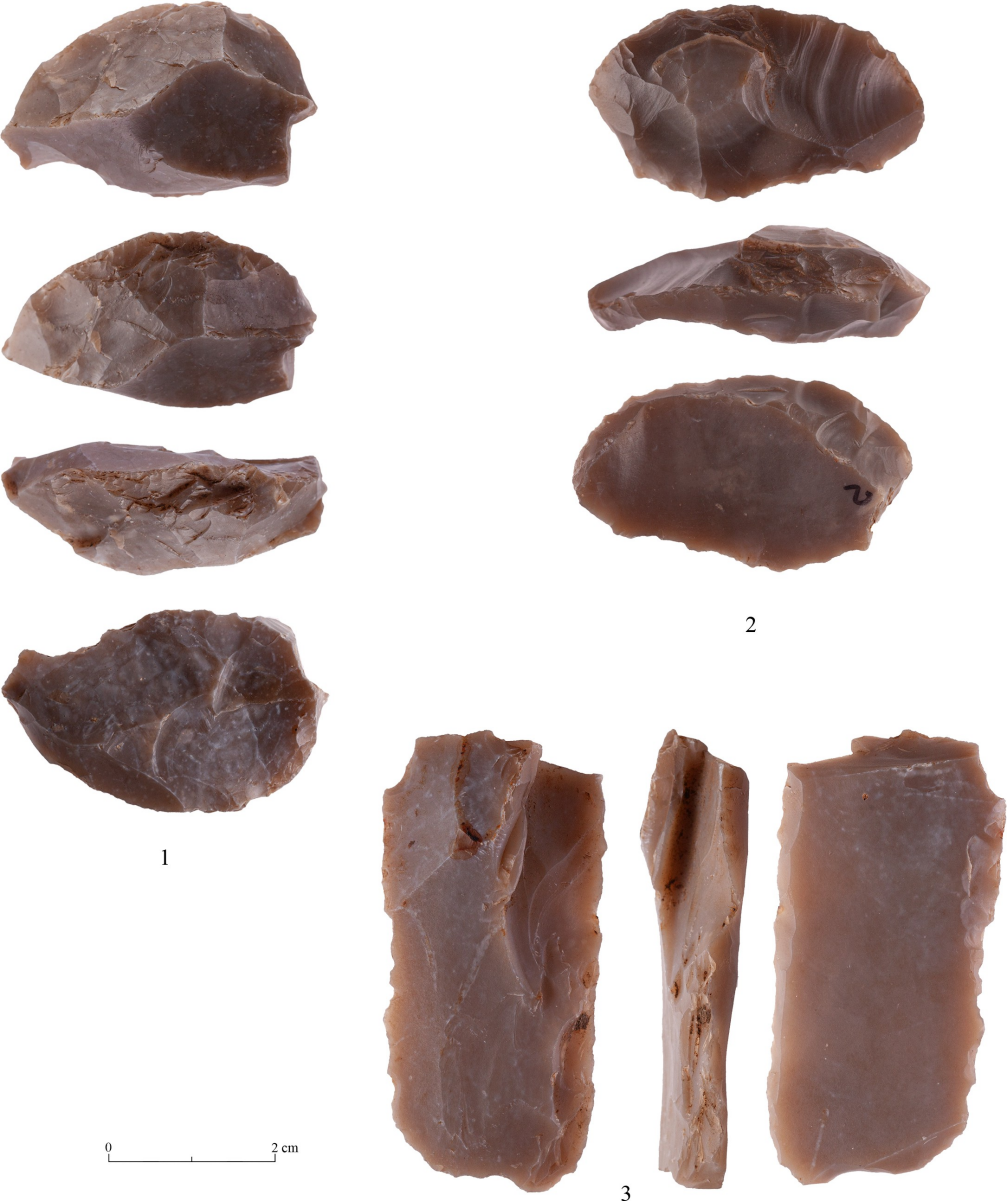
Transversal spalls (1, 2) and a surface-rejuvenation flake (3).

**Fig 44:**
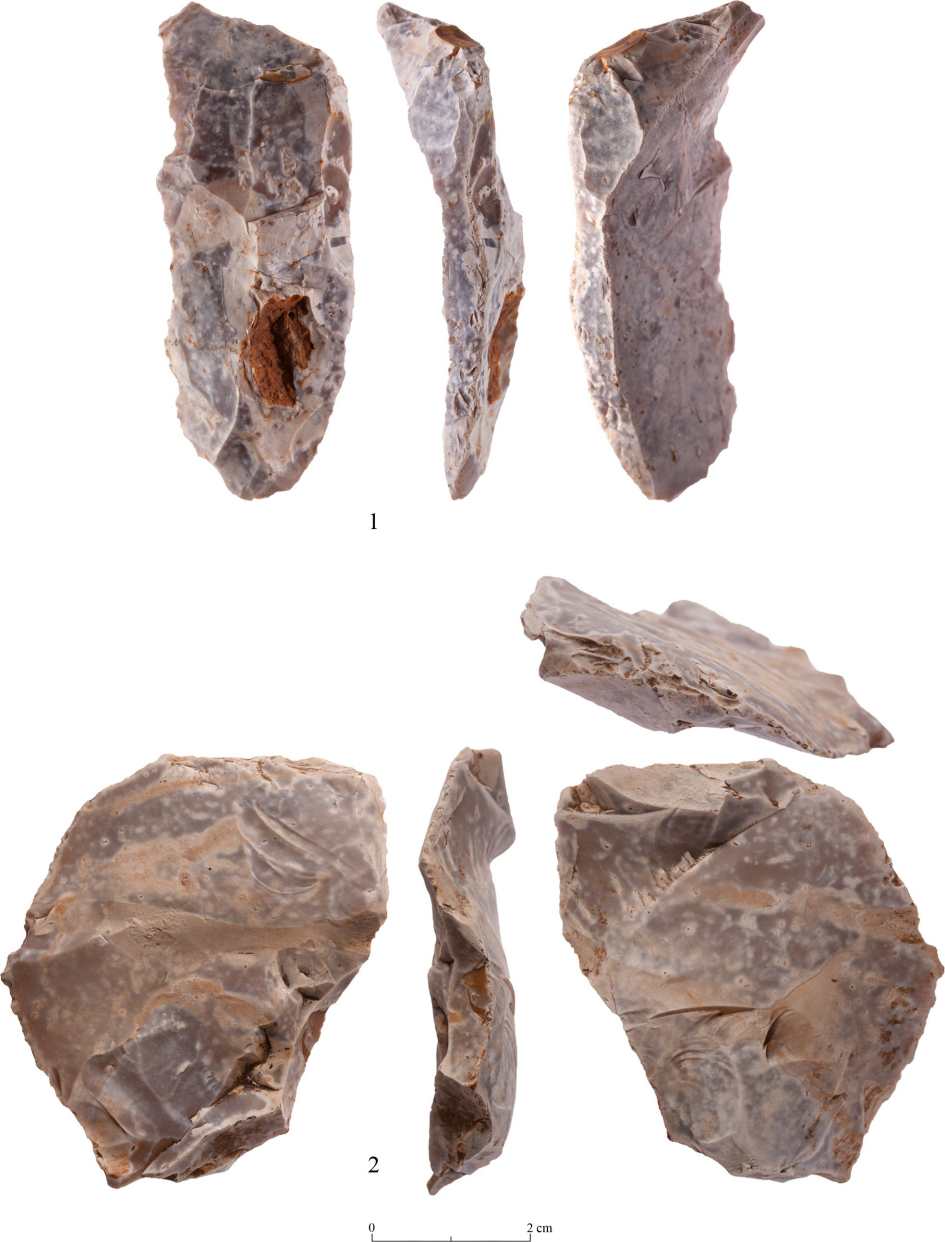
Biface rejuvenation spalls.

**Fig 45:**
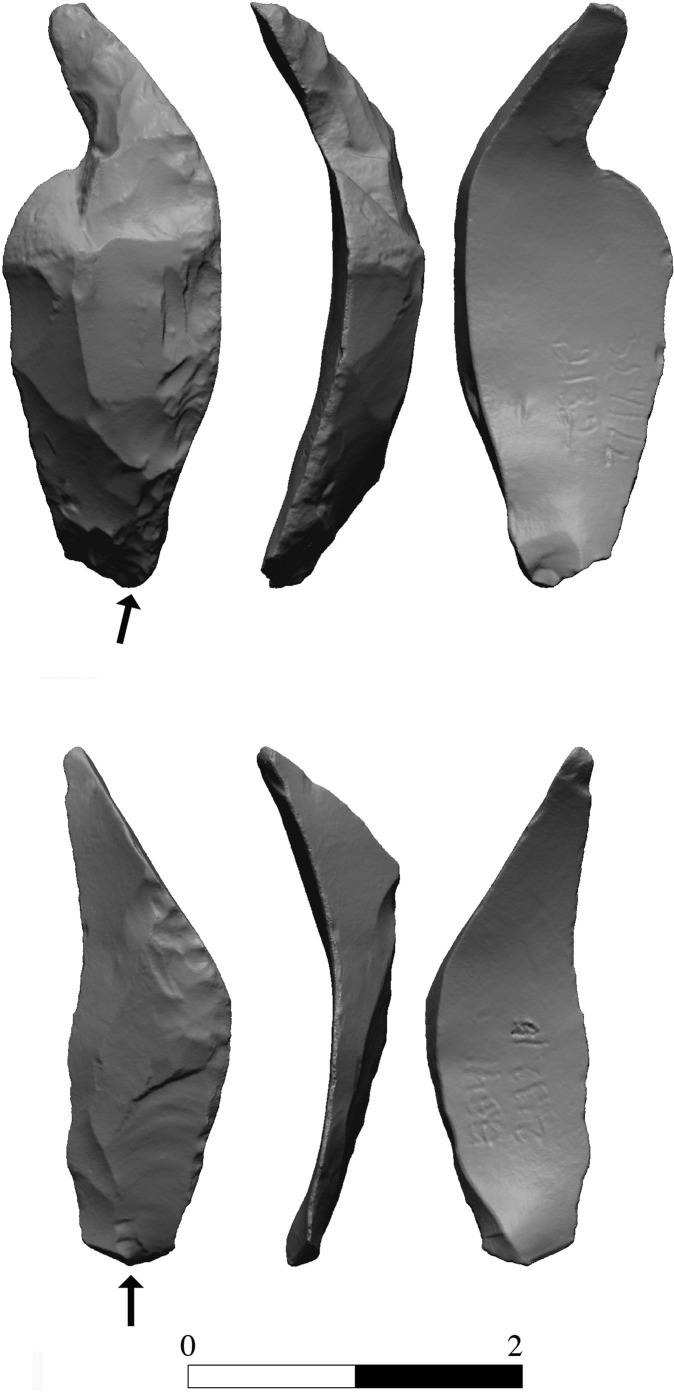
Bir el-Maksur PPNA site—lateral spalls.

Following the preparation of appropriate knapping surfaces is the thinning and finishing of the proximal end of the tool. Our observations support the notion that the knappers first worked on the proximal end and completed its shaping, which in many cases is very thin, small, narrow and pointed (Figs 31 and 32). Only at a later stage of the reduction did the knappers shift towards shaping the distal, working edge of the biface. This is clearly in contrast to other scholars’ view (e.g., [24:14]), who considered the treatment of the distal part (including the *tranchet* blows) as the starting phase of the axe reduction. Here the first goal was to thin the artifact, a procedure which was done through preparing a striking platform on the distal end to enable a series of transversal removals, i.e., transversal spalls (Fig 29: 1–3). This took place either from a single face or from both faces (e.g., Fig 25) resulting in transversal scars (*tranchet* like) either a single-per-face or multiple. The modification of the distal end of bifacial tools by several transversal blows was observed also in other axe assemblages (e.g., [24, 25: 645]).

## Discussion

The Kaizer Hill quarrying rock damage marks show that the Neolithic quarrymen had deep geological knowledge of the two flint-rich Formations available, on the Hilltop (Mishash) and Terraces (Bi’na) of the site and that they were capable of extensive exploitation of these resources. Indeed, the Neolithic quarrymen were versed in the ways and means of handling the hard limestone of the Bi’na Fm. on the Terraces as evidenced by the extensive series of linear quarrying fronts which formulated the terraced morphology of the slopes. The residual flint embedded in the Bi’na Fm. (apparently judged to be of bad quality) and specific quarrying marks provide evidence for intensive exploitation of the extracted flint and to a lesser extent that of the limestone. The techno-typological characteristics of the lithic assemblages retrieved from both the surface and the limited-in-scope excavation indicate that the site belongs to the PPNA cultural sphere.

The location of good quality flint embedded in the caliche at the Hilltop could not be known in advance, as the caliche engulfed the Mishash flint lumps, a residue of a geological eroded surface [11]. The geology of the embedded flint in the Bi’na Fm. on the Terraces represents a different scenario. Here, the flint occurrence is associated with the stratigraphy and original bedding of the limestone bedrock and its structure, as expressed by its joints and fissures. While the search for flint and its quarrying in the Hilltop caliche probably did not have a defined, overall, strategy [11], the search for flint on the Terraces was more methodological and hence the quarrying strategy was most probably planned ahead. It was not only the exposure of surfaces and then a systematic search for the potential flint content, as it involved also a large operation of quarrying, transporting, and stocking of the limestone refuse [21]. Taking into consideration the above and the fact that much planning went into the exposure, evaluation and extraction of the Bi’na flint, evident through the amount of unexploited, residual, flint, left on the exposed surface—it was but natural for us to assume that the recovered chipped stone assemblage will comprise mainly the Bi’na flint. Our study proved us to be wrong as ca. 95% of the artifacts was modified on Mishash flint. This demands new explanations for the overall phenomena observed at the site. It seems unlikely that the Mishash artifacts were washed into their current depositional place. This could have been an option if the assemblage was limited to surface finds. However, the raw material of the excavated artifacts was the same, i.e., Mishash flint. Hence, we suggest that once the hill slopes were stripped off of the high-quality Bi’na flint and the quarrying waste was removed elsewhere (see below), a terraced rocky landscape was formed, and the knapping activities of the Neolithic community took place on the exposed and transformed Bi’na limestone terraced landscape. It is worth noting that there are many more artifacts on the Terraces than on the Hilltop. This could not be explained simply as resulting from downslope erosion since the Hilltop surface did not show any indication of downslope sorting mechanism [11]. If our suggested interpretation is valid, we may consider all of these findings relating to the chipped-flint assemblages as a component of “… the quarrying process included an organized waste management system” [21:401]. We add here yet another facet of the organization, previously unrecorded, where the retrieved raw material was transported from the Hilltop to the Terraces, all within the boundaries of the same quarry site.

The site formation processes which are responsible for building the sedimentological deposits on the Terraces remain unknown. We suggest that the quarrying of the Bi’na Fm. began at the foothill of the slope and advanced upwards. It seems logical to assume that the quarrymen dumped the quarried material down to lower topography as they advanced upslope (the other alternative–dumping material from high topography to a lower one would had necessitated extra effort to get rid of the dumped material and then expose and quarry the underlying rock). Similar strategy is known from much younger quarries ([21] and references therein). What is still unknown is whether the entire excavated content of each Terrace is in-situ or is actually an admixture of both excavated and dumped material. Furthermore, we failed to identify the pristine Neolithic surfaces at the stage when the knapping activities took place. Since nearly all of the artifacts were made on the Mishash flint which is not available on the slopes of the Kaizer Hill, does this mean that all the Bi’na quarrying activities ended before the knapping? Or had the knapping of the Mishash flint took place contemporaneously with the quarrying of the Bi’na Fm.? Our project failed to provide evidence for either alternative as there were no visible concentrations of lithics at the bottom of the trench nor in any other locations along the depositional sequence.

The Terrace lithic assemblages, represent both surface collections (the majority) and excavation material. Since no difference was observed between the surface and excavation material, it has been treated as one assemblage (see Table 15), comprising 12.7% Tools, 81% Debitage, 4% Cores and 152 items with signs of utilization which can be added either to the tools (raising the percentage to 14.9%) or to the debitage (83.3%). The Debris category is nearly double in size from all the rest of the finds and it is nearly all chips (see Table 4). The dominant debitage item (ca. 77%), as well as tool blank, are the flakes, with blade/bladelets poorly represented in both (ca. 4% in the debitage) (for tool blanks see S1 Text), followed by elements pertaining to the production and shaping of artifacts, namely regular CTE, thinning flakes, and bifacial spalls, all in all ca. 25%. Among the cores, the exhausted/amorphic/broken items represent ca. 40% of the total (for details see Tables 5 and 6). The presence of primary elements (ca. 13%), the high percentage of production and modification waste, the intensive exploitation of the cores, and the overwhelming number of chips, all indicate that the knapping on-site was quite intense.

**Table 15:** The entire lithic assemblage—Kaizer Hill Terraces.

CATEGORY	Surface (T0-3)		T2 (excavation)		T3 (excavation)		Surface (above building)		TOTAL	
	N	%	N	%	N	%	N	%	N	%
Tools	346	12.6	144	9.8	65	23.8	316	13.4	871	12.7
Use-wear	46	1.7	17	1.2	6	2.2	83	3.5	152	2.2
Cores	133	4.9	46	3.1	8	2.9	88	3.7	275	4.0
Debitage	2218	80.9	1270	86	194	71.1	1868	79.3	5550	81.1
Debris	5831		4543		527		1535		12436	

* the percentages were calculated excluding the Debris category

As for the tools, they are quite typical for a PPNA industry. The dominant tool category is ‘Perforating tools’ (34.9%). Essentially, their number is much higher as among the ‘Multiple Tools’ category, 53 items are combinations comprising a perforating tool. Specimens in other tool categories may also be considered as re-used or un-finished perforators (e.g., in the ‘Notches and Denticulates’ category and see S1 Text). The 2^nd^ largest category are the ‘Bifaces’ (discussed below), followed by the ‘Notches and Denticulates’. The rest of the tool categories are represented in single percentages (Table 7). Of interest to note the absence of other typical PPNA markers, such as sickles (the only specimen recovered from T3-Excavation, is part of a broken double tool) or arrowheads (the only item that can be considered either as an a-typical arrowhead or a borer was collected from the surface ‘Above Building’).

The absence of those tool types together with the heavy use-wear of the perforating tools and some of the bifaces indicate that the tool inventory, probably of local production, reflects their use in the main activity at the site, namely the quarrying.

## Bifaces

The bifaces of Kaizer are small, and only 24% of them are complete. Over 99% of them are made on Mishash flint and their production took place on Kaizer Hill as manifested through the presence of preforms, many unfinished tools and by an array of waste products, in particular those types characteristic of biface modification e.g., bifacial spalls and thinning flakes. Visually, two groups of bifaces were identified: one skillfully made–symmetrical, and standardized, while the other, the larger group, is characterized by high morphological diversity, by deep scars and irregular edges. Clearly, both groups were made on small and irregular blanks by expert knappers who overcame the inferior quality of the blanks.

The geometric morphometric shape analysis of the bifaces is an inherently comparative approach. Its objective nature and the availability of the obtained data from our study [45] provides a solid foundation for future comparative studies of bifaces which will undoubtedly contribute to a better understanding of this important PPN cultural component.

The 3DGM analysis of the Kaizer Hill bifaces indicated that there are no shape differences between the bifaces of the Hilltop and those of the Terraces. A cluster analysis revealed the existence of four biface clusters, of which of which only two seem to reflect intentional behavioral tendencies in the modification of the tools. Cluster 1 comprises very thin, elongated items with high surface-to-volume ratio, while cluster 4 is comprised of wide, short, and low surface-to-volume ratio tools. It appears that all artifacts interpreted visually as ‘prestigious tools’ were classified to cluster 1. Even though not all objects in this cluster can be considered as such, clearly the morphological attributes of this group reflect a more meticulous and careful production. It should be stressed that allometric analysis indicates that none of the observed differences can be attributed to diverse stages of the tools life history, i.e., usage or re-sharpening.

The small Mishash flint blanks and its characteristically amorphous shapes which was exploited in Kaizer for biface production (and possibly in other biface assemblages in this region, [24], is in sharp contrast to the orderly and larger blanks of raw materials reported from other PPN sites (e.g. Bir el-Maksur; Fig 45). Still, we would like to endeavor and compare the suggested biface reduction process at Kaizer Hill to that of other PPNA assemblages. At Netiv Hagdud Nadel [27] reports that over 50% of the bifacial tools are characterized by more than a single transversal blow and that the size of the “axe spalls” (all in all 156 –[27:110]) are larger than the distal ends of the finished tools. He considers these “axe spalls” as representing re-sharpening and rejuvenation of the tools, yet the sizes of the spalls do not fit those of the complete bifaces: “Many spalls are wider than any tranchet axe” [27:103]. Nadel himself noted that the spalls are not as sharp as the ‘working edge’ of *tranchet* tools. Moreover, Netiv Hagdud “axe spalls” illustrations ([27]: Figs 4.22 and 4.23) show that they resemble the lateral and transversal spalls from Kaizer Hill Terraces. It seems that in Netiv Hagdud, like at Bir el-Maksur, the bifaces are quite large and accordingly their blanks were larger than those observed at Kaizer, thus, the lateral spalls detached during the preliminary stages of the tool modification are elongated, and mostly of blade dimensions. In our view, Nadel’s description of the bifaces with the *tranchet* scars and the “axe spalls” clearly indicates that the latter do not represent the final stage of the axe modification. Rather they, like the Kaizer spalls, represent different stages of production and rejuvenation (Fig 43:1, 2) of the tool. Netiv Hagdud is but one of several similar PPNA assemblages comprising bifaces with analogous scar pattern and waste products (lateral and transversal spalls, thinning flakes) suggesting that the same mode of biface production processes, i.e. the same general reduction strategy, was employed throughout the PPNA times. Accordingly, it seems that the reduction processes (though fragmented) suggested for the Kaizer Hill bifaces are valid for other Neolithic assemblages as well.

Barkai [24] refers to the site Shimshoni located not far from Kaizer, as an axe workshop, typical of other sites in the vicinity of Modi’in and claims that such sites were mainly producing artifacts that were exported elsewhere. While we consider the Kaizer Hill site as both a quarry and an axe workshop we disagree with Barkai’s interpretation. We consider the sites of Kaizer Hill, Shimshoni, Bouchman and most probably other sites in the region, as quarrying sites, where the production and utilization of bifaces was primarily as quarrying tools. We agree with Barkai that the bifaces found in the Modi‘in sites are smaller and thinner than those from other PPNA sites (e.g., Netiv Hagdud), but we disagree with his interpretations concerning the function of the tools, as he believes that bifaces were used for: “… cut[ting] shrubbery or perhaps small trees as well as light woodworking tasks. … they may have also taken place in the preparations for the cultivation of wild cereals.” [24: 369]. We believe that the high breakage rate of the bifaces at Kaizer as in other assemblages mentioned above [24] and the intense use-wear observed with the naked eye supports the notion that mostly these were quarrying tools. Interestingly, while most of the Kaizer bifaces were indeed used as heavy-duty utensils, a small percentage of the tools, of a much better quality and with no use-wear to speak of were most probably ‘prestige items”, to be exported to other PPNA sites.

## Concluding remarks

The PPNA is rather a problematic period when trying to figure out the inter-relationship between the various site types, i.e., we have very little knowledge as regards of the ways and means the raw material, quarried in Kaizer Hill site, was transferred and integrated within the PPNA sites networking. The reasons for this vary from the short time span of the PPNA to its basic definition as both a timeframe and cultural entity/entities (and see [46]). Perhaps also in the PPNA there were ‘nodes of production’ as those known from the following PPNB, e.g., sites such as Ramat Tamar [47] which were knapping stations where the quarried material was fashioned into final products, as those recovered from PPNB settlement sites.

It appears that the Mishash flint was quarried on the Hilltop, partly exploited there [11] and partly moved locally to the stripped, barren-rocky Bin’a quarried Terraced slopes. Apparently, the Mishash flint was modified and used locally, most obviously the bifaces, used as one of the main quarrying tools. The crude marksmanship of these ‘working’ tools most probably reflects the aim of the local PPNA craftsmen to avoid excessive investment in the tools’ shape, as the bifaces break easily when used, as was observed in other quarrying sites where in our opinion bifaces were working tools, part of the quarrying activities (e.g., the Shimshoni site, [24]). We believe that the local knappers were highly skilled in producing useable bifaces from amorphous and small sized blanks. This expertise is also expressed by the presence of some ‘prestigious’ specimens, well-made bifaces, similar to items recovered in basecamp sites (e.g., Netiv Hagdud [27], Gesher [29]). Indeed, these tools provide a glimpse of the unknown PPNA distribution system of finished tools. We also miss part of the limestone exploitation processes evident at Kaizer Hill. Limestone blocks were quarried and flaked *in situ* on the Kaizer Terraces, yet, no limestone bifaces were recovered, indicating most probably that the final products were exported elsewhere. Still, we can state based on the limestone debitage recovered on site that the reduction sequence of the limestone bifaces, at least in Kaizer Hill was the same as that of the flint bifaces.

The PPNA sites vary not only in their geographic location and environmental settings, but also in their extent, intensity and functionality, from hunting/temporary camps [46, 48, 49] through hamlets (e.g., Netiv Hagdud), regional centers serving as focal points or nodes of exchange, with public communal (sometimes monumental) structures (e.g., the Tower and Walls at Jericho (e.g., [50]) or the large-scale communal ‘kiva-like’ edifice at Wadi Faynan 16 [51, 52] and last but not least major quarry sites, some of which were mentioned herewith (e.g., Tzur Natan, Bir el-Maksur, Kaizer and its vicinity). Clearly the PPNA quarrymen had extensive knowledge of the environment, the diverse rock resources, and the various options of their manipulation. Another example of this is the production of caliche slabs at the site of Hatula [9]. These slabs were obtained through a particular technique (mainly trenching), while the search on-site for good quality flint embedded within the caliche was conducted mainly through drilling.

Actually, the Hatula site, just like the site of Kaizer Hill portrays a major alteration of the landscape, though different in nature and at the latter, of a much larger magnitude. The different approaches of exploiting bedrock resources illustrate both the detailed knowledge and the adroitness of the PPNA communities.

The south Levantine PPNA sites exhibit an array of architectural features, both domestic and communal ([46, 53] and references therein), expertly constructed from both stone and mud-bricks as well as the intensive use of plaster.

There are relatively few quarries that were exploited only during the PPNA (all of which are mentioned in the present study), as more frequently similar quarries were used for protracted periods by different cultural entities (e.g. [54, 55] and references therein). Yet, it is during the PPNA that one can observe a rise in the size and scale of quarrying enterprises, culminating in the spectacular phenomena of PPNA pillar quarrying in the Northern Levant, southeastern Anatolia, at sites such as Gobekli Tepe [56] and [57]. Clearly the accumulated knowledge, expertise and communal memory of exploitation of resources served as a solid foundation for the growing architectural endeavors in the early Neolithic at large all over the Levant.

The PPNA phenomena in the southern Levant, which is the focal area of our discourse is varied, differing in some aspects from the preceding Epipalaeolithic cultural realm and heralding the appearance of the fully fledged agricultural existence developing through the PPNB and later periods. Indeed, the PPNA cultural complexity and its variability is impressive and most meaningful from the perspective of cultural evolution ([46] and references therein). Our study of the Kaizer Hill quarry provides but a glimpse into a single facet of the PPNA complexity. Taking into consideration the amount of the quarried raw material we clearly miss an important segment of data concerning where that huge quantity of raw materials was transferred to and deployed. Apparently, there is a lot more to be explored in order to obtain a fuller picture of the PPNA make-up and cultural connectivity. Accordingly, detailed study of such quarry sites as Kaizer Hill provide new directions for future explorations, clearly pointing out the ‘black holes’ of the PPNA archaeological research.

## Supporting information

S1 File Detailed methodology and descriptions of the selected Rock Damaged Units (S1 Text and S1 Figs 1–7). (DOCX)

S2 File (DOCX)

S1 Text KAIZER—detailed tool typology. (DOCX)

## References

[1] Chew SC. World Ecological Degradation: Accumulation, Urbanization, and Deforestation, 3000BC-AD2000: Altamira Press; 2001.

[2] Barkai R, Gopher A, LaPorta PC. Middle Pleistocene landscape of extraction: quarry and workshop complexes in northern Israel. In: Goren-Inbar N, Sharon G, editors. Axe Age Acheulian Tool-Making from Quarry to Discard. London: Equinox Publishing; 2006. p. 7–44.

[3] Aston M. Interpreting the landscape: landscape archaeology and local history: Routledge; 2002.

[4] Bender B, Hamilton S, Tilley C, Tilley CY, Anderson E. Stone worlds: narrative and reflexivity in landscape archaeology. Walnut Creek, Ca.: Left Coast Press; 2007.

[5] David B, Thomas J, editors. Handbook of landscape archaeology. London: Routledge; 2016.

[6] Herzlinger G, Goren-Inbar N. Do a few tools necessarily mean a few people? A techno-morphological approach to the question of group size at Gesher Benot Ya’aqov, Israel. Journal of Human Evolution. 2019;128:45–58. doi: 10.1016/j.jhevol.2018.11.008 30825981

[7] Barkai R, Gopher A, Weiner J. Quarrying flint at Neolithic Ramat Tamar—An experiment. In: Astruc L, Binder D, Briois F, editors. Technical Systems and Near Eastern PPN Communities. Antibes: Éditions SPDCA; 2007. p. 25–32.

[8] Finkel M, Gopher A, Barkai R. Extensive Paleolithic flint extraction and reduction complexes in the Nahal Dishon central basin, Upper Galilee, Israel. Journal of World Prehistory. 2016;29:217–66.

[9] Grosman L, Goren-Inbar N. “Taming” rocks and changing landscapes: A new interpretation of Neolithic cupmarks. Current Anthropology. 2007;48(5):732–40.

[10] Marder O, Goring-Morris AN, Khalaily H, Milevski I, Rabinovich R, Zbenovich V. Tzur Natan, A Pre-Pottery Neolithic A site in central Israel and observations on regional settlement patterns. Paléorient. 2007;32(2):79–100.

[11] Grosman L, Goren-Inbar N. Landscape Alteration by Pre-Pottery Neolithic Communities in the Southern Levant–The Kaizer Hilltop Quarry, Israel. PLosONE. 2016;e0150395. doi: 10.1371/journal.pone.0150395 26960156 PMC4784956

[12] Zbinovich V. Modi’in Project -The Flint Assemblage from the Hill (A) at Modi’in (1). Jerusalem: Israel Antiquities Authority; 1–8; 1999a.

[13] Zbinovich V. Modi’in Project -The Flint Assemblage from the Early Neolithic Site F9 at Modi’in—Shimshoni. Jerusalem: Israel Antiquities Authority; 1999b.

[14] Zbinovich V. Modi’in Project—Salvage Excavations at the Early Neolithic (PPNA) Site F3 at Mod’in-Buchman (Continuation). Jerusalem: Israel Antiquities Authority; 1999c.

[15] Zbinovich V. Salvage excavations at a Pre-Pottery Neolithic site at Modi’in. Atiqot. 2006;51:1–14.

[16] van den Brink ECM. Evaluation and assessment of salvage excavations at Modi’in-Buchman, Southeast Precinct, Hills ’B’ and ’C’, Fall/Winter 2004. Israel Antiquities Authority; 2004. Report No.: # A/4069-04/01.

[17] van den Brink ECM, Liphschitz N, Lazar D, Bonani G. Chalcolithic Dwelling Remains, Cup Marks and Olive (Olea europaea) Stones at Nevallat. IEJ. 2001;51:36–43.

[18] Golani A. Salvage excavations in the Modi‘in landscape. Atiquot. 2005;50:73–97.

[19] Spivak P. Modi‘in, Giv‘at Kaizer. Hadashot Arkheologiyot—Excavations and Surveys in Israel. 2016;128.

[20] Y. Yechieli, cartographer Geological Map of Israel at 1:50,000—sheet 8-III, Lod. Jerusalem: Geological Survey of Israel, Ministry of National Infrastructures; 2008.

[21] Herzlinger G, Grosman L, Goren-Inbar N. The PPNA Quarry of Kaizer Hill, Modi’in, Israel—The waste piles. In: Borrell F, Ibanez JJ, Molist M, editors. Stone Tools in Transition: From Hunter-Gatherers to Farming Societies in the Near East. Bellaterra (Barcelona): Universitat Autònoma de Barcelona. Servei de Publicacions; 2013. p. 395–405.

[22] Novoselsky I, Grosman L, Herzlinger G, Goren-Inbar N. Limestone Wedges: ad hoc Quarrying Tools of the Kaizer Hill Quarry Site. Lithic Technology. 2020:45:68–85.

[23] Grosman L. The Natufian Chronological Scheme–New Insights and their Implications. In: Bar-Yosef O, Valla F, editors. Natufian Foragers in the Levant—Terminal Pleistocene Social Changes in Western Asia. Archaeological Series 19. Ann Arbor, Michigan: International Monographs in Prehistory; 2013. p. 622–35.

[24] Barkai R. Flint and Stone Axes as Cultural Markers: Socio-Economic Changes as Reflected in Holocene Flint Tool Industries of the Southern Levant. Berlin: ex oriente; 2005.

[25] Crowfoot-Payne J. The Flint Industries of Jericho. In: Kenyon KM, Holland TA, editors. Excavations at Jericho, V Appendix C. London: British School of Archaeology in Jerusalem; 1983. p. 622–759.

[26] Barkai R. The evolution of Neolithic and Chalcolithic woodworking tools and the intensification of human production: axes, adzes and chisels from the Southern Levant. In: Davis V, Edmonds M, editors. Stone Axe studies III Oxford: Oxbow; 2011. p. 39–54.

[27] Nadel D. The chipped stone industry from Netiv Hagdud. In: Bar-Yosef O, Gopher A, editors. An Early Neolithic Village in the Jordan Valley, Part I: The Archaeology of Netiv Hagdud. Cambridge: American School of Prehistoric Research; 1997. p. 71–149.

[28] Bar-Yosef O, Goring-Morris AN, Gopher A, editors. Gilgal- Early Neolithic Occupations in the Lower Jordan Valley: The Excavations of Tamar Noy. Oxford: Oxbow Books; 2010.

[29] Garfinkel Y, Nadel D. The Sultanian flint assemblage from Gesher and its implications for recognizing Early Neolithic entities in the Levant. Paléorient. 1989;15(2):139–51.

[30] Herzlinger G, Grosman L. AGMT3-D: A software for 3-D landmarks-based geometric morphometric shape analysis of archaeological artifacts. PLOS ONE. 2018;13(11):e0207890. doi: 10.1371/journal.pone.0207890 30458049 PMC6245792

[31] Herzlinger G, Varanda A, Deschamps M, Brenet M, Lopez-Tascon C, Goren-Inbar N. Reevaluation of the Classification Scheme of the Acheulian in the Levant– 50 Years Later: A Morpho-Technological Analysis of Handaxe Variability. PaleoAnthropology. 2021. 10.48738/2021.iss1.70.

[32] Agam A. Flint Procurement and Exploitation Strategies in the Late Lower Paleolithic Levant: the case of Acheulo-Yabrudian Qesem Cave. Tel Aviv: Tel Aviv University; 2019.

[33] Agam A. Late Lower Paleolithic lithic procurement and exploitation strategies: A view from Acheulo-Yabrudian Qesem Cave (Israel). Journal of Archaeological Science: Reports. 2020;33:102447.

[34] Balkan-Atli N, Kuhn S, Astruc L, Kayacan N, Dinçe B, Balci S, et al. Göllü Dağ Survey 2010. Anatolia Antiqua. 2011;XIX:259–78.

[35] Milić M, Brown K, Carter T. Appendix 21.1: A visual characterization of the Çatalhöyük obsidian (on CD). In: Hodder I, editor. Substantive Technologies at Çatalhöyük: Reports from the 2000–2008 Seasons. Çatalhöyük Research Project Volume 9. London: British Institute at Ankara; Los Angeles: Cotsen Institute of Archaeology; 2013.

[36] Kolodny Y. Petrology of siliceous rocks in the Mishash Formation (Negev, Israel). Journal of Sedimentary Research. 1969;39:166–75.

[37] Singh BP. Chert breccia. Current Science. 2011;10:1402.

[38] Malinsky-Buller A, Aladjem E, Givol-Barzilai Y, Bonnes D, Goren Y, Yeshurun R, et al. Another Piece in the Puzzle—a New PPNA Site at Bir el-Maksur (Northern Israel). Paléorient. 2013;39(2):155–72.

[39] Agam A, Walzer N, Schechte HC, Zutovski K, Milevski I, Getzov N. Organized waste disposal in the Pottery Neolithic: A bifacial workshop refuse pit at Ein Zippori, Israel. Journal of Field Archaeology. 2016;41(6):713–30.

[40] Newcomer MH. Some qualitative experiments in handaxe manufacture. World Archaeology. 1971;3(1):85–104.

[41] Ekshtain R, Zaidner Y. Raw material exploitation at the Middle Paleolithic site of Nesher Ramla, Israel. Quaternary International. 2021.

[42] Crowfoot J. Notes on the flint implements of Jericho, 1936. Liverpool Annals of Art and Archaeology. 1937;24:35–51.

[43] Belfer-Cohen A. The Natufian Settlement at Hayonim Cave: A Hunter-Gatherer Band on the Threshold of Agriculture. Jerusalem: Hebrew University; 1988.

[44] Malinsky-Buller A, Aldjem E, Yeshurun R. Bir el-Maksur. A New Pre-Pottery Neolithic A Site in Lower Galilee. NEO-LITHICS. 2009;2:2–16.

[45] Goren-Inbar N., Herzlinger G., Grosman L., Belfer-Cohen A., & Agam A. (2021). Flint axes of Kaizer Quarry, Israel.Open Science Framework Online Repository 10.17605/OSF.IO/54327.

[46] Goring-Morris AN, Belfer-Cohen A. The Southern Levant (Cisjordan) during the Neolithic Period. In: Steiner M, Killebrew AE, editors. The Oxford Handbook of the Archaeology of the Levant (ca 8000–332 BCE) Oxford: Oxford University Press; 2014. p. 147–69.

[47] Taute W. Pre-Pottery Neolithic flint mining and flint workshop activities Southwest of the Dead Sea, Israel. In: Gebel HG, Kozlowski SK, editors. Neolithic Chipped Stone Industries of the Fertile Crescent (Proceedings of the First Workshop on PPN Chipped Lithic Industries). Berlin: ex oriente; 1994. p. 495–510.

[48] Finlayson B, editor Neolithic Settlements in the Greater Petra Region: A Synthesis. Palaeoenvironment and the Development of Early Settlements; 2016; Proceedings of the International Conferences at Sanliurfa 2012 and Aqaba 2013: Rahden/Westf.

[49] Richter T. Natufian and Early Neolithic in the Black Desert, Eastern Jordan. In: Enzel Y, Bar-Yosef O, editors. Quaternary of the Levant Environments, Climate Change, and Humans. Cambridge: Cambridge University Press; 2017. p. 94–101.

[50] Naveh D. PPNA Jericho: a Socio-political Perspective. Cambridge Archaeological Journal 2003;13(1):83–96.

[51] Mithen S, Finlayson B, Smith S, Jenkins E, Najjar M, Maricevic D. An 11,600 year-old pisé structure indicative of communal activity from the Neolithic of southern Jordan. Antiquity. 2011;85:350–64.

[52] Mithen SB, Finlayson B, Maričević D, Smith S, Jenkins E, Najjar M. WF16—Excavations at an Early Neolithic Settlement in Wadi Faynan, Southern Jordan. Stratigraphy, Chronology, Architecture and Burials. Oxford: Council for British Research in the Levant; 2018.

[53] Goring-Morris AN, Belfer-Cohen A. Houses and households: a Near Eastern perspective. In: Hofman D, Smyth J, editors. Tracking the Neolithic House in Europe. New York: Springer; 2013. p. 19–44.

[54] Gopher A, Barkai R. Sitting on the tailing piles: creating extraction landscapes in Middle Pleistocene quarry complexes in the Levant. World Archaeology. 2011;43:211–29.

[55] Finkel M, A. G, Ben-Yosef E, Barkai R, 16, 14–33.‏ A Middle paleolithic and neolithic/chalcolithic flint extraction and reduction complex at Mt. Achbara, Eastern Galilee, Israel. Archaeological Research in Asia. 2018;16:14–33.

[56] Schmidt K. Gobekli Tepe. A Stone Age Sanctuary in South-Eastern Anatolia. Berlin: ex oriente; 2012.

[57] Karul N, Buried Buildings at Pre-Pottery Neolithic Karahantepe. Türk Arkeoloji ve Etnografya Dergisi 2021;82:21–31.

