# Supplementary material for: Kaizer Hill (Modi‘in), a pre-pottery neolithic a quarry site – the terraced slopes
Source: PLoS One. 2022 Mar 24;17(3):e0265727. doi: 10.1371/journal.pone.0265727 (PMC8946753; doi:10.1371/journal.pone.0265727)

**S1 File**

# Detailed methodology and descriptions of the selected Rock Damaged Units (S1 text and S1_figs. 1-7)

Methodology of study, and the characteristics of each Rock Damaged Unit.

The rock damage features were defined as follows (plan view):

1. Straight quarrying front, linear (right angle front)
2. Drilling (voids)
3. Chiseling holes (groups of cup-marks)
4. Chiseling (cup-marks)
5. Widening of natural fissures

S1_ fig. 1: A symbol key describing the observed damage patterns on the 16 selected surface units.


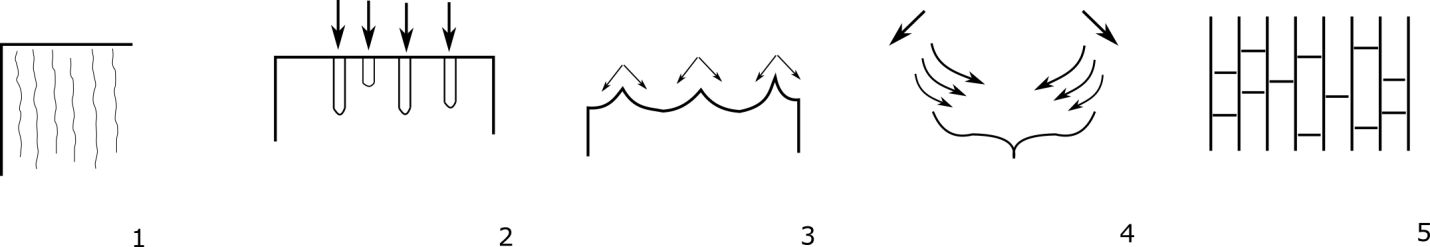


Detailed descriptions of each of the 16 sampled units:

**Unit 1**

About 7 m long, about 80 cm high.

Split into 3 sections.

Main features are Types 1 and 2.

**Unit 2**

Small boulder just east and in front of Unit 1.

Main feature are cuppings.

Square at bottom.

1.30 m wide.

Around 90 cm depth.

**Unit 3**

Just south of Unit 2.

Main feature is Type 1;

1.5 m wide.

Around 80 cm depth.

**Unit 4 (partially analyzed)**

About 12 m long.

Eroded frontal face.

Type 1 is main feature.

Depth is about 1m.

**Unit 5**

Elevation: 209 m

Length: about 3 m

Width: about 2.5 m

Depth: about 1 m

The main feature are cup-marks. Those range from 35cm length to 11cm maximum length, forming a quarrying front facing north.

**Unit 6**

Elevation: 254 m

Length (max): ca. 3m

Depth (max): ca. 1m

The main feature is Type 1 on the eastern face. There are also upper and lower “steps” as well as cup-marks. The largest cupmark is ca. 40cm in length, 22cm in width and 5cm deep, made by drilling.

There are horizonal, linear drill holes on the eastern face of the outcrop. Their maximum length is 65cm and they are about 3cm deep. The outcrop contains small, unexploited flint nodules.

**Unit 7**

Elevation: 243 m

Length: about 7 m

Width: about 2.5 m

Depth: about 50 cm

The major feature are drilled “channels”, occurying at defined intervals along the margins of the outcrop, creating a square-like pattern in plan, each ‘square’ is about 1m² and about 25cm deep.

**Unit 8**

Elevation: 243 m

Major feature is drilled “channeling”.

Length (max): 4 m

Width (max): 3.5 m

Depth (max) 45 cm

It is divded into one main segment and two smaller ones. The drilled segmenting is linear in fashion with defined spatial intervals so that pieces removed are square or “sub-square” in shape.

**Unit 9**

Elevation: 239 m

Length (max): 2.2 m

Width (max): 1.1 m

Depth (max): 60 cm

M-C (depth, max): 20 cm

There is alearly defined 90° angle between the very flat upper surface and the quarrying front on the NE face. There are scattered horizontal drilling holes on the whole quarrying surface.

**Unit 10**

Elevation: 248 m

Length (max): 5.5 m

Width (max): 2.4 m

Depth (max): 50 cm (both upper and lower surfaces)

The major features are a complex pattern of drilled channels on the upper surface. It exhibits a quarried vertical surface, which is divided into one large and two smaller rectangular or semi-rectangular segments.

S1_fig. 2: Unit 10; on the left: a general view of Unit 10; on the right: a close up view.


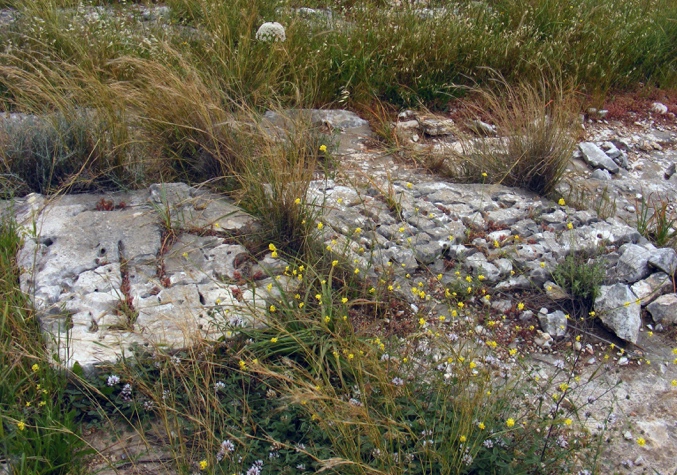

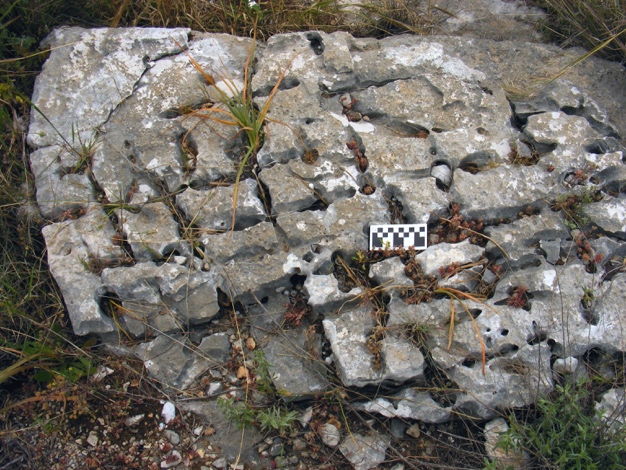


**Unit 11**

Elevation: 239 m

Length: 2.5 m

Width: 1.4 m

This feature exhibits a series of cup-marks on the upper surface which flint nodules systematically removed from north to south, where there are still flint nodules in situ. There are also a number of drilled channels beginning at the eastern wall and moving inward. They are more or less at equal intervals from one another.

S1_fig. 3: Unit 11


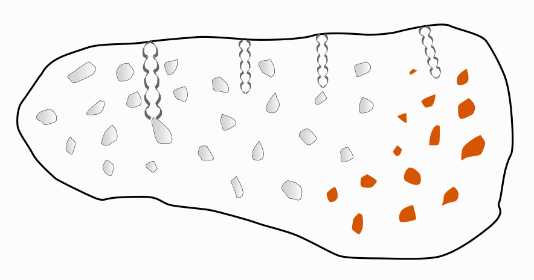


**Unit 12**

Elevation: 239 m

Length: 4.5 m

Width: 4.0 m

Depth: 1.1 m

A very large sub-square surface that exhibits a large amount of in situ flint nodules. It appears that only a few were quarried out, leaving cup marks. The most striking feature of this unit is an elongated flint nodule (48cm X 9 cm) that the quarrymen attempted to extract but left in situ. The nodule has a long-drilled channel leading to it, and a series of quarried (vertical) channels around it.

S1_fig. 4: Unit 12: The eastern vertical face.


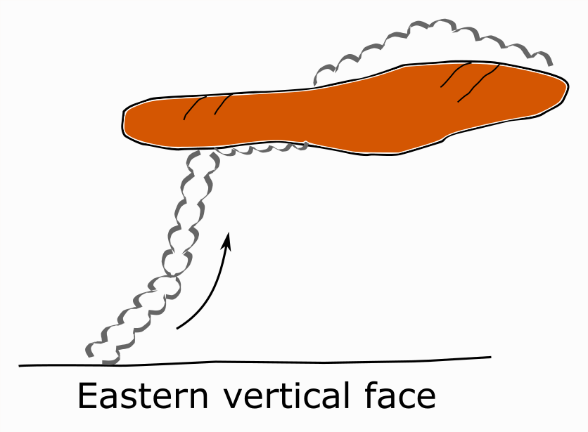


**Unit 13**

Elevation: 239 m

Length: 2m

Width: 1.70 m

Depth: 1.10 m

A relatively deep and isolated feature with a large, elongated flint nodule in the center of the upper surface. The quarrymen approached it from the southern end and towards the nodule by a drilled channel but left it in situ. The other major characteristic of this feature is the clear quarrying channels (about 10) that are situated in association with a cup mark. Four or five of these are at equal distances from one another on the SW surface.

**Unit 14**

Elevation: 242 m

Length: 2.70 m

Width: 2.10 m

Depth: 40 cm

A large, low-lying flat feature that exhibits a widening of a natural fissure in the stone. As the quarrymen were following the natural fissure, the drilled channels are linear but not parallel as observed in other units where channels were artificial, not associated with natural fissures.

**Unit 15**

Elevation: 224 m

Length: 3.40 m

Width: 1.70 m

Depth: 70 cm

The main feature of this unit is a large number of very narrow drilled channels particularly on one of the units sections. Only a few of these are associated with cup marks.

S1_fig. 5: Unit 15.


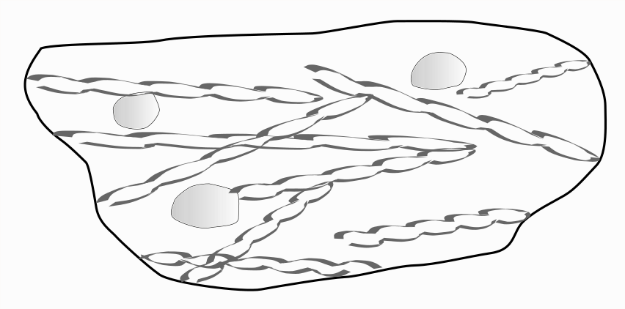


There are at least two very deep and wide fissures that were further widened by quarrying. One of these resulted in the complete separation of one surface section and a partial separation of a second.

**Unit 16**

Elevation: 242 m

Length: 2.30 m

Width: 2.20 m

Depth: 60 cm

The outcrop has several quarrying fronts. Running along the face of the Northern end, there are drilled quarrying channels that are deeper than most of those observed in the other units described above.

S1_fig.6: Plan view of north-east wall (a cast was made of it)


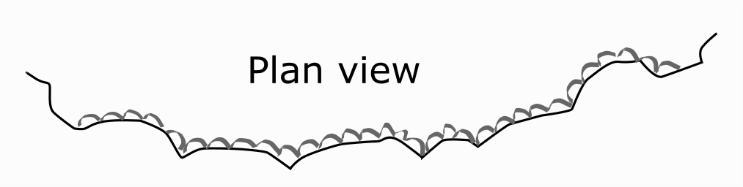


In addition, on the upper surface of the unit, there is a shallow quarrying front where the quarrymen worked in a SE direction (towards the top of the hill). This front runs perpendicular at a 90° angle to the main vertical front. The quarrying stoped at the meeting point of the two fronts.

S1_fig.7: A 90^0^ angle quarrying front.


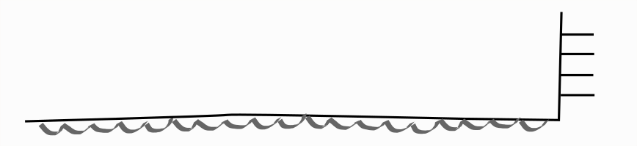

Supplement: S1 File — (DOCX) [file pone.0265727.s001.docx]
