# Supplementary material for: Kaizer Hill (Modi‘in), a pre-pottery neolithic a quarry site – the terraced slopes
Source: PLoS One. 2022 Mar 24;17(3):e0265727. doi: 10.1371/journal.pone.0265727 (PMC8946753; doi:10.1371/journal.pone.0265727)
Supplement: S1 Text — (DOCX) [file pone.0265727.s003.docx]

# S1 Text - KAIZER - Detailed Tool Typology

## Surface (T0-T3)

ENDSCRAPERS (N=24)

On flake – 11 items. 2 on CT flakes, one of which is steep; 1 complete, rounded, semi-carinated; 1 is askew; 3 are actually micro-endscraper, of which two are on small primary flakes; 1 is regular and complete; 2 are broken of which one is with double patina; 1 is on a burnt flake.

On retouched flake – 5 items. 1 is retouched on both laterals, with patinated cortex; 1 on alternately retouched flake; 2 are broken, one is actually a-typic thumbnail on CTE, alternately retouched; 1 on a primary flake CTE, double patinated, the lateral retouch is irregular.

On retouched blade – 2 items, both broken.

Rounded – 1 item.

Carinated (mini) -2 items. Both are micro, 1on flake with **double patina** and 1 on a retouched blade.

Varia – 2 items. 1 on a retouched flake; 1, with double patina, semi-steep.

Bit – 2 items. 1 probably on a flake.

BURINS (N=25)

Dihedral – 2 items. Transversal (according the striking platform orientation) or straight (symmetry of flake), on primary, hammerstone/CTE flake; 1 *dejete*, on flake.

On break – 3 items. 1 on a break, ventral; 2 items, both a-typique, one probably belongs to the ‘borer’ category.

Transverse – 3 items. 2 on lateral retouch, on flake; 1 on a break, on a thick flake.

On Truncation - 10 items. 6 on a straight truncation, five of which are on flakes, two of which are thick. One of them is double patinated, the truncation is actually a previous endscraper retouch, the burin blow is ventral. Another is tiny, on a primary flake, one on an alternately retouched blade; 3 on a concave truncation, one is on a fragment, another is on a flake and the third is on a CTE flake; 1 oblique on a tiny, broken bladelet.

Double, dihedral – 3 items. 1 *dejete* and on a break, on a primary flake; 2 are straight dihedral and on break, both on flakes, one of which is rather thick.

Double, mixed – 1, on a break and on a concave truncation, on a CTE flake.

Varia – 3 items. All are on a straight truncation, ventral. One on a blade, one on a flake with double patina and one on a primary flake.

BORERS (N=111)

Borer – 3 items. 1 on a flake with a symmetrical end, half of the laterals converging to a point. 2 a-typic, 1 on a primary flake.

Heavy borer/bec – 2 items, 1 on a retouched flake; 1 on a core fragment.

Spike – 19 items. 6 on flakes; 2 transverse on a flake; 2 on retouched flakes, one of which is double patinated, retouched alternately all around; 2 on blades, broken, with some alternate retouch; 1 is on a retouched blade, burnt and broken; 1 on an inversely retouched flake; 1 on a fragment; 1 on a primary thick flake (chunk?); 1 on a core fragment; 1 on a CTE flake; 1 on a thinning flake.

Awl – 9 items. 5 on retouched flakes, one alternately retouched and another is broken, with a characteristic break scar and double patinated; 1 on a double patinated flake; 1 on a bifacial CTE; 1 on a short blade, with alternate retouch; 1 on a fragment.

Bec - 28 items. 6 on flakes, two of which are double patinated; 4 on a primary flake, two of which are quite thick, and one is perhaps a double bec; 2 on retouched flakes; 2 on a retouched CTE flake; 2 on a CTE flake, perhaps a double ‘borer; 1 on a CTE fragment, with double patina; 4 on alternately retouched flakes; 2 a-typique, one on a CTE and another on a flake; 2 on a blade, small and broken; 1 on a broken primary blade; 1 on what was previously a carinated endscraper; 1 on a burnt flake.

Double Borers/Becs - 43 items. 2 on flakes with quite an intensive retouch; 2 on double patinated flakes; 2 on a primary flake, one of which is quite massive; 1 on a fragment; 5 are double mixed (=thick and flat), one of which is on a double patinated flake; 3 are triple becs; 3 massive double becs; 1 double flat; 12 are in-between multiple spikes/denticulate, nine flakes, one primary flake, three of them quite thick with ‘bifacial’ lateral retouch/use, two double patinated, used also as a hammerstone, one on blade, one on bladelet, one on a primary flake ; 4 are a combination of spike-bec, on flakes, two with a lateral retouch; 2 are a combination of awl-bec, one with ouble patina, another on a ‘Levallois’ blade**;** 4 awl (alternate retouch)-spike, three on flat flakes and one on regular flake**.**

Varia – 7 items. 1 on blade, 1 on bladelet, 1 on a primary flake; 2 on a flat flake; 2 on regular flakes, one with an inverse retouch.

BACKED PIECES (N=7)

Backed fragment – 7 items. All are very irregular, one is on a primary blank.

TRUNCATIONS (N=11)

Most of those are on their way to become a borer-family tool, or simply do not fit-in within the strict definitions of what is a borer/aw/bec.

Concave – 3 items. All on flakes, one with intensive retouch, two are quite thick, seem to belong to the ‘borer’ category, intensively used.

Straight – 8 items. 1 on a flake; 2 on retouched flakes of which one is primary; 1 with a straight truncation on a bifacial CTE; 1 on an alternately retouched flake; 1 is ventral on a flake; 1 cortical; 1 very intensively retouched, nearly a semi-steep scraper.

NOTCHES & DENTICULATES (N=29)

Also here quite a few of the items are borers in the making, i.e., of the borer family.

Single notch - 20 items. 1 on a broken CTE blade; 2 on blades, both broken with lateral retouch on the opposed side; 4 are on flakes, one of which is big and double patinated; 2 are on primary flakes, one is big, battered and bashed, the other is double patinated; 1 on a steep retouched flake, with double patina; 3 are on simple retouched flakes; 1 is on a CTE flake, also double patinated; 5 are on fragments, one is cortical, two are perhaps broken ‘borers’; 2 are ventral, on a fragment and a flake; 1 is on a cherty flake.

Multiple notches – 6 items. 1 bi-lateral, on a fragment of a blade; 2 on flakes, both actually of the borer family; 3 are of the spoke-shave variety, a-typique, on fragments and one of them burnt.

Denticulate – 3 items. 2 are on flakes**;** 1is on a primar**y** blade.

RETOUCHED ITEMS (N=26)

Retouched flake – 18 items. 6 with regular retouch, one is burnt and another with double patina; 4 with inverse retouch; and 3 with alternate retouch, one of these is on a ‘bifacial’ CTE.

Retouched blade – 3 items. All broken.

Retouched fragment – 5 items. One is cortical.

MULTIPLE TOOLS (N=29)

Endscraper+Burin – 4 items. 1 a-typique endscraper and a burin on a break, on a flake; 1 is a burin on concave truncation, which might as well be a massive bec; 1 actually triple (burin on a break, transverse burin on retouch and semi-steep endscraper) on a flake double patinated; 1 actually a double endscraper, the ‘burin’ is accidental, on double patinated flake.

Endscraper+Borer –14 items. 2 are endscraper/bec, one on a thick flake and one on a CTE flake; 1 is an endscraper/awl on a double patinated flake; 1 is an endscraper/spike, on a flake, the endscraper may also be actually of the borer family; 1 on a thick, primary flake, can be also assigned as multiple borer to the borer category; 1 on a primary flake, obverse to each other with some lateral retouch; 1 on thick primary flake at opposed ends; 2 on double patinated flake, obverse to each other, with some lateral retouch; 2 on a broken flake; 1 on a retouched blade; 1 actually a triple tool (two borers and an endscraper), on a flake; 1 on a primary CTE flake.

Endscraper+Truncation – 1item, straight, on a fragment with double patina.

Burin+Bec – 1item, dihedral, on a flake.

Burin+Notch – 1item, burin on break with retouch arresting the burin removal and a deep notch at the opposite, on a blade;

Borer+Truncation – 1item, on a primary flake.

Borer+Notch – 5 items. All are on flakes, one is a bec, on a thick, battered flake, another is an awl, two can be assigned to a variant of the borer category.

Borer+Denticulate – 1item, on a thick flake, sort of a heavy duty tool, a varia.

Varia (double tool) – 1item, on a primary fragment, transverse burin and a bec.

BIFACE (N=46)

VARIA (N=32)

This category includes: 1item which is a burin of sorts, double dihedral on break, on a flake; 1 variant of a bec with bifacial modification at the base, more like a push-plane; 1 inversely retouched flake; 1 alternately retouched flake; 2 *piece esquillee*, both are bifacial, one on a small burnt flake; 1 retouched bifacial CTE; 1 small core with a spike like edge, and some retouch; 6 ‘varia’ including one on a CTE flake and one with double patina; 11 are simply ‘varia’ including 3 primary flakes, 1 thinning retouched fragment; 1 microburin; 2 on a primary flake, served both as a borer and a hammerstone; 4 battered –cum-retouched items, two on a thick primary flake, one on a chunk, and one on a thick primary blade, i.e. a variant of heavy duty tools

## T2 (excavation) (N=144)

ENDSCRAPERS (=2)

On a flake – 1item, on a CTE primary flake.

Carinated – 1item, frontal, core-like.

BURINS (N=9)

Dihedral – 1item, straight on a small flake.

Polyhedral – 2 items. 1 ventral on a primary flake and 1 regular, on flake.

On a break – 3 items. 1 on a massive battered flake; 1 on a retouched, battered flake; 1 on a primary flake, ventral.

Transverse – 1item, on a flake.

Varia – 2 items. Both seem accidental, on a break, on flakes.

BORERS (N=60)

Borer – 12 items. 1 ‘varia’ on a burin spall, of blade proportions; 2 ‘heavy duty’ on flakes, one is a primary flake, with double patina; 2 on a blade; 1 on a retouched blade; 2 on a flake, one is proximal, as if a bifacial tang, complete; 1 on a retouched flake, with double patina, burnt; 1 alternate on a fragment; 2 alternate, one of them on a flat flake.

Awl – 8 items. 3 are on ‘flat’ flakes, two of them with alternate retouch; 2 on flakes; 1 on a fragment; 2 alternate, on a flake;

Spike – 20 items. The ones not described are on regular flakes. 1 on a primary blade; 1 on a retouched blade/bladelet; 5 are on ‘flat’ flakes; 2 are burnt; 3 are multiple on a flake, one with double patina; 1 on a retouched flake, ventral; 2 on a fragment.

Bec – 3 items. 1 massive, on a flake and 2 are on retouched flakes.

Double borer – 17 items. 1 is triple on a ‘flat’ flake, with double patina; 4 are double spikes, one on a tiny fragment, one on a primary flake and two on flakes, alternate; 2 are actually multiple spikes/denticulates on **‘**flat’ flakes; 4 are double becs on flakes, one of them a ‘flat’ one with double patina; 3 items are a bec + spike, one is on a primary ‘flat’ flake, the other is on a fragment and the third is ‘varia’ on a flake; 1 is a borer-spike on a flake; 2 are ‘varia’, one on a ventrally retouched flake and the other on a broken blade/bladelet.

BACKED PIECES (N=1)

Backed blade – 1item, primary.

TRUNCATION (N=1)

Oblique – 1item, on a flake.

NOTCHES & DENTICULATE (N=22)

Single notch - 11 items. 1 on a retouched ‘flat’ flake, 4 on fragments, two of them ventral and most probably of the borer category; 1 on a burin spall; 1 on a CTE; 4 on flakes, one of them burnt.

Multiple notches – 6 items. All on flakes - 3 are dorsal, 2 are ventral, 1 is alternate. It seems that they mostly relate to the borer category.

Denticulate – 5 items. 1 is fine, on a primary flake; 3 on flakes, most probably related to the borer category; 1 is on a blade.

RETOUCHED PIECES (N=17)

Retouched blade –4 items. All are broken. 1 with an alternate retouch; 1with an inverse retouched; 2 seem on the verge of use-wear.

Retouched flake – 11 items. 2 massive, one of them primary; 2 are on ‘flat’ flakes; 2 are regular, one of them broken; 5 items seem on the verge of ‘use-wear’.

Retouched fragment – 2 items. 1 with cortex, the other with semi-steep retouch, double patina and burnt.

MULTIPLE TOOLS (N=7)

Endscraper+Burin – 1item, a heavy duty scraper on a retouched, large, primary flake and a transverse burin.

Burin+Borer – 2 items. 1 burin on truncation (varia), on a small CTE flake and a bec; 1 transverse burin and a borer, of the heavy duty category, on a flake with double patina.

Borer+Notch – 4 items. 3 are becs, one on a primary flake, burnt, one on a ‘flat’ flake and the third is on a primary flake with alternate retouch and double patina; 1 is a spike, on a burin spall (?)

MICROLITH (N=2)

Helwan bladelet – 1item, broken.

Retouched bladelet – 1item, medial part, alternate retouch.

BIFACE (N=9)

VARIA (N=1~~4~~)

Six can be assigned to ‘varia’ in the borer category - one is a massive bec on a denticulate flake, one is a fragment and four are on flakes, one of which is retouched and double patinated; 1 item is a heavy duty scraper on a huge primary flake; 1 is a primary flake with a sheen; 1 is a transverse scraper on a primary flake; 1 battered flake; 1 *piece* *esquillee*-like, with bifacial retouch/battering, on a flake; 3 ‘varia’ on flakes.

## T3 (excavation) (N=65)

ENDSCRAPERS (N=6)

On flake – 1item, a-typique.

Carinated – 1item, on a thick flake.

Semi-carinated – 1item, on a blade.

Bit – 2 items.

Double – 1item, on a primary flake, mixed.

BURINS (N=4)

On a break – 1item.

On a straight truncation – 1 item, on a thick flake.

Carinated – 1 item, varia.

Double, on a break – 1 item.

BORERS (N=19)

Borer – 2items. 1 on a broken flake;1 snapped off, on a retouched flake.

Awl – 3 items. 1 on a retouched burin spall, 1 on an alternately retouch flake, 1 on a ‘flat’ flake.

Bec – 4 items. 1on a ventrally retouched flake, 1 on a retouched flake, 1 on a blade, 1 on a thick CTE flake.

Spike – 2 items. 1 on a retouched fragment, 1 on a flake.

Double – 8 items. 1 double bec on a flake; 4 bec-spike, one on a blade, one on a primary, alternately retouched flake; 1 spike-spike; 2 multiple spikes on flakes, one burnt.

NOTCHES & DENTICULATES (N=3)

Single notch – 1item, on a ‘flat’ flake with inverse retouch, most probably a broken borer.

Multiple notches – 1 item, on a flake with some cortex.

Denticulate – 1 item, on a large flake, most probably belongs to the borer category (some characteristic snaps).

RETOUCHED ITEMS (N=6)

Retouched flake – 3 items. One on a ‘flat’ flake, perhaps a broken borer.

Retouched flake, inverse – 3 items, 2 on ‘flat’ flakes, 1 on a regular flake.

MULTIPLE TOOLS (N=6)

Burin+Notch – 3 items. One burin is dihedral straight, one on a break, one transverse on lateral retouch.

‘Borer’+Notch – 2items, 1 perhaps a multiple bec, 1 a spike/awl on blade.

Sickle+Spike – 1item. A medial fragment, retouched, the spike is on the opposed lateral.

BIFACES (N=12)

VARIA (N=9)

*Piece esquillee* – 1 item, on flake; Varia – 8 items. 1 ‘burin’ on a core; 1 retouched primary flake; 1 retouched flake; 1 inversely retouched flake; 1 burnt, modified fragment; 1 backed fragment; 1 notch or a retouch on a broken ‘flat’ flake; **1** retouched CTE.

## Surface (above building) (N=316)

ENDSCRAPERS (N=28)

On flake – 7 items. Most of them intensively retouched. 2 are primary flakes, one of which is a CTE (of the bifacial variety). All are flat.

On retouched flake – 1 item, broken with alternate, continuous retouch.

Thumbnail – 2 items, one of them cortical.

Nosed, flat – 4 items, one with little cortex – related to the “borer” category for sure.

Nosed, thick – 2 items, both on flakes, one with double patina, sort of a ‘Levalois’ flake.

On blade – 1 item, short.

On retouched blade – 2 items, one is actually a primary blade, the other has a little cortex.

Carinated – 2 items, both frontal, both on primary flakes.

Denticulated – 1 item, on a primary flake – can be assigned to the “borer” category as multiple spikes.

Double – 3 items, 1 is mixed on a flake, 1 alternate flat on a flake, and 1 is double flat on blade.

Varia – 3 items. 1is a thick scraper on flake, 1 is on a flake with some lateral retouch and 1 is actually a transversal/fan scraper on a retouched flake.

BURINS (N=12)

Dihedral – 1 item, *d’angle*, on a double patinated flake.

On break - 3 items, 1 on a CTE/Core fragment, 1 on a thick primary flake with some retouch, 1 on a flake CTE with double patina.

Transverse **-** 2 items, on lateral retouch. 1 is a thick blade with some retouch, the burin scar cancels out an endscraper. 1 is on an inversely retouched, broken, double patinated flake.

On concave truncation – 3 items, all are on flakes, 2 actually can be assigned also to the “borer” category. 1 is quite accidental with irregular lateral retouch.

Double on truncation – 1 item with some irregular retouch/use wear.

Double mixed – 2 items, 1 on a thick CTE, nearly a core, 1 on a primary blade (can be assigned to the ‘borer” category).

BORERS (N=114) Actually, there are no ‘classic’ borers. 10 items are modified on “flat” flakes.

Spike – 15 items. Most of the retouch is alternate to create the working edge, though 5 items are only with obverse retouch; 1 on a primary flake. 1 item on a burnt and retouched flake; 1 on a thick flake with cortex; 1 on a broken blade; 2 items are with double patina.

Awl – 9 items, all are on flakes, 3 of them with an alternate retouch.

Bec – 23 items: 3 artefacts are with alternate retouch, 9 with inverse retouch and 10 with an obverse one. 1 with a shining spot (inversely), 4 are on primary flakes while 2 are with some cortex, 4 items are with double patina including one which is on a ‘bifacial’ CTE, 1 item is burnt.

Double/Multiple – 58 items. Most of them are with alternate or inverse retouch creating the working bit. 13 are with double patina, 8 are primary flakes, and 3 other with little cortex, 3 burnt flakes, 12 are on retouched flakes, 1 on a retouched blade. Most of them are of a bec + spike variety, bec + bec are rare and there are 2 multiple spikes on small retouched flakes.

Varia – 9 items. 1 on a burnt flake and 2 on primary flakes.

BACKED PIECES (N=3)

Backed fragment – 3 items. 1 with bipolar retouch and an alternate one on the opposed lateral. 1 seems to belong to the ‘borer’ category.

TRUNCATION (N=2)

Straight – 2 items, both on alternately retouched flakes.

NOTCHES & DENTICULATES (N=35)

Single notch – 23 items. 1 on chunk, 2 thick flakes, 2 on blades, 1 primary flake, 1 with double patina. All very irregular – mostly suspected of belonging actually to the ‘borer’ category.

Multiple notches – 3 items. 2 bilateral on broken blades, one of which is with some retouch on its distal end, as if an endscraper; 1 on a retouched blade with double patina.

Denticulate – 9 items. Also, most of these items relate somehow to the ‘borer’ category. 1 item as if a ‘spoke-shave’; 1 is on a primary flake.

RETOUCHED (N=27)

Retouched flake –12 items. All kind of ad-hoc items. 2 are double patinated; 1 burnt with a steep retouch; 1 on a cortical CTE; 1 on a ‘bifacial’ CTE; 2 primary flakes, one inversely retouched and the other seems as if a broken borer.

Retouched blade – 9 items. 1 is a primary blade; 2 are with semi-abrupt retouch; 1 is double patinated.

Retouched fragment – 6 items. 1 on a ‘bifacial’ CTE; 1 item is double patinated; 1 item is inversely retouched.

MICROLITHS (N=20)

Retouched bladelet – 2 items, both broken with an ad-hoc retouch.

MULTIPLE TOOLS (N=30)

Endscraper+Burin – 2 items. Both are semi-steep scrapers and burins on a break, one with double patina and the other is on a primary flake.

Endscraper +Borer – 9 items. Most of them are endscraper-bec, but there are also an endscraper-spike and a denticulate scraper + bec on an alternately retouched flake which most probably can be assigned also to the borer category. 1 specimen is a triplet – with 2 becs and the endscraper is ventral. 2 items are of the heavy-duty variety, both on primary flakes; 2 additional are on primary flakes.

Endscraper+Notch – 2 items. Both are on thick primary flakes, one of them is a ventral nosed endscraper.

Burin+Borer - 6 items. 1 on a concave truncation/notch on a small flake; 1 dihedral/transverse on a fragment, the borer bit snapped off; 1 a burin on a break and a double bec on a flake; 1 burin on oblique truncation and a spike, on a retouched flake; 1 ‘accidental’ burin and spikes; 1 a burin on a break and a bec, on a ‘bifacial’ CTE.

Burin+Notch – 1 item, dihedral burin, more like a broken ‘borer’ category item, on a fragment

Truncation+Notch – 2 items, both truncations are straight, one on a primary flake and the other is with double patina.

Borer+Truncation – 8 items. Most ‘borers’ are spikes. 1 with double patina; 3 on retouched flakes, 1 with some cortex. Most of them are actually multiple borers.

BIFACES (N=34)

VARIA (N=29)

*Piece esquillee* – 2 items, one on both faces, one is a-typique.

Varia – 27 items. 11 are failed, exhausted items of the ‘borer’ category; 2 are sort of a-typique endscrapers, 1 flat and 1 carinated; 1 arrowhead/borer; 1 probably a biface fragment; 4 as if a ‘chisel’, one of them cortical and burnt; 1 with bifacial ‘retouch’ on a CTE.
